# Optimising digital clinical consultations in maternity care: a realist review and implementation principles

**DOI:** 10.1136/bmjopen-2023-079153

**Published:** 2024-11-01

**Authors:** Catrin Evans, Georgia Clancy, Kerry Evans, Andrew Booth, Benash Nazmeen, Candice Sunney, Mark Clowes, Nia Jones, Stephen Timmons, Helen Spiby

**Affiliations:** ^1^School of Health Sciences, University of Nottingham, Nottingham, UK; 2 School of Health and Related Research, University of Sheffield, Sheffield, UK; 3 School of Allied Health Professionals and Midwifery, University of Bradford, Bradford, UK; 4 Notitngham Maternity Research Network, University of Nottingham, Nottingham, UK; 5 School of Medicine, University of Nottingham, Nottingham, UK; 6 Business School, University of Nottingham, Nottingham, UK

**Keywords:** Quality in health care, Maternal medicine, Systematic Review, Telemedicine, Health Equity, Organisation of health services

## Abstract

**Abstract:**

**Objectives:**

The COVID-19 pandemic has led to increased use of digital clinical consultations (phone or video calls) within UK maternity services. This project aimed to review the evidence on digital clinical consultations in maternity systems to illuminate how, for whom and in what contexts, they can be used to support safe, personalised and equitable care.

**Design:**

A realist synthesis, drawing on diverse sources of evidence (2010–present) from OECD countries, alongside insights from knowledge user groups (representing healthcare providers and service users).

**Methods:**

The review used three analytical processes (induction, abduction and retroduction) within three iterative stages (development of initial programme theories; evidence retrieval and synthesis; validation and refinement of the programme theories).

**Results:**

Ninety-three evidence sources were included in the final synthesis. Fifteen programme theories were developed showing that digital clinical consultations involve different mechanisms operating across five key contexts: the organisation, healthcare providers, the clinical relationship, the reason for consultation and women. The review suggests that digital clinical consultations can be effective and acceptable to stakeholders if there is access to appropriate infrastructure/digital resources and if implementation is able to ensure personalisation, informed choice, professional autonomy and relationship-focused connections. The review found relatively less evidence in relation to safety and equity.

**Conclusions:**

Due to the complexity of maternity systems, there can be ‘no one-size fits all’ approach to digital clinical consultations. Nonetheless, the review distills four ‘CORE’ implementation principles: C—creating the right environment, infrastructure and support for staff; O—optimising consultations to be responsive, flexible and personalised to different needs and preferences; R—recognising the importance of access and inclusion; and E—enabling quality and safety through relationship-focused connections. Service innovation and research are needed to operationalise, explore and evaluate these principles, particularly in relation to safety and equity.

**PROSPERO registration number:**

CRD42021288702.

**STRENGTHS AND LIMITATIONS OF THIS STUDY**

- The realist methodology moves beyond a descriptive focus on barriers or facilitators of digital clinical consultation, identifying core implementation principles that can inform future research and service innovation.
- The combination of purposive and comprehensive evidence searching means that the findings (programme theories) are underpinned by a large number of relevant evidence sources and are likely to be applicable across a range of maternity settings.
- The project has been influenced by knowledge user insights at every stage, from question formulation to development of recommendations.
- The diversity of potential knowledge users across a complex and large maternity system means that, in spite of efforts for inclusive involvement, relevant perspectives or contexts may have been missed.
- The inclusion criteria prioritised UK evidence sources; however, a wide range of international literature is nonetheless included.

## Introduction

 The COVID-19 pandemic saw the rapid introduction of hybrid models of maternity care in which some in-person contacts were replaced with remotely delivered care. Post pandemic, there is a need to consider whether and how virtual consultations should continue to feature within maternity care pathways.

Remote care has a very diverse nomenclature.^1^ In this paper, we draw on the work of Griffiths *et al*^2^ and refer to remote care as ‘Digital Clinical Consultation’ (DC-CON), defined as: “synchronous telephone or video consultations involving direct interaction between a service user and a maternity healthcare professional. It has two-way functionality and can be initiated by either party. It may be linked to, or complemented by, other digital technologies within the maternity care pathways.” This definition links the consultation to the systems within which it operates.

There is a small, heterogeneous, but accumulating, evidence base to suggest that hybrid models of maternity care can achieve equivalent clinical and patient satisfaction outcomes.^3^,^11^ Less is known however regarding DC-CON’s potential impact on harms and health equity, with experts calling for caution and more research prior to widespread rollout.^39 12^,^17^

In the UK, good practice guidance was issued during the pandemic to support the use of DC-CON.^18^,^20^ Since then, anecdotal evidence suggests that DC-CON remains a feature of maternity care, but that practices are highly variable within and between different healthcare services. A large-scale maternity transformation programme is underway in the UK, with digital transformation as a key component.^21^,^23^ It is likely that an element of DC-CON is here to stay; therefore, guidance is needed to support implementation.^24 25^

### Aim

This paper reports the findings of a realist evidence synthesis that aimed to illuminate how DC-CON can work to support safe, personalised and appropriate maternity care (from pregnancy to 14 days post partum), and to clarify when it might be most appropriately used, for whom, how and in what contexts. A previously published protocol provides further detail of the review’s rationale and methods (see online supplemental file 1).^26^ The review is registered with Prospero: CRD42021288702.^27^

## Methodology

Realist reviews draw on diverse evidence sources to explore complexity and causality in healthcare.^28^,^30^ Drawing on a realist ontology, the aim is to generate theoretical understandings (referred to as ‘Programme Theories’ (PTs)) of how healthcare interventions (such as DC-CON) work and why their outcomes may vary in different contexts.^31 32^ PTs are expressed using a *C*ontext-*M*echanism-*O*utcome (C-M-O) heuristic. The responses of actors to different intervention resources are referred to as ‘mechanisms’.^33^ Interventions are implemented through a range of different contexts. These differences in context can cause different mechanisms to be activated and lead to variation in outcomes.^34 35^ Within a complex healthcare system such as maternity care, innovations are implemented through many levels of context, involve many groups of actors and are associated with multiple mechanisms.^36^ Hence, PTs need to be able to incorporate multilevel and intersectional phenomena.^37^ A glossary of terms used in realist enquiries can be found in online supplemental file 2.

### Patient and public involvement

Following a regional research prioritisation exercise,^38^ the review was informed from the outset by two groups of knowledge users.^39^ The first was a community organisation and service user group (COSU-G). This comprised 13 women, recruited from 3 organisations to ensure that it represented a diversity of experience and identities: (1) through a local patient and public research network (the Nottingham Maternity Research Network from whom one member—CS—became a project coinvestigator), (2) the National Autistic Society, and (3) via Sister Circle (an organisation working with women experiencing complex social disadvantage in a linguistically and ethnically diverse area of London). The second was a healthcare professional group (HCP-G) comprising midwives and obstetricians working in different roles across the UK maternity system (n=26). Representatives of the groups were recruited through direct invitation to the community organisations and through open invitations to participate (disseminated through professional email lists and social media). The knowledge user groups contributed to the project at every stage through online meetings and an in-person workshop. Their input is reported according to the GRIPP2 checklist^40^ (see online supplemental file 3) and is described in more detail below and in a recently published paper.^41^ In this review, knowledge user expertise and lived experiences contributed to the generation, refinement and sense checking (interpretation) of the emerging theoretical insights. As such, their insights helped to shape and illuminate the review findings and the review process.^41^ We did not undertake formal (primary) research to generate new evidence as this was beyond the scope of the current project. The review team also received guidance and advice from a ‘Project Advisory Group’ (n=9) comprising senior midwives and obstetricians with strategic leadership roles related to quality, maternity digital transformation and equity, diversity and inclusion. In addition to the regular input of these groups, three additional consultation workshops were undertaken with women (n=22) in the final phase of the project. Online supplemental file 4 provides further details of the knowledge users.

## Methods

The realist synthesis was conducted in three iterative phases^31^ (see online supplemental file 5 for a flowchart), each drawing on two main sources of evidence: (1) literature (published and unpublished research, health and safety investigation reports, audit, evaluation, theory, policy and guidance) and (2) knowledge user expertise and insights. The review followed the Realist and Meta-narrative Evidence Synthesis Evolving Standards quality procedures^30 37^ and publication standards (online supplemental file 6).^42^ Evidence searches were conducted by an experienced information scientist. To ensure rigour, every stage of the review was undertaken by two or more team members.

### Phase 1: development of initial programme theories

This phase involved the development of initial programme theories (IPTs) proposed to characterise DC-CON implementation. It involved exploratory searches for evidence (2010–present) drawing on: (1) sources suggested by the team and knowledge user/advisory groups, (2) Google Scholar and (3) searches in three bibliographic databases guided by the BeHEMoTh approach to searching for theory (behaviour of interest, health context, exclusions, models or theories).^43^ Initial consultations with the knowledge user groups strongly emphasised the need for IPTs to incorporate equity and diversity as key dimensions.^41^ This emphasis was incorporated into a purposive sampling framework to help prioritise relevant papers. Online supplemental file 7 sets out the full search and study selection approach and final list of evidence sources (n=49) included for IPT development.^712 14 15 18^,^20 22 23 44^ The searches identified three mid-range theories that contributed concepts to IPT development: Candidacy,^45^ Burden of Treatment^68^ and Normalisation Process Theory (NPT).^84^ These had direct relevance to the review’s focus on digital health implementation processes and could incorporate equity and diversity concerns. IPT development was also informed by a conceptual framework with implementation principles entitled ‘*Planning and Evaluating Remote Consultation Services*’ (PERCS).^51^ This framework helped to clarify the macrolevel, mesolevel and microlevel context dimensions that overlap and interact with DC-CON within a complex and dynamic healthcare system to produce variable outcomes, including equity/social justice. Table 1 elaborates the rationale for, and application of, the theories and conceptual framework. The analysis in phase 1 drew on thematic synthesis techniques, combined with abductive and retroductive theorising^85^ to propose 13 IPTs (see online supplemental file 8).

**Table 1:** Utilisation of mid-range theories and conceptual framework to inform programme theory development

Theory	Brief summary	Rationale for consideration in IPTs	Select examples of application for IPT development
Normalisation Process Theory (NPT)^84^	This is a theory of the work that individuals and groups undertake to enable an intervention or change to become ‘normalised’ and sustained. It has four main components: coherence (meaning or sense-making); cognitive participation (commitment or engagement); collective action (work done to enable the intervention to happen); and reflexive monitoring (formal and informal reflection and appraisal of the benefits and costs of the change).^84^	NPT has been widely used to investigate and theorise processes of change, including those related to the introduction of new technologies. It has most often been applied to understand the response of staff and organisations in processes of health system change, but can also be used to understand patients’ actions and perspectives.^66^ The focus on understanding sustainability of a change matches closely to a key concern of the review.	Some evidence sources identified that staff valued the relationships they developed with their patients and that DC-CON could be resisted as a result of concerns about disrupting these relationships, leading to lack of engagement with DC-CON. The concept of ‘coherence’ in NPT provided a more abstract understanding of ‘resistance’ in terms of how DC-CON may (or may not) align in meaningful ways with professional norms and expectations of professional roles and thus whether or not it is perceived to bring about a benefit that is worth pursuing.
Burden of Treatment Theory^68^	This is a theory that explains “the relationship between the demands that participating in healthcare places on patients and caregivers (their workload), and the affective, cognitive, relational and material resources that they can bring to bear on this workload (their capacity)”^48^	Several commentaries have explored how the increasing policy trend towards self-management, facilitated though DC-CON and other technologies may impact on the burden of treatment and may thus explain how service users interact with new approaches to care.^63 64^ This focus appeared to fit well with the review topic and questions. Moreover, conceptualisation of ‘capacity’ appeared to have explanatory potential for the review’s focus on equity and inclusion.	Some sources had identified that some women (who were busy with work or childcare and needed to attend multiple appointments) responded positively to DC-CON due to its ‘convenience’. This was hypothesised to potentially lead to improved motivation to engage with care and thus improved engagement with care. Considered from the point of view of Burden of Treatment Theory, ‘convenience’ could be reinterpreted in terms of reduced ‘treatment burden’ opening up new possibilities for theorising.
Candidacy Theory^45^	This is a theory of “the ways in which people’s eligibility for medical attention and intervention is jointly negotiated between individuals and health services”.^45^ It involves seven dimensions including internal processes of how people evaluate themselves to be eligible for medical care, as well as processes within the health service that enable or impede access to care: identification of candidacy, navigation of services, permeability of services, appearance at services, adjudications by professionals, offers and resistance, operating conditions.	This theory has explanatory value when considering access, equity and inclusion—all key foci of the review. Furthermore, utilisation of DC-CON potentially alters the contexts and processes through which actors construct candidacy and navigate through services.^61^ With respect to maternity, another dimension of candidacy has been identified—that of ‘understanding normality’ in the context of knowing when or when not, to seek help.^62^	Some sources identified that some women felt DC-CON enabled easier and quicker access to healthcare providers, which enabled them to quickly ask a question and put their mind at rest without feeling ‘too much of a bother’ to the system. This was initially formulated as DC-CON providing support and reassurance, leading to improved satisfaction with care. Candidacy theory helped to abstract this CMO in relation to ‘permeability of services’. DC-CON was recognised as potentially altering or complicating the ways in which women accessed care. In terms of candidacy theory, this can be conceptualised as ‘navigation of services’. In addition, discussions on who is, or is not, suitable for DC-CON were able to be theorised in relation to ‘adjudications’.
Planning and Evaluating Remote Consultation Services (PERCS) Framework^51^	PERCS^51^ is based on evidence for implementation of remote consultations in a range of healthcare settings (pre-pandemic and during-pandemic), thus capturing real-world insights of rollout and scale up. PERCS consists of seven domains. In realist terms, these can be equated with overarching ‘contexts’ (the reason for consulting, the patient, the home and family, the clinical relationship, technologies, staff, the healthcare organisation and the wider system).^51^ It includes a focus on the digital maturity of the healthcare system and digital inclusion. Taking into account the practical ethics of every day implementation, the model considers how features within these domains interact to affect key outcomes and proposes 26 implementation principles.	PERCS conceptualises DC-CON from a complex systems perspective in which all aspects, actors and contexts need to be considered in order to develop a holistic view of implementation processes, capable of taking into account the dynamic interdependencies and interactions occurring between the different parts of the system and at different levels of social structure (micro, meso, macro). As such, the model fit with the project’s focus on DC-CON within the maternity *system* (rather than looking at just one part of its implementation).^12^ In addition, by aligning the review with an existing framework, we hoped that its findings would be more transferable.	For IPT development, the PERCS model was adapted into a more streamlined maternity-focused version. The IPTs fit well into the PERCS domains. The model was further modified to illustrate realist processes (C-M-O) whereby the mechanisms were more clearly depicted and linked to a range of outcomes.

### Phase 2: evidence retrieval and synthesis: testing and refining the IPTs

#### Search strategy

Phase 2 comprised a comprehensive and systematic search for evidence related to DC-CON in maternity care in OECD countries across six bibliographic databases (Medline, Embase, PsycINFO, CINAHL, Cochrane Library and ASSIA), undertaken in July 2022 and updated in January 2023. Additionally, records already screened during phase 1 were carried forward and re-evaluated against the full review eligibility criteria. Searches to identify relevant unpublished evidence were also conducted, alongside reference list checking and citation searching (via Web of Science) of included papers. The date range for the phase 2 search was narrower (2016–2023), recognising that policy developments in UK maternity care,^21^ developments in technology (and associated user confidence/competence) and changes related to the COVID-19 pandemic had significantly altered the context of DC-CON implementation, such that prior research was deemed less likely to be directly relevant.^86^ See online supplemental file 9 for full details of the search strategies. All records were downloaded into EndNote V.X9, deduplicated and transferred to Covidence for screening.

#### Study screening, selection, appraisal and prioritisation

Study selection comprised two stages. The first stage applied detailed inclusion/exclusion criteria (see box 1), leading to a ‘longlist’ of included studies.^31^ The longlist documents were then appraised using the concepts of relevance (does the research/text address the theory under test?) and rigour (does the research/text support the conclusions drawn from it by the researchers/reviewers?).^87^ To aid transparency and consistency,^88^ these concepts were operationalised using a points-based traffic light system where studies were appraised and prioritised into nine ‘bands’.^28 89^ To represent Pawson’s idea that even methodologically weak studies can generate ‘nuggets’ of insight,^90^ higher priority was given to texts which scored well in the ‘relevance’ category compared with the ‘rigour’ category (in our review, high ‘relevance’ included having a UK focus). The team agreed an inclusion cut-off point of band 6, after which documents were excluded as they were not considered to be contributing new insights within an already large data set. The appraisal process also included a consideration of ‘richness’ to help evaluate the extent to which texts could provide in-depth explanation of *how* and *why* an intervention worked^88 89 91^ (see online supplemental file 10 for full details of the appraisal/prioritisation criteria). Systematic reviews identified within the shortlist were ‘set aside’ for analysis in phase 3.

Box 1 **Inclusion/exclusion criteria**

**Inclusion criteria**

*Participants*

Women and birthing people accessing maternity care.

Maternity care professionals and healthcare management.

*Interventions*

Studies looking at the implementation, evaluation, views and experiences of Digital Clinical Consultation (DC-CON) (as defined in the protocol).

*Comparator*

The most implicit or explicit comparator is face-to-face consultations; however, studies without a comparator will be included if they meet the other criteria.

*Outcomes*

Uptake, utilisation, engagement, satisfaction, access, equity, personalisation, quality/safety, clinical, harms, sustainable adoption, efficiency, cost

*Study designs*

Primary and secondary research of any study design, reporting empirical research, audit, evaluation and quality improvement data.

UK-focused grey literature (UK-specific reports, guidelines, policy documents, websites, conference proceedings and theses/dissertations if they are reporting primary data)

*Context/setting*

Studies within various maternity care contexts/settings and models (eg, midwife/obstetric-led care) and including different stages of the maternity care pathway (eg, antenatal, intrapartum and early postnatal period: 10–14 days).

OECD countries

*Other criteria*

Date: 2016–present. The initial focus in phase 2 is on texts published from 2016–onwards, but studies from 2010 will be considered (in phase 3) to address gaps in the evidence base

Studies about maternity care during COVID-19 will be included for full-text screening on the assumption that DC-CON is likely to have occurred, even if this is not explicitly clear from title and abstract screening

**Exclusion criteria**

Studies not in English; studies where the full text is unavailable, protocols; non-UK focused opinion pieces/editorials

Studies not explicitly focused on service user-healthcare provider consultations, for example, online antenatal classes

Studies not explicitly focused on maternity care, but other areas of reproductive health, for example, abortion, fertility or contraceptive care

Studies focused on services/interventions provided by non-maternity care professionals/providers (eg, drug and alcohol services, specialist mental health services, stopping smoking services, weight management services). We recognise that there may be regional and national variation in the delivery and commissioning of maternity supportive services and therefore such studies will be discussed on a case-by-case basis within the research team and assessed for inclusion in consideration of the role and involvement of the maternity care professional. As a general rule for overseas studies, these will be included if they describe a service which, in the UK, would typically be provided by maternity professionals within commissioned maternity services.

#### Data extraction, analysis and synthesis

Key characteristics of included evidence sources were extracted into an Excel spreadsheet. PDFs of all included sources were imported into NVivo.^92^ Coding of data was initially structured according to the 13 IPTs (supplemented by analytical memos) with new nodes created for data that did not ‘fit’.^33 93^ Analysis was an iterative process, drawing on the PERCS conceptual framework^51^ and mid-range theories to aid in a critical and ongoing process of abductive and retroductive theorising.^85 93^ This phase also involved further meetings of the knowledge user groups in which clinical scenarios incorporating different IPTs were presented for ‘sense-checking’ and challenge. Collectively, these processes led to a reconfiguration of the 13 IPTs into 16 IPTs.

### Phase 3: validation and strengthening of PTs and generation of recommendations

This phase included several steps to sense-check and further refine the IPTs. The systematic reviews identified in the phase 2 search were analysed to confirm, refute or refine the IPTs. Each IPT was also evaluated in terms of the quality, quantity, consistency and applicability of the underpinning evidence. Most IPTs were deemed to be well supported by their underpinning evidence and none were categorised as weak. However, a few IPTs—primarily those relating to safety and equity outcomes—were relatively less well supported with contextually relevant data which provided clear causal insights. To address this, with help from community organisations, three additional consultations (one online, two in-person) were undertaken with women (who were pregnant or had recently had a baby) from minoritised and underserved communities (n=22). These consultations were designed to explore the IPT’s credibility and completeness and helped the team to recognise more clearly the ways in which the PTs operated in different contexts. For example, the consultations made it clear that navigating an unfamiliar system (PT 4.1) was as much of a potential challenge for appropriate DC-CON use as language barriers (PT 4.2). These insights had already been identified in the literature, but were given more depth and application to the UK context through the additional consultations. The team also undertook an additional focused search (in March 2023) to find evidence specifically related to DC-CON (expanded to include non-maternity settings), equity and safety.^2930 42 91 94^,^97^ This search was applied across four bibliographic databases (Embase, Medline, CINAHL, PsycINFO), supplemented by well-established CLUSTER search processes.^91^ The latter integrates a range of methods to build up ‘clusters’ of evidence linked to specific PTs.^43 91 94 95 98^ These included citation and reference list searching, keyword searching of the existing EndNote library, Google Scholar searches, key website searching, snowball searching from key author papers and key author publication alerts (see online supplemental file 11 for phase 3 search strategies). As in phase 1, study selection and appraisal were highly purposive, aimed only at finding papers that could offer key additional insights.

The insights and evidence from phase 3 contributed to further iterative theorising and reconfiguration of the IPTs into a final set of 15 PTs organised within 5 domains. These were sense-checked and agreed in a third round of knowledge user group consultations and in a 1-day workshop which distilled the PTs into key implementation principles. Both activities generated key recommendations for service development, policy/systems development and research.

## Results

### Search, screening and appraisal results

**Figure 1:**
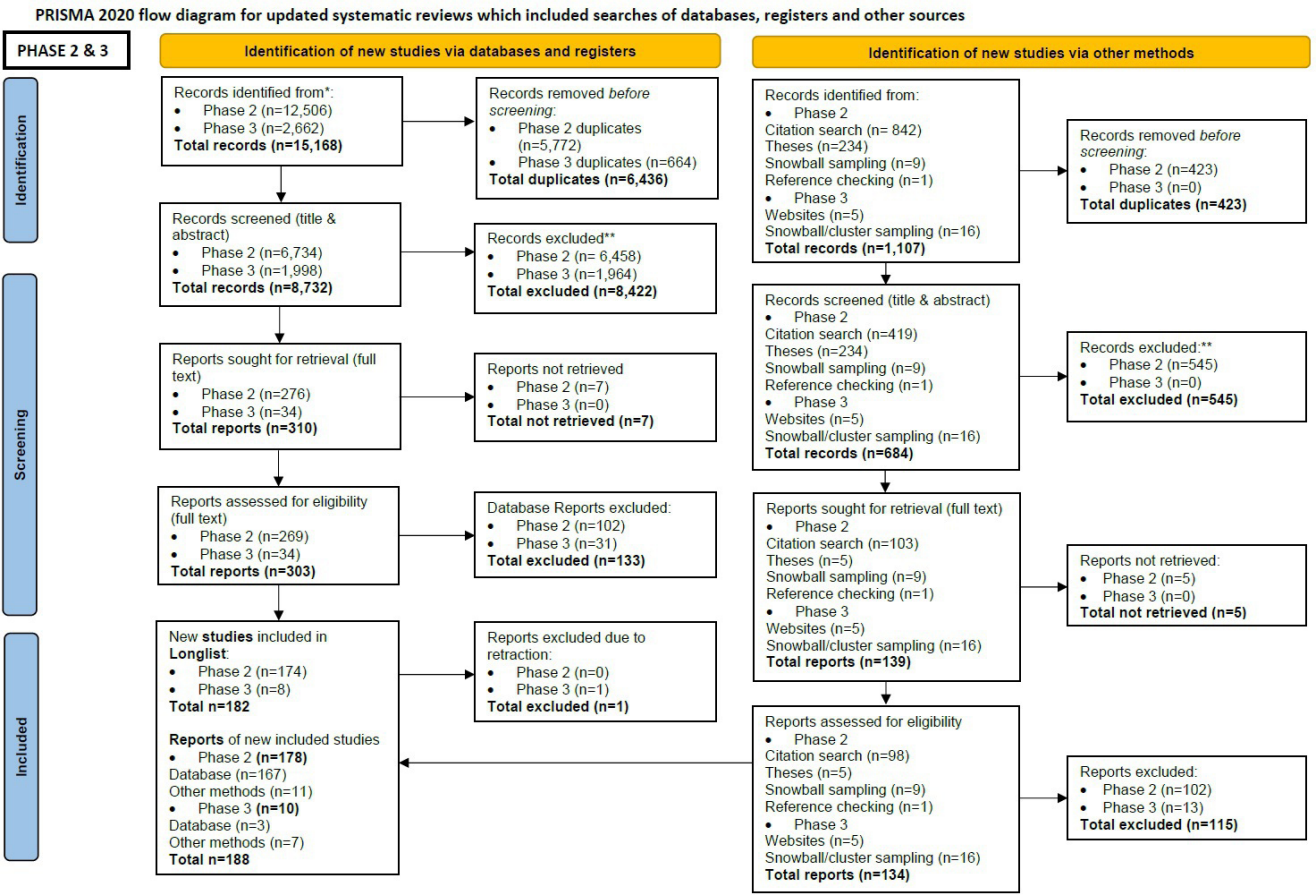
Preferred Reporting Items for Systematic Reviews and Meta-Analyses 2020 flow diagram (phase 2 and phase 3 searches).

Following deduplication, the comprehensive and focused searches in phases 2 and 3 resulted in 9416 records for title and abstract screening. Full-text screening was undertaken for 437 records, following which 189 reports were included within the review’s ‘longlist’—see figure 1 (Preferred Reporting Items for Systematic Reviews and Meta-Analyses flow chart).^99^ After the appraisal and prioritisation process (and 1 retraction), 93 evidence sources were ‘shortlisted’ to be included in the synthesis. These included empirical papers (n=77 11,1346 59 75 78 80 81 100), reviews (n=11^35 7 9 168^,^174^) and unpublished reports (n=5 175,179).

### Characteristics of included studies

See online supplemental file 12 for a detailed table of the characteristics of included evidence sources (n=93). The evidence sources that were not prioritised but were included in the longlist (n=95) are listed in online supplemental file 13.

#### Primary research

The 77 empirical papers included 1 RCT (2 publications),^108 164^ 1 quasi-experimental study (2 publications)^146 147^ and 1 interrupted time-series analysis.^11^ The remaining papers were observational studies (n=10), quality improvement reports (n=1), mixed methods (n=19), qualitative (n=22) and cross-sectional surveys (n=20). Twenty-five papers had a UK focus.^1213 80 81 101 104^,^107 127 139 140 148 150 151 153 154 156^ The majority of other studies were undertaken in the USA (n=35), with some from Europe, Australia and New Zealand. Approximately one-third of papers (n=25) were undertaken prepandemic^4659 80 102^,^104 108 110 112 114^; the rest reported on DC-CON use during, and in the aftermath of, the pandemic.

In terms of DC-CON modality, in 7 papers, DC-CON was used in combination with remote monitoring systems,^59 108 122 146 147 164 166^ 16 papers explored the use of video calls only,^1146 78 80 104^,^106 112 119 133 134 136 138 143 156 159^ and 13 papers described phone calls only.^102107 110 113^,^117 128 148 150 151 155^ The remaining papers referred to both telephone and video options, often collapsing these into a generic overarching term such as ‘virtual’ or ‘remote’ care and not distinguishing any further. The majority of papers provided only limited detail on the infrastructure, systems, governance and other processes underpinning DC-CON. Approximately half (n=38) of the papers focused on the antenatal period; 4 papers related to the postnatal period^78 112 123 128^; and 31 papers focused on both aspects (or it was unclear). Eight papers focused specifically on triage.^4680 102 105 106 114^,^116^

DC-CON was being used to support specific medical or psychosocial issues or within different clinical contexts, but the majority of papers did not specify this clearly. Eight papers focused on care for women with pre-existing diabetes or gestational diabetes^59124 131 149 154^,^156 162^; one paper was focused on outpatient care for women with diagnosed COVID-19^132^; three papers related to maternal–foetal medicine^133 137 159^; two papers focused on women with hypertensive disorders^128 166^; five papers focused on high-risk pregnancies (variously defined)^78 81 109 126 134^; three papers included high-risk and low-risk pregnancy contexts^136 145 180^; two papers included a focus on mental health^125 140^; two papers included a focus on safe-guarding and domestic violence^119 125^ and two papers focused specifically on models of care for women with complex social risk factors.^150 151^

Clinical and safety-related outcomes were reported in 12 studies^11 108 109 113 117 132 134 146 150 155 159 162^; only 4 studies reported on cost (to users or the health system).^130 155 162 164^ Seventeen publications reported outcomes related to healthcare utilisation and efficiency.^81108^,^110 113 126 128 132 137 144 145 150 151 155 162 164^ Fifteen papers focused explicitly on health equity in terms of their objectives or analytical approach.^12 13 78 109 117 123 128 131 137 144 150 151 153 161 162^ The majority of papers (n=66) provided information related to satisfaction, acceptability, views, experiences, barriers or facilitators.^1213 46 59 75 78 80 81 100^,^108 110^

In terms of study population, 17 papers focused on healthcare professionals, 45 papers focused on women and 15 papers had a mixed sample. The most commonly reported sample characteristics were age, sex and educational level. Data on race and/or ethnicity were reported in only half the papers (n=39).^1213 75 78 107^,^109 111^ Seven papers reported that their sample included women who were migrants or who did not speak English.^78 123 131 133 136 150 151^ Three papers mentioned participants with a hearing-related disability.^81 101 127^ Two papers had a specific focus on the experience of young mothers/parents.^139 140^ One paper focused on veterans,^110^ and three had a specific focus on low-income women.^123 150 151^ Seven papers were focused particularly on rural areas^104 112 117 134 141 149 159^ (the other papers were a mix of urban/rural, urban or did not specify). Within many papers, there was limited commentary on how representative the sample was of the relevant populations and, therefore, it was often unclear whose voices were being heard or being missed.

#### Reviews and other sources

The 11 reviews^35 7 9 168^,^174^ included foci on safety-netting,^173^ continuity of care,^171^ breastfeeding,^170^ telephone triage,^169^ antenatal care^5 7 174^ and ‘generic’.^3 9 168 172^ Only three of the reviews included equity objectives,^3 9 171^ with two noting that it was not possible to undertake relevant analyses due to a lack of sufficiently detailed/disaggregated equity-related data in the primary studies.^3 9^ The five reports were all UK based, focusing on the clinical, safety and equity-related impacts of the COVID-19 pandemic on maternal/neonatal outcomes.^175^,^179^

### Findings: key PTs

**Figure 2:**
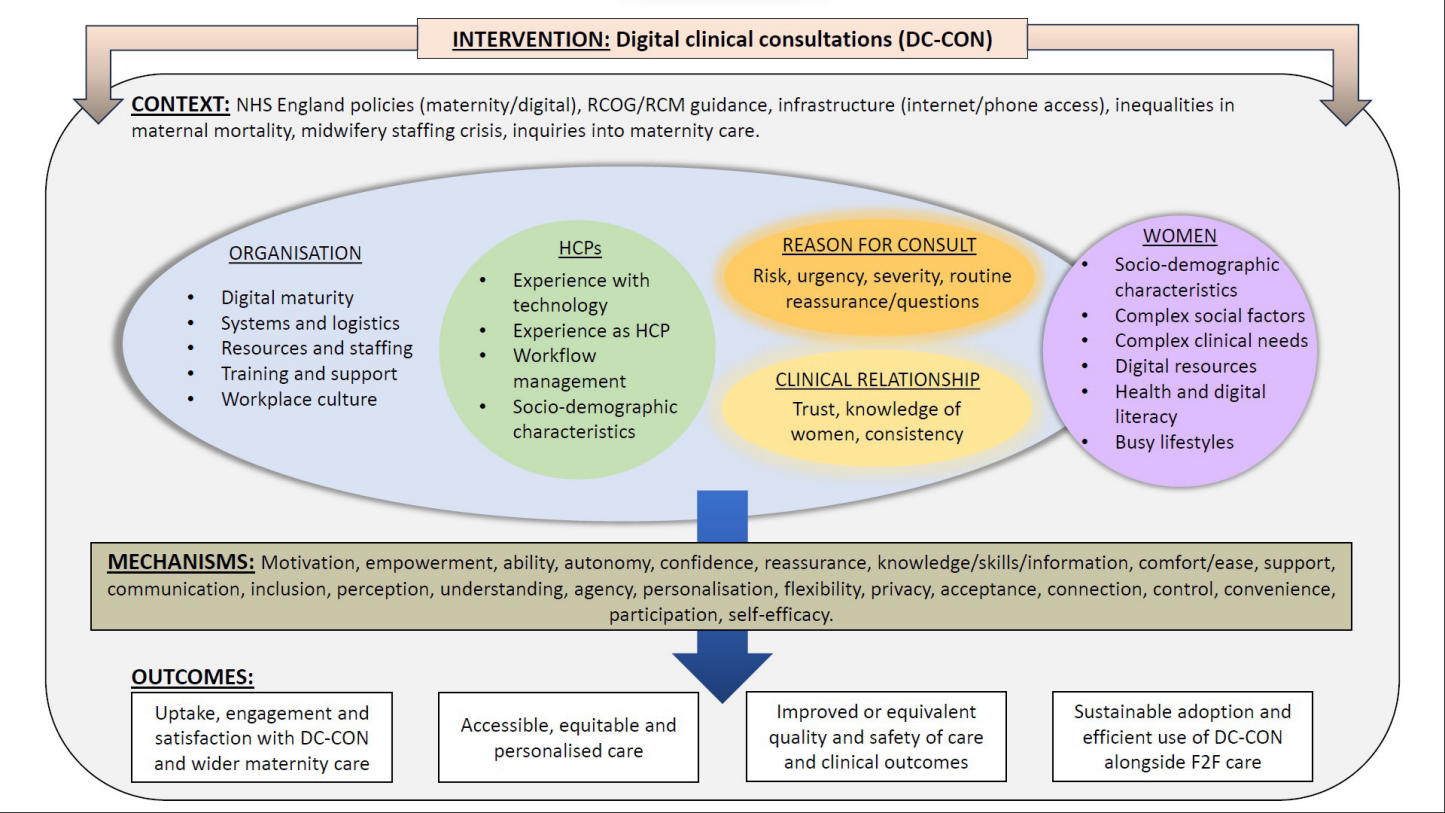
Programme theory (PT) conceptual model. HCP, healthcare professional; NHS, National Health Service.

The synthesis developed 15 PTs expressed as C-M-O configurations, organised within 5 domains (see box 2). Online supplemental file 14 provides detail of the evidence underpinning each PT, the key contexts to which they relate, supporting quotes and additional insights from knowledge users. The key contexts and mechanisms underpinning DC-CON implementation are represented visually in figure 2 which is a maternity-focused adaptation of the PERCS model.^51^ The model provides a way of considering DC-CON implementation across the whole maternity system. It identifies five interlinked contextual dimensions (the organisation, HCPs, reason for consulting, the clinical relationship and women) that need to be considered for DC-CON implementation. These, in turn, are situated within a wider national health system context. The PTs show that different potential mechanisms are activated, depending on varying configurations across these five contextual dimensions, thus leading to different potential outcomes. The PTs incorporate insights from Candidacy,^45^ Normalisation Process^66^ and Burden of Treatment^63^ theories; however, the latter was renamed ‘Burden of Care’ (BoC) as this terminology better represented the maternity context.

Box 2 **Programme theories (PTs)**

**Programme theory domain 1: infrastructure and resources**

**PT 1.1. Developing infrastructure:**

If organisations take adequate time to provide a digital infrastructure (including reliable equipment, software, internet), developed with staff input to make it user-friendly [**C**], healthcare providers will feel confident [**M**] that digital consultations [**I**] are a tool that can ‘fit’ into existing work practices [**C**]. Hence, staff will feel motivated [**M**] to embed it into their practice [**O**].

**PT 1.2. Establishing clinical systems and pathways:**

If digital consultations [**I**] are supported by administrative systems and integrated electronic patient record systems that can operate across contexts [**C**], it will improve the ability of staff to access information, work in multidisciplinary teams and coordinate care across the pathway [**M**]. When systems work well, digital consultations are perceived by staff to improve existing workflows—increasing convenience, efficiency and reducing workload [**O**]—for organisations, staff and service users—as well as maintaining safety [**O**].

**PT 1.3. Appropriate staffing models and conditions:**

If staffing models for digital consultations include dedicated teams in private spaces with the capacity to provide continuity of carer [**C**], this type of working environment can enhance staff and women’s sense of privacy and comfort [**M**] facilitating the communication of concerns and treatment [**O**]. This helps women and staff feel confident and motivated [**M**] to use digital consultations (and sustain their use) [**O**].

**Programme theory domain 2: training and support for staff**

**PT 2.1. Providing staff training and ongoing support:**

If National Health Service and professional organisations provide a supportive and enabling workplace culture for digital clinical consultations (including sufficient training, protected time for training, appropriate workspaces and ongoing access to clinical, technical and administrative support) [**C**], staff will gain relevant knowledge/skills [**M**] and will feel more motivated, supported and confident [**M**], leading to appropriate and sustained uptake of digital consultations [**O**].

**PT 2.2. Ensuring staff motivation and ‘buy-in’:**

If staff are informed about the potential benefits of DC-CON [**C**], to both HCPs and women, it can promote staff ‘buy-in’. In particular, if staff perceive [**M**] that women accept, are benefitting from, and satisfied [**O**] with, digital consultations they will be motivated [**M**] to use it (buy into and sustain its use) [**O**] and gain job satisfaction from using it [**O**].

**PT 2.3. Providing clinical protocols on consultation mode:**

If digital consultations are guided by clear clinical protocols [**C**], staff can feel supported [**M**] in deciding what type of consultation is appropriate to meet women’s varied needs and preferences. When digital consultations are further enhanced with the use of at-home monitoring [**C**], it can provide additional reassurance to professionals and women [**M**] of the quality and safety of DC-CON [**O**]. Combined, this can increase staff ability, acceptance and confidence in monitoring and treating women at a distance [**M**], leading to optimal clinical/safety outcomes [**O**].

**Programme theory domain 3: personalisation and flexibility for women**

**PT 3.1. Supporting choice and personalisation of care:**

If digital consultations are clearly presented to women as a choice within a hybrid model of care, [**C**] then women will be reassured [**M**] about the option to still have face-to-face appointments when necessary. Furthermore, *i*f the use of digital consultations [**I**] is personalised [**M**] to women’s needs, preferences and life circumstances [**C**], women can feel a sense of safety and empowerment [**M**]. This can help digital consultations to be accepted as a valuable addition to traditional maternity care [**O**].

**PT 3.2. Managing the burden of care:**

If digital consultations are easy to use and fit flexibly [**M**] with women’s preferences, life circumstances and clinical needs [**C**], it gives them more control over the time, money and effort they have to engage with care [**M**]. This can be a relief and for some women will make it less burdensome [**M**] for them to engage with services [**O**]. It can also make it easier [**M**] for women to access services/specialists in a wider geographical area, potentially improving clinical outcomes [**O**].

**Programme theory domain 4: women’s access and inclusion**

**PT 4.1. Supporting women’s knowledge and navigation of care:**

When comprehensive information on digital consultations is provided to women in an easy to understand, accessible format and in a variety of languages, it can facilitate health and digital literacy [**C**]. If women are made aware of the different types of consultations available to them when they first engage with the maternity services [**C**], they can be empowered [**M**] to make informed choices about the mode of care they receive [**M**]. This will improve the potential for personalisation [**M**] of care delivery, enable access [**O**] and help women to play an active role in their maternity care [**O**].

**PT 4.2. Ensuring inclusion and equity:**

While there can be benefits to using digital clinical consultations [**I**], for women who face language or other communication barriers [**C**], digital clinical consultations [**I**] can present a challenge to the equitable access of care [**O**]. Experiencing communication barriers can create frustration or anxiety, a lack of motivation or sense of entitlement [**M**] to engage with care [**O**]. This can lead to particular groups of women receiving less or inappropriate care relative to their needs [**O**], important issues being missed and suboptimal clinical outcomes [**O**].

**PT 4.3. Considering access to digital resources:**

If women do not have access to digital devices, a reliable internet connection or telephone signal [**C**], it may lead to feelings of disempowerment, frustration and loneliness [**M**] as women will struggle to engage with digital clinical consultations [**O**]. This is likely to disproportionately affect already vulnerable women living in poverty or unstable circumstances [**C**], exacerbating health inequalities through digital exclusion [**O**].

**Programme theory domain 5: quality care through relationship-focused connections**

**PT 5.1. Considering safety and managing risk:**

Digital clinical consultations [**I**] provide staff with additional methods with which to communicate with women [**C**]. When healthcare professionals are matching the mode of consultation to the reason for consultation [**C**], understanding [**M**] women’s physical, psychological or social circumstances and risks [**C**] can help staff to personalise care and manage uncertainty [**M**]. This can lead to equivalent clinical outcomes [**O**], and safety assurances [**O**].

**PT 5.2. Managing relationships and building rapport:**

If digital consultations are used in place of face-to-face care, it can affect the women–healthcare provider relationship [**C**]. Since video calls enable the conveyance of non-verbal cues [**M**], they can be more beneficial in relationship building than telephone calls [**O**]. If a relationship of trust has already been established and there is sufficient time for the consultation [**C**], then staff and women can communicate easily and openly [**M**], improving women’s disclosure of sensitive information and feelings of reassurance [**M**]. For both routine and complex care via digital consultations, continuity of carer can lead to greater satisfaction for women and professionals and is perceived to support optimal clinical outcomes [**O**].

**PT 5.3. Supporting women’s empowerment and familial involvement:**

If women have the ability to use digital consultations [**C**], it can make it easier to facilitate women’s active participation [**M**] in partnership with their healthcare provider, especially if remote monitoring is used [**C**]. The flexibility and convenience of digital consultations [**C**] can also help to include women’s partners/families [**M**] in their care. This can empower, motivate and give women a sense of control over their health and care, [**M**] improving access and enhancing engagement with services [**O**].

**PT 5.4. Offering connection and support:**

If digital consultations can provide additional and/or convenient opportunities for women to connect with services and staff [**C**], it can support women’s sense of safety, reassurance and empowerment [**M**]. These benefits may be enhanced by a pre-existing healthcare provider–woman relationship, good communication and sufficient time for the consultation [**C**]. This leads to increased self-efficacy and motivation [**M**] contributing to satisfaction, engagement and access [**O**].

#### Domain 1. Infrastructure and resources

The organisational infrastructure and resources provided to support DC-CON have a major impact on how it is practically implemented and how staff respond; hence, this domain links across the ‘organisational’ and ‘HCP’ contexts in figure 2.

Access to digital resources, connectivity onsite and offsite, as well as DC-CON software, form key aspects of an organisation’s digital readiness (**PT 1.1**).^12 121 125 149 167 168^ However, it takes time for organisations to develop the infrastructure necessary to achieve potential efficiency gains of DC-CON and implementing this service too rapidly (as happened during COVID-19) can create frustration for women and HCPs.^110^ Establishing clear clinical systems and pathways was highlighted as important to facilitate connection and coordination (**PT 1.2**). Key areas include queuing systems, call-back procedures and, most importantly, interoperability between DC-CON software and patient records. Where these worked well, they were found to increase permeability to services and reduce the BoC for women,^12 13 105 136 148 167 168 175 177^ as well as improve convenience, efficiency and workload for staff.^59 131 148^ However, when systems worked poorly, DC-CON could create hidden work for staff, double handling and wasted resources.^12 13 81 110 149^

Another central aspect of planning and resourcing a DC-CON service is appropriate staffing models and conditions (**PT 1.3**). This included recognising DC-CON as an important and distinct activity requiring dedicated and trained staff with protected time and appropriate (private and quiet) workspaces.^12 80 102 105 106 177^ A suitable working environment was also considered important to address staff concerns that digital care can lead to a loss of shared spaces and may negatively affect teamworking and communication.^12 131 136^ However, DC-CON was also recognised for its potential to facilitate multidisciplinary working and care coordination (especially when relevant specialists were in different locations), provided that systems could support this (**PT 1.2**).^12 104 121 156^

With respect to the UK, the review highlighted significant variation in DC-CON ‘readiness’ between and within National Health Service (NHS) Trusts (**PT 1.1**). This caused staff to doubt whether video calls in particular were technologically feasible,^12 81 104^ with telephone calls seen as a more reliable ‘back-up’ option.^80 132^ Telephones were the main modality in triage services which can help to manage care effectively within the maternity pathway (preventing spill over into other services, eg, GP surgeries or emergency departments) (**PT 1.2**),^80 151^ thus reducing unnecessary visits to hospital and allowing women to receive care at home.^80 100 105 125^ As such, the need for effective telephone infrastructure and systems was apparent. When video was used, mainstream applications such as WhatsApp were perceived as easier to access and more user-friendly than NHS software (**PT 1.1**).^105 106 121 129 152^

#### Domain 2. Training and support for staff

This domain highlights the importance of providing support for staff to use safe and high-quality DC-CON. In figure 2, domain 2 links across the ‘organisational’ and ‘HCP’ contexts, as well as the ‘reason for consulting’. It draws particularly on NPT to explore the work that people do while implementing DC-CON.

There was a strong desire from staff for specific DC-CON training (including technology/systems, communication and risk assessment skills) which was linked to improved staff satisfaction, efficiency, safety and quality of care (**PT 2.1**).^5 9 46 80 102 105 106 110 114 116 121 129 132 152 169 177^ Several studies linked the desire and need for training to staff age and clinical experience, suggesting that support may need to be tailored (**PT 2.2**). For example, younger staff may have more positive attitudes towards DC-CON and more confidence using video technology.^12 122 129 163^ Staff also expressed a need for ongoing IT and administrative support to better manage the workload associated with DC-CON and improve workflows (**PT 2.1**).^75 114^ However, the literature highlighted that a lack of training and guidance was commonplace.^102 148^

A key motivating factor for staff acceptance was knowing whether women accepted, wanted and/or were satisfied with digital consultations (**PT 2.2**).^12 80 104 105 110 120 148 152^ In addition, staff confidence in, and acceptance of, DC-CON was linked to the need for clinical support and governance systems (including clinical protocols, preclinic vetting systems, safeguarding guidance, structured approaches to conducting and recording DC-CONs, safety-netting guidance and quality-assurance systems) to maintain safety and quality of care (**PT 2.3**).^9 80 81 105 116 132 145 149 165 169 173 175 177^ However, staff also wanted to be able to exercise professional autonomy in choosing or adapting the consultation mode according to a woman’s needs, but also according to their own preferences, work context and skills (**PT 2.2**).^12 13 80^ For example, staff perceived that DC-CON could improve their efficiency at work since it would offer them greater convenience (eg, less travelling) and flexibility to manage their workload.^75 122 129 136 140 152 167 168^ Nonetheless, while these factors could improve staff acceptance of DC-CON, the potential for DC-CON to offer tangible efficiency gains was dependent on whether or not it was operating under ‘optimal conditions’.^12 103^

#### Domain 3. Personalisation and flexibility for women

This domain relates to person-centred care and the ability to adapt consultations to meet women’s needs and preferences and to fit with their life circumstances. In figure 2, this domain includes interactions across multiple contexts (‘HCP,’ ‘women’, the ‘clinical relationship’ and ‘reason for consulting’).

The literature strongly highlighted the importance of women being able to choose and personalise their consultation modality, with studies emphasising that preferences and experiences varied greatly according to individual dispositions, needs and circumstances (**PT 3.1**).^12 106 111 130 148 154 158 174^ The emphasis on choice was perhaps particularly prominent as a consequence of experiences during the pandemic where women (and staff) had little control over the use of DC-CON and worried about a lack of physical examinations.^12 101 107 131 154 155 157 158 160 172^ Interestingly, some studies reported high levels of satisfaction with DC-CON during the pandemic but *satisfaction* (within an exceptional situation) cannot necessarily be equated with a *preference* for DC-CON in ‘normal’ times.^135 167^ Indeed, some studies found that women do not perceive DC-CON to be ‘real’ appointments and find in-person care more reassuring and productive.^111 123 131^ Online supplemental file 14 provides detail on specific contexts (including medically complex or socially vulnerable women), in which choice and personalisation are highlighted as especially important.^13 100 101 107 124 127 146 153 159^

Tailoring care to women’s needs (eg, medical, social and psychological) and preferences (including appointment times and modality) could help to reduce the BoC for some women, as well as facilitate the involvement of partners and family members in care (**PT 3.2**). This can improve women’s satisfaction, engagement in and sense of control over, their care, as well as benefit the women–HCP relationship.^57 9 12 13 78 80 81 104 108 111 117 118 123^,^125 130 131 134 136 138 143 145 148 154 155 158 160 161 163 165 166 172 174^ Some HCPs perceived that the convenience of DC-CON could help to reduce ‘did not attend’ rates.^9 113 131 148^ However, while DC-CON can relieve the BoC in some respects, it can also create new context-specific burdens such as the logistics of downloading DC-CON software, finding a private space to talk and juggling DC-CON alongside concurrent responsibilities (**PT 3.2**).^141^

#### Domain 4. Women’s access and inclusion

This domain considers equity, diversity and inclusion in DC-CON implementation, drawing on Candidacy theory in particular to explore how DC-CON may impede or facilitate access to services and quality of care.^45^ In figure 2, domain 4 particularly illuminates features associated with the ‘women’s’ context. Much of the literature in this domain reported staff views on how DC-CONs might affect vulnerable groups, with less evidence from women themselves. As such, additional stakeholder consultations with women from under-represented groups were conducted to validate the PTs in this domain.

Many studies suggested that women wanted help and reassurance using DC-CONs (**PT 4.1**), including: what DC-CON was, why it was being used, what would happen during the call, who would make the call, the schedule of face-to-face versus digital appointments, technical support (particularly for video calls), training on remote monitoring equipment (if used), a practise call and a contingency plan if the technology fails.^746 80 104^,^106 111 112 121 123 136 138 145 154 158 167 168 174^ Women wanted this information to be clear, consistent and provided in advance to limit the BoC and, in turn, improve satisfaction.^5 46 112 145 154 158 174^ In some situations, DC-CON-related information was perceived as confusing, particularly when there were multiple service access points (eg, multiple phone numbers). This could impede the navigation and permeability of services, leaving women unsure or unaware of how and who to contact for help when needed, and potentially compromising safety.^115 158 177^

Some studies highlighted an assumption that, as pregnant women were part of a ‘tech-savvy’ generation, they would not struggle to access digital maternity care (**PT 4.1**).^81 104^ Other studies challenged this assumption however, with women and staff noting several groups who may struggle in navigating DC-CON, including those with mental health problems, lacking technical skills or facing communication barriers.^129 138 140^

Many evidence sources highlighted the need to acknowledge potential communication barriers (eg, language, disability or neurodiversity) in DC-CON to ensure equitable access (eg, by providing instructions and software in appropriate formats) (**PT 4.2**).^12 172^ UK sources noted that digital NHS interpretation services are not always available, easily used or of sufficient quality, causing women to seek in-person care instead via GP surgeries or emergency departments.^12 81 106 175 176^ Problems with professional interpreters can cause family members to provide translation, even though this is against NHS guidance.^177^ Three UK studies (during COVID-19) described women with hearing disabilities struggling to engage in video consultations without a sign language interpreter.^81 101 127^ Difficulties with interpretation services were also reported in other countries,^123 131 137^ with one review of perinatal mental health, suggesting that women strongly valued being able to express themselves and receive assistance in their own language.^174^ The evidence also indicated that women with language barriers were less likely to want DC-CON and less likely to be offered DC-CON by staff (exemplifying the adjudications being made in considering suitability for DC-CON).^109 121 131 137 178^ While language barriers could be difficult to mediate digitally, video calls were felt to aid communication (compared with phone calls) by enabling visual assessment, interpretation of non-verbal cues and using drawings/diagrams.^46 80 105 123^

Another equity consideration for DC-CONs relates to access to digital resources (eg, mobile data, internet, equipment) (**PT 4.3**). Where consistent access to relevant resources could not be assured, DC-CONs could be stressful or impossible for certain women, reducing permeability to services and amplifying inequalities.^9 12 13 78 80 109 121 123 124 136 139 145 149 163 168^ Although audio-only communication had recognised limitations (particularly regarding language), telephone consultations were typically considered cheaper, more accessible and reliable than video consultations.^7 12 106 123 131 157 174^ Studies from Australia and the USA noted that women may incur additional costs to purchase and run remote monitoring equipment.^75 131 149^ In some situations however (eg, for women in rural and remote areas), evidence suggests that DC-CON can increase access to care (provided there is adequate internet/phone service).^9 141^

#### Domain 5: Quality care through relationship-focused connections

This domain explores issues of patient safety and clinical outcomes in DC-CON. In addition to other sources, evidence in this domain includes recent reports from MBBRACE-UK and the Healthcare Safety Investigation Branch who independently examine cases of patient safety in the UK NHS to improve systems and processes. In figure 2, this domain shows interactions across multiple contexts, but particularly highlights mechanisms related to the ‘clinical relationship’.

A common theme expressed in many studies was a concern about DC-CON safety and quality. However, the evidence consistently suggested that clinical outcomes for women who had experienced digital maternity care were comparable to in-person care, while potentially improving access, satisfaction and (in some cases) health disparities (**PT 5.1**).^3 7 9 11 108 113 134 146 162 174^ Indeed, in some studies undertaken during COVID-19, staff reported positive attitudes towards safety and care quality and were optimistic about continuing DC-CON post pandemic.^133 136 142 143 148 165^

The evidence suggested a link between quality and safety and the ability of women and staff to personalise the consultation modality and to manage key contextual factors that may create risks in DC-CON implementation. Key contextual factors relate to the reason for consultation (eg, transactional vs complex/sensitive enquiries, the need for physical examination or perceived risk), and women’s circumstances (eg, proximity to care facilities, communication barriers, psychosocial concerns) (**PT 5.1**).^12 13 75 81 111 119 121 125 129 131 132 137 138 140 145 149 157 158 163 172 178 179^ Key mechanisms for managing these contextual factors lie within the relationship between women and providers (**PT 5.2**). Maternity care was described by some women and midwives as a uniquely personal experience, which necessitated personal contact.^13 118 121 129 172^ Where DC-CON was provided by a known HCP, it could promote relationship building, avoid fragmentation of care and repetition of information, while improving women’s feelings of comfort, reassurance and ability to disclose sensitive information (limiting the BoC).^46 105 106 112 117 124 145 148 149 174^ When there was no consistency in the HCP providing DC-CON, women’s trust in professionals could be diminished, leading to reduced engagement and potential safety concerns.^12 131^

Building an effective clinical relationship involves rapport, often linked to the ability to visualise, empathise and make sense of non-verbal cues (**PT 5.2**). Where this proved challenging (eg, during phone calls), HCPs worried about missing something important, for example, intimate partner violence.^125^ Similarly, women reported that seeing their HCP’s body language, particularly when receiving difficult news, could provide a better understanding of the situation.^7 111 123 138^ More positive experiences of DC-CON were reported when women perceived that the HCP listened, was friendly, empathetic, reassuring, made eye-contact, explained clearly, invited questions and had sufficient time.^111 115 138 147 148 154 158^ These features were more prominent in video or face-to-face care, but not exclusively. Additionally, establishing a relationship over DC-CON (eg, during triage) could facilitate a better connection when the woman and provider later met in-person.^80 103^ Regardless of the women–provider relationship, an important (but currently not well evidenced) finding relates to the need to provide ‘safety-netting’ advice during DC-CONs to manage risk (see^173^) (**PT 5.1**).

In situations where DC-CON formed an integral part of a care process (eg, at-home remote monitoring), some women were apprehensive about having additional responsibility and perceived that assessments should be conducted by medical professionals (particularly during COVID-19) (**PT 5.3**).^118 123 124 139 157^ However, studies indicate that educating woman on the equipment and process of remote monitoring–ideally in-person^131^–could help to alleviate concerns and improve experiences.^166 168^ Other women experienced remote monitoring as an efficient and empowering way of undergoing regular checks, developing their self-management skills and sense of responsibility for the health of themselves and the baby, potentially improving satisfaction and lowering stress.^7 9 12 103 111 122 124 131 142 145 166 174^ In addition, the convenience and flexibility of DC-CON were found to improve partner and familial involvement in maternity care.^5 78 105 106 134 140 154 168 174 175^

While the flexibility of DC-CON could be beneficial, communication around symptoms and safety-netting was highlighted as particularly challenging and complex processes (**PT 5.4**),^46 78 175^ leading to frustration or anxiety during a call or to concerns about safety. Communication could be particularly problematic if calls took place when women were not prepared (eg, at an unplanned time) and were in a public place or multitasking.^81 131^

The evidence suggested that easy, flexible (potentially out-of-hours) access to care via DC-CON can improve women’s sense of eligibility for care (eg, by perceiving that telephone or video calls place less of a burden on busy staff or services^78 175^) and therefore provide women with an important source of connection and support as and when they need it (**PT 5.4**).^12 105 106 112 115 117 154 155 166^ The perception of eligibility to care is particularly increased where contact is with a known HCP as in midwifery continuity of carer models.^78 150 151^ In this context, DC-CON may improve access/engagement in care (particularly when used with vulnerable women).^103 117 151^

Overall, the PTs suggest that the nature and quality of the woman–provider relationship can help to optimise safety and clinical outcomes, as well as support women to develop self-management skills and recognise themselves as candidates for care.

## Discussion

This review aimed to illuminate ‘*what works, for whom, and in what contexts’* in relation to DC-CON in maternity care. Overall, the PTs propose that DC-CON use can be safe and acceptable to stakeholders if implementation can ensure equity of access, personalisation and professional autonomy. Key mechanisms that support implementation for women and families include a sense of control and empowerment, personalisation, knowledge, motivation, ease of use, reassurance, sense of connection, communication and participation. Underlying contextual conditions for women include access to material, social and digital resources, capacity and a flexible system that enables information sharing and can adapt to women’s needs and preferences. Key implementation mechanisms for staff include convenience, motivation, knowledge/skills, perceived support, confidence, professional autonomy, communication and the ability to personalise care. Underlying contextual conditions for staff include provision of clinical guidance, resources, infrastructure and integration with record systems as part of a workplace culture that provides support and training.

The synthesis demonstrates that there is no one-size fits all for DC-CON. However, taking the PTs all together, it is possible to identify key implementation principles which can guide future practice and research. We name these the CORE principles (see table 2) and elaborate these below in relation to relevant literature and mid-range theory.

**Table 2:** CORE principles

CORE principles	Link to programme theory domains
**C**—Creating the right environment, infrastructure and support for staff	1 and 2 (these are merged to reflect the interaction between HCPs and their organisational context)
**O**—Optimising consultations to be responsive, flexible and personalised to different needs and preferences	3
**R**—Recognising the importance of access and inclusion	4
**E**—Enabling quality and safety through relationship-focused connections	5

HCPhealthcare professional

### C—creating the right environment, infrastructure and support for staff

A prerequisite for DC-CON is a reliable digital infrastructure that staff can integrate smoothly into existing workflows and practices and which provide interoperability across systems.^83181^,^183^ Studies from the UK NHS, across different healthcare settings, suggest that this remains an enduring challenge, with stakeholders expressing particular frustration at (1) a lack of integration with patient records and (2) the need to use bespoke Apps and virtual platforms which create additional obstacles and do not easily ‘fit’ with systems that are already widely used by staff and service users.^69 83 184 185^ This is particularly the case for video technology whose uptake has been slow and patchy across different specialities, leading to a default tendency to use the telephone.^50 186 187^ These concerns are strongly reflected in the current review findings.^13 81^ However, it is important to note that a significant portion of evidence sources in the review did not clearly specify the DC-CON modality (referring generically to ‘virtual’ or ‘remote’ care) or did not provide full details on precisely how it was being used within a service. This limits our ability to draw clear conclusions in relation to infrastructure, staffing and organisational or IT systems. To address these limitations, future research should consider using in-depth case study designs and should report the details of DC-CON using established intervention description frameworks (such as TIDieR).^188^

Drawing on constructs from NPT,^84^ our review findings align closely with implementation studies in other healthcare settings that show that staff motivation, uptake and buy-in to DC-CON are contingent on this supportive infrastructure (collective action), but also on a clear sense of purpose and perceived benefit of DC-CON (coherence and cognitive participation) which is enhanced when staff are able to contribute to service design and receive feedback on outcomes or benefits (reflexive monitoring).^44 49 57 181 189^ Given the emphasis currently on safety, equity and person-centred care in UK maternity services, our review suggests that processes are needed to provide staff with confidence in using DC-CON such as private workspaces, training, IT support, clear protocols and strategies for providing feedback on outcomes and performance.^44 81 190^

### O—Optimising consultations to be responsive, flexible and personalised to different needs and preferences

The synthesis indicates that DC-CON has the potential to offer benefits in terms of reducing the BoC for women, and, drawing on candidacy theory, may make it easier (for some) to access services by reducing logistical barriers and enhancing a sense of eligibility to make contact with a professional.^45 63 64^ As noted in studies across a range of healthcare settings, by offering the ability to promote control, flexibility and fit with women’s needs, DC-CON has the potential to enhance uptake and engagement with maternity services and offers an important strategy for person-centred care delivery.^65 181 191^ However, as demonstrated in other studies, the review shows that women’s preferences for DC-CON are highly variable, dependent on personal characteristics, clinical and domestic circumstances.^48 49^ All stakeholder groups emphasised the importance of offering women informed choices around DC-CON and revisiting preferences throughout the maternity pathway. Negative experiences reported during COVID-19 may be (partially at least) linked to the lack of choice during this time. To date, the issue of choice and how to practically offer and integrate choice within remote care practices has not been investigated in detail. Knowledge users were agreed that individual women’s preferences and choices should ideally be explored (in-person) in the initial antenatal booking appointment, although it is currently unclear what questions should be asked or how best to document women’s preferences and needs. These questions require urgent attention in future research and service development.

### R—recognising the importance of access and inclusion

A key issue for maternity services is tackling inequalities in access and outcomes.^39 14^,^16^ Indeed, an increasing number of studies across different healthcare settings are highlighting the potential for remote consultations to impact equity and inclusion, but the associated mechanisms remain undertheorised.^5886 151 192^,^199^ Drawing on concepts associated with burden of treatment and candidacy theory,^4562^,^64^ our review contributes to this emerging evidence base. The findings highlight that DC-CON adds an additional, potentially complex, dimension to navigation of services as it requires key capabilities in health and digital literacy. Some groups of women are disadvantaged by lower levels of literacy; services should develop clear signposting and communication around DC-CON processes (eg, which phone numbers to use or how to use video platforms). As noted in other studies and reviews,^55 73 74 150 184 190^ our findings highlight the role of communication challenges (eg, associated with language, disability or neurodiversity) and access to digital and material resources (eg, internet, mobile phones or data) as key contexts that may impede women’s ability to use DC-CON, leading to frustration, anxiety, poor communication, potential misunderstandings and disengagement with services.^176 177^ To date, there has been little work on how best to address some of these challenges in relation to remote care (eg, how interpretation services can be optimally used with DC-CON).^184^ This is an important area for future research and service development.^190^ However, stakeholders cautioned that it is important not to make assumptions in relation to DC-CON suitability, as this risks unfairly excluding some women from the benefits that DC-CON may offer. Rather, it is important to ensure that access and inclusion questions are incorporated into assessments of individual women’s preferences and circumstances.

As noted in the Results section, many included studies in the review had not collected, disaggregated or specifically analysed data in relation to key sociodemographic characteristics (eg, ethnicity or socioeconomic status). Going forward, it is imperative for future research on DC-CON to better record ethnicity and sociodemographic data and to undertake intersectional analyses in order to embed an equity focus.^13 58 86^

### E—enabling quality and safety through relationship-focused connections

A key finding from COVID-19 related studies of DC-CON in maternity care was a concern that DC-CON may pose clinical safety risks and would negatively affect interpersonal relationships.^16 127 200 201^ Regarding the former, there is little evidence directly linking DC-CON to harms or adverse events, with most studies (both pre- and during COVID-19) suggesting equivalent clinical and satisfaction outcomes.^3^,^68 9 11 202^ However, given the poor reporting of specific intervention processes within many existing studies and a paucity of RCTs that include associated process evaluations,^108 164^ the mechanisms through which risk or safety operate need further careful exploration. For example, it is unclear how ‘safety-netting’ advice is best delivered or understood in a remote maternity context. Of note, only eight sources in the review focused specifically on triage systems, which (in the UK), are playing an increasing role as an initial access point to maternity services.^4680 102 105 106 114^,^116 169^ More research on triage as a distinct DC-CON modality may thus be warranted.^190^ Future research should include safety-focused outcomes measures. In addition, we suggest that consultation modality should become a reporting criterion within existing safety reporting structures and investigations of adverse outcomes. This would enable comparison of outcomes related to different consultation modes.

Drawing on Candidacy theory,^13 45^ the realist approach taken in the current review offers three new insights for further exploration. First, it suggests that a personalised and flexible approach to DC-CON can potentially act as a safeguard to safety and risk concerns. Professionals and women both stressed the importance of having the choice and autonomy to make adjudications about appropriate consultation modality, particularly having the option to visualise each other via video or to request a face-to-face consultation when indicated.^13^ Second, clinical and care-seeking decision-making as well as the confidence to disclose concerns were strongly linked to the nature of the clinical relationship.^203^ The review findings are consistent with evidence from other healthcare settings that DC-CON is best used in the context of an established relationship.^65173 184 204^,^207^ Indeed, where there is an element of continuity of care, the review suggests that DC-CON can enhance care delivery when used alongside other approaches (such as remote monitoring or to provide additional support to vulnerable groups).^207^ In these situations, DC-CON can help to motivate women/families to be involved in, and engage with, care and offers an important additional mechanism to provide reassurance.^65 150 151 207^ We suggest that future research would benefit from consideration of how DC-CON can optimise principles of relational continuity, establishing trust, mutual understanding and a sense of affiliation within varying models of maternity care.^203 207^

Finally, the review points to the importance of professionals developing good communication skills that are tailored to a remote context. The Institute for Healthcare Improvement refers to this as a good ‘*web-side manner’*.^181^ More research is needed (eg, using conversation analysis) to investigate the ways in which communication may change and may need to be adapted in the context of maternity DC-CON.^204 205^ Health professional education (preregistration and post registration) should include remote communication training for staff.^190^ Going forward, it will be important to develop patient-reported experience and outcome measures around DC-CON to evaluate quality of care offered through this route.

## Recommendations

Table 3 summarises recommendations for: (1) service delivery, (2) policy/systems development and (3) research in relation to the CORE principles of DC-CON implementation.

**Table 3:** Recommendations

For service delivery(maternity practitioners, students, managers, IT staff)	For policy/systems(managers, IT developers)	For research, evaluation, audit(researchers)
Creating the right environment, infrastructure and support for staff		
**Technology and equipment** Easily available IT support Good, secure internet connections Provision of work phones **Environment** Enable privacy and a quiet environment **Protocols/guidance** Develop protocols to support practice, to set out suitability criteria for DC-CON, to provide clarity around risks/safety/safeguarding issues (and guidance for how to address these) **DC-CON modality** Enable/allow staff choice and flexibility to use different DC-CON modalities according to professional judgement **Workload** Provide dedicated time for DC-CON (eg, with appropriate time allocated within workload models and job plans) **Training** Provide preregistration and postregistration training—for (1) confidence with systems/technology and (2) on communication (web-side manner) **Communication/feedback systems** Undertake audit/patient experience surveys and outcome data in order to create feedback processes to support staff buy-in and involvement Consider use of digital champions to promote change and support staff	Apps and systems for DC-CON to be codesigned with relevant knowledge users Apps and systems to have templates for recording of preferences and digital access/inclusion needs Apps and systems to provide users with information of DC-CON times and modality Interoperability for systems within NHS (eg, record systems and Apps) Interoperability with main-stream virtual platforms (eg, Whatsapp, Zoom) Clarity on GDPR and DC-CON systems	Undertake service evaluation to understand staff/service user and management perspectives and data on DC-CON uptake Development of audit criteria around good practice principles Guided by NPT, undertake case study research to better understand DC-CON implementation processes and challenges in different settings and with different staff groups (especially related to staff workload and efficiency) Future research on DC-CON should provide in-depth clear descriptions of interventions modality (video/phone), service setting and precisely how it is used within a service. Suggest the use of TIDieR framework for future intervention reporting
Optimising consultations to be responsive, flexible and personalised to different needs and preferences		
**Assessment, documentation and evaluation** Assess women’s: (1) preferences, (2) digital literacy/resources, (3) digital capacity/competency and (4) bio/psycho/social situation and needs (preferably in-person at the antenatal booking appointment) Record preferences/situation in notes Reassess suitability criteria/preferences/needs regularly **Informed choice** Produce information resources for women explaining the pros/cons of different DC-CON modalities and explaining how to use these modalities and when (including clarity around phone numbers for different services and who to call when) Offer women choice around consultation modality **DC-CON modality (video/phone**) Use DC-CON modality flexibly—as appropriate to women’s preferences and situation **DC-CON timing** Where possible, offer a time slot so that women are able to engage with the call		Development and evaluation of key assessment questions for use in booking appointments regarding women’s preferences, situation, capacity and access to digital and other supportive resources Development of Patient Reported Outcome Measures for DC-CON Research to investigate women’s and staff experiences focused more specifically on (1) particular groups of women with specific conditions or needs, or (2) at specific points in the maternity pathway, or (3) in specific services—to move away from highly generalised (and therefore less useful) COVID-19 research As above, case study research on different models of hybrid care—seeking to develop deeper theoretical understandings of how to achieve personalised care using DC-CON within a complex population-based system, including a consideration of ‘Burden of Care’ within systems or care models that use a greater proportion of DC-CON
Recognising the Importance of access and inclusion		
As above. Also: pay particular attention to needs associated with: health literacy and understanding of NHS systems, processes and services associated with maternity care digital literacy access to digital resources identification of specific barriers, needs or issues related to: migration status, language, neurodiversity, hearing impairment and other relevant characteristics **Interpretation** Ensure there is access to appropriate interpretation services Ensure that staff are trained to be confident and competent in making full use of virtual interpretation technologies	Ensure that Apps and systems have templates for recording of EDI data, DC-CON preferences and digital access/inclusion needs	As above. Also: Ensure that EDI data are adequately documented and analysed in order for intersectional analyses or more nuanced statistical analysis to be applied in relevant research Research to apply and develop existing theories related to inequality (such as candidacy or intersectionality) to DC-CON Research to explore the use of different interpretation technologies that can be used with DC-CON
Enabling quality and safety through relationship-focused connections		
As above. Also: Ensure there are opportunities for in-person consultations to enable thorough biopsychosocial assessments (including for safeguarding concerns) and relationship building Where appropriate, build in processes for utilisation of DC-CON to support relationship-based reassurance, involvement and engagement in care, including with partners/families Within protocols and guidance: develop DC-CON suitability criteria—but always ensure that staff have flexibility and autonomy to exercise professional judgement if there are any concerns	Development of clear and consistent hybrid pathways/protocols (with built-in flexibility options)	As in(O)above. Also: Experimental study designs—ideally RCTs—to investigate clinical, safety, access and equity outcomes for specific conditions, specific DC-CON technologies/modalities and specific population groups, including specific research on maternity triage systems and safety-netting processes Development of better theory-informed understandings of: (1) how to support and conceptualise relationship building and rapport within DC-CON, including (2) how to understand and support relational continuity in the context of different DC-CON systems within maternity services

DC-CONDigital Clinical ConsultationNHSNational Health ServiceNPTNormalisation Process Theory

## Reflexivity

A key feature of transparency and rigour in the review process relates to reflexivity, whereby the review team considers their identity and positionality and reflects on how these impacted the review focus and processes.^208 209^ The review team is multiprofessional (eg, obstetrics, midwifery, academic, information science, methodologists and teacher) and multidisciplinary (eg, political science, social science, applied health services research and psychology) combining different genders, ages and ethnicities. The diversity in the team was openly discussed and harnessed in four ways.^210^ First, to challenge assumptions and to debate key concepts as part of abductive theorising (eg, debating the meaning of taken-for-granted concepts such as continuity of care or DC-CON). Second, to advance retroductive theorising (eg, by reconceptualising the concept of ‘burden of treatment’ to a more maternity-relevant ‘burden of care’).^211 212^ Third, to develop analytical sensitivity and clarification of standpoint (eg, by developing a greater sensitivity and commitment to issues of equity, inclusivity and diversity).^213^ Fourth, to inform our research practices. In relation to the latter point, we undertook various strategies to address the potential power dynamics within and between knowledge user groups (and also between these groups and the research team). Furthermore, we recognised that meaningful involvement required an element of capacity development around the realist approach. Each knowledge user meeting began with brief, relatively simple, descriptions of the realist review approach, with opportunities for questions and clarifications. In order to aid accessibility and meaningfulness, the IPTs were developed into everyday scenarios (rather than presented as abstract theories) that we hoped that different groups would relate to.^41^ The service user and professional knowledge user group meetings were held separately. This was initially done to try and ensure that each group felt safe and comfortable to talk openly. To further promote comfort, the COSU-G groups were cofacilitated by the team’s public representative (CS) and the HCP-G were cofacilitated by team members who were midwives or obstetricians. We had initially planned to hold later consultation events with both groups together to stimulate dialogue, but decided against this in order to maintain the safe spaces that we felt we had created.^213^ After each meeting, group attendees were sent a summary of key points and insights elucidated by the team and were invited to comment further.

## Strengths and limitations

This was an extremely comprehensive and rigorously undertaken review, drawing on diverse sources of evidence and knowledge user expertise.^30 42^ The contribution of knowledge users was critical in overcoming potential academic biases in interpretations, identifying knowledge gaps, developing/validating the final PTs and formulating actionable recommendations. Nonetheless, an ongoing challenge and potential limitation lies in how to define, and adequately involve, knowledge users when considering an entire maternity system, covering an enormous range of different service contexts, staff roles and service users. Project meetings were mostly undertaken online, thereby facilitating participation of knowledge users from across the UK. However, we also conducted two consultations with women in-person, in an attempt to overcome barriers related to language and digital exclusion. Inevitably, there may be experiences, identities and groups that we may have missed.

A key strength, but also potential limitation, lies in the review’s wide focus across the whole maternity system. This focus helped to identify key implementation principles that can apply across settings. However, as noted previously, a limitation of many included evidence sources was their generic focus, lacking in detail of setting or precise intervention systems/processes. As described in the project protocol^26^ and in line with realist methods guidance,^30 37 42^ the team addressed this challenge through the application of abductive and retroductive reasoning, drawing together ‘nuggets of information’^90^ to infer wider explanatory claims, including attention to potential refutational theories.^37 42^ In addition, the consistency of the wider non-maternity evidence on DC-CON with the review findings lends an element of confidence to our theorising. Nonetheless, we recognise that the contextual diversity and imprecision of reporting in the underpinning studies means that the review is limited in being able to tailor its findings to specific microlevel contexts (eg, a specific service or a specific group).

A potential limitation is that it is possible that, by restricting the phase 2 search to 2016–onwards, relevant studies were missed. However, the radical change to DC-CON in terms of technology as well as user confidence/competence brought about by the COVID-19 pandemic means that earlier studies are less likely to offer contemporary insights unless based strongly in theory (of which we found very few in the 2010–onwards phase 1 maternity-specific search).

Another potential limitation is the emphasis given to the UK context within the review’s appraisal process, in which UK studies were prioritised for inclusion based on relevance. This was an intentional bias as the review’s purpose was to inform UK NHS policy and practice. Nonetheless, the majority of the evidence was drawn from non-UK settings; hence, we suggest that its findings may have international relevance. While studies from various OECD countries were included in the review, each PT included a mix of evidence from different OECD countries. Key themes were often repeated in these different contexts and did not appear to be strongly linked to particular models of care or financing systems. In terms of supporting evidence, no PT was heavily skewed by evidence from a particular country or region. Our ability to theorise the implications of international contextual variation may have been strengthened further had we included international experts on the advisory panel.

A significant strength of this review is the attention given to mid-range theories. This enabled the development of more analytical interpretations of the evidence, leading to PTs that are theoretically transferable, and, hopefully, can be applied across a range of maternity and geographical contexts. There are, of course, a wide range of other theories that could have been considered and that would also offer insight. Given the time constraints of the project, we adopted a pragmatic ‘best-fit’ (rather than exhaustive) approach to theory selection,^214^ recognising that other review teams may have made different decisions.

## Conclusions

In the UK, as in other contexts, digital transformation forms a key aspiration within maternity and health system reform.^24 25 215^ The PTs developed in this review offer important new insights that can guide further policy, research and service developments in this area. The reviews affirms the importance placed by stakeholders on flexibility and choice, delivered through inclusive, person-centred, relational care approaches. A key challenge for the future lies in how to incorporate these principles into the design of new ‘hybrid’ models of care.

## supplementary material

10.1136/bmjopen-2023-079153 online supplemental file 1

10.1136/bmjopen-2023-079153 online supplemental file 2

10.1136/bmjopen-2023-079153 online supplemental file 3

10.1136/bmjopen-2023-079153 online supplemental file 4

10.1136/bmjopen-2023-079153 online supplemental file 5

10.1136/bmjopen-2023-079153 online supplemental file 6

10.1136/bmjopen-2023-079153 online supplemental file 7

10.1136/bmjopen-2023-079153 online supplemental file 8

10.1136/bmjopen-2023-079153 online supplemental file 9

10.1136/bmjopen-2023-079153 online supplemental file 10

10.1136/bmjopen-2023-079153 online supplemental file 11

10.1136/bmjopen-2023-079153 online supplemental file 12

10.1136/bmjopen-2023-079153 online supplemental file 13

10.1136/bmjopen-2023-079153 online supplemental file 14

## Data Availability

All data relevant to the study are included in the article or uploaded as supplementary information.
