# Supplementary material for: Optimising digital clinical consultations in maternity care: a realist review and implementation principles
Source: BMJ Open. 2024 Nov 1;14(10):e079153. doi: 10.1136/bmjopen-2023-079153 (PMC11529580; doi:10.1136/bmjopen-2023-079153)
Supplement: online supplemental file 2 [file bmjopen-14-10-s002.pdf]

Supplemental File 2: Glossary of Realist Terms

Concept	Definition/Description
Realist Review (also referred to as realist synthesis)	A theory-driven approach for synthesising secondary research and providing explanations for why and how interventions work, for whom, when and in what contexts.
Intervention	The features and characteristics of programmes, e.g. technology used, staff involved.
Context (C)	The conditions or circumstances required for programme mechanisms to activate and impact the outcomes - i.e. the environment in which digital consultations happen.
Mechanisms (M)	The resources offered in a programme and how people respond to them. Mechanisms can be behavioural, cognitive, emotional etc.
Outcomes (O)	The intended or unintended effects of the context-mechanism interaction.
Context-Mechanism-Outcome (**CMO**) configuration	A heuristic used in realist reviews to help express causal insights which explain how an intervention, or part of an intervention, works. CMO configurations are the building blocks for programme theories.
Initial Programme Theory (IPT)	A hypothesised programme theory for how and why a programme works; usually tested against the literature and through consultation.
Programme Theory (PT)	An explanation for how and why a programme works. A realist review attempts to develop and test programme theories.
Mid-range Theory (MRT)	These can help to conceptualise and understand what is happening between contexts and mechanisms to produce outcomes. MRTs are more general than programme theories and may be applicable across similar programmes occurring in different settings.
