# Supplementary material for: Optimising digital clinical consultations in maternity care: a realist review and implementation principles
Source: BMJ Open. 2024 Nov 1;14(10):e079153. doi: 10.1136/bmjopen-2023-079153 (PMC11529580; doi:10.1136/bmjopen-2023-079153)
Supplement: online supplemental file 3 [file bmjopen-14-10-s003.pdf]

Supplemental File 3: GRIPP2 Checklist

* The shortform of the checklist has been used as the long form contained many items that were not relevant for a realist review

**Section and Topic**	**Item**	**Reported on Page No.**
1. Aim	Report the aim of PPI in the study	• p.4
2. Methods	Provide a clear description of the methods used for PPI in the study	• p.4<br>• Supplemental File 4
3. Study results	Outcomes—Report the results of PPI in the study, including both positive and negative outcomes	• p.4<br>• p.8
4. Discussion and conclusion	Outcomes—Comment on the extent to which PPI influenced the study overall. Describe positive and negative effects	• p.24
5. Reflections/critical perspective	Comment critically on the study, reflecting on the things that went well and those that did not, so others can learn from this experience	• p.24

Reference

Staniszewska, S., Brett, J., Simera, I. *et al.* GRIPP2 reporting checklists: tools to improve reporting of patient and public involvement in research. *Res Involv Engagem* **3**, 13 (2017). https://doi.org/10.1186/s40900-017-0062-2
