# Supplementary material for: Optimising digital clinical consultations in maternity care: a realist review and implementation principles
Source: BMJ Open. 2024 Nov 1;14(10):e079153. doi: 10.1136/bmjopen-2023-079153 (PMC11529580; doi:10.1136/bmjopen-2023-079153)
Supplement: online supplemental file 4 [file bmjopen-14-10-s004.pdf]

Supplemental File 4: Information about Knowledge Users Involved in the Review

Knowledge User Groups participated in 3 consultation exercises (one in each Phase of the review). The Nottingham Maternity Research Network was also part of a research prioritisation exercise undertaken during the Covid-19 pandemic in which digital clinical consultations were identified as an important area for future research.[1]

All knowledge user group participants were requested to complete a demographic questionnaire via MS Forms. In spite of multiple reminders, not all of the participants completed the form. Hence, the demographic data is incomplete.

Community Organisation and Service User Group (COSU-G)

Thirteen women were part of this group, recruited through: Nottingham Maternity Research Network (n=7), National Autistic Society (n=1) and Sister Circle (n=5). Five of these women had been pregnant or had babies during the last 2 years.

Demographic Data

Six women completed the questionnaire. The age range of these six respondents was 23-55 years old, with a mean age of 36.8 years old. Two respondents were born in Scotland, one in Egypt, one in Canada, one in England and one in the UK. Three identified their ethnicity as White (one specified Scottish), one as ‘Other ethnic group’ (specified as Arab), one as Black, African, Caribbean or Black British (specified as Caribbean) and one preferred not to say. Three respondents stated that they had no religion, one identified as Muslim, one as a Christian (including Church of England, Catholic, Protestant and all other Christian denominations) and one chose not to say. Four COSU-G respondents spoke English as their first language, one spoke Arabic as their first language and one preferred not to say. One identified as having a disability, four stated no disability and one chose not to say. When asked if they had any other physical or mental health conditions or illnesses which they felt were relevant to their experience with the maternity services, four answered with ‘no’, one answered with ‘yes’ and one preferred not to say. Three respondents stated that they worked in ‘Higher & intermediate managerial, administrative, professional occupations’, two were in full-time education and one chose not to say.

Health Professional Group

Twenty-six health professionals were part of this group, representing 20 midwives and 6 obstetricians.

Demographic Data

Eleven individuals (42%) completed the questionnaire. Respondents’ age ranged from 32-60 years old, with a mean age of 45.7 years (one respondent did not answer). Nine respondents were born in the UK, one from New Zealand and one from Spain. Eight respondents identified their ethnicity as White, two as Black, African, Caribbean or Black British (one of which further specified as West African). Seven identified as Christians (including Church of England, Catholic, Protestant and all other Christian denominations), two as having no religion and two chose not to say. Nine respondents spoke English as their first language, one spoke Spanish as their first language and one preferred not to say.

Additional Consultations with Women

In Phase 3 of the review, 3 additional consultation exercises were held with groups of women recruited through different community organisations: Sister Circle (London), City of Sanctuary (Bradford) and the Centre for Ethnic Health Research (Leicester). In total, the

groups comprised 22 women, all of whom were pregnant or had had a baby within the last 2 years.

Sister Circle (London)

6 women – all asylum seekers or refugees from Pakistan, Mexico and Eritrea.

City of Sanctuary (Bradford)

10 women – all refugees and asylum seekers from a variety of countries.

Centre for Ethnic Health Research (Leicester)

6 women – all of South Asian heritage; 2 of whom had limited English and took part with the aid of an interpreter.

References

1. Evans K, Janiszewski H, Evans C, et al. Establishing information needs and research priorities in response to the Covid-19 pandemic in the local maternity setting. *Midwifery* 2021; 95: 102922. 2021/01/31. DOI: 10.1016/j.midw.2021.102922.
