# Supplementary figures and images for: Optimising digital clinical consultations in maternity care: a realist review and implementation principles
Source: BMJ Open. 2024 Nov 1;14(10):e079153. doi: 10.1136/bmjopen-2023-079153 (PMC11529580; doi:10.1136/bmjopen-2023-079153)

Supplemental File 5: Flowchart

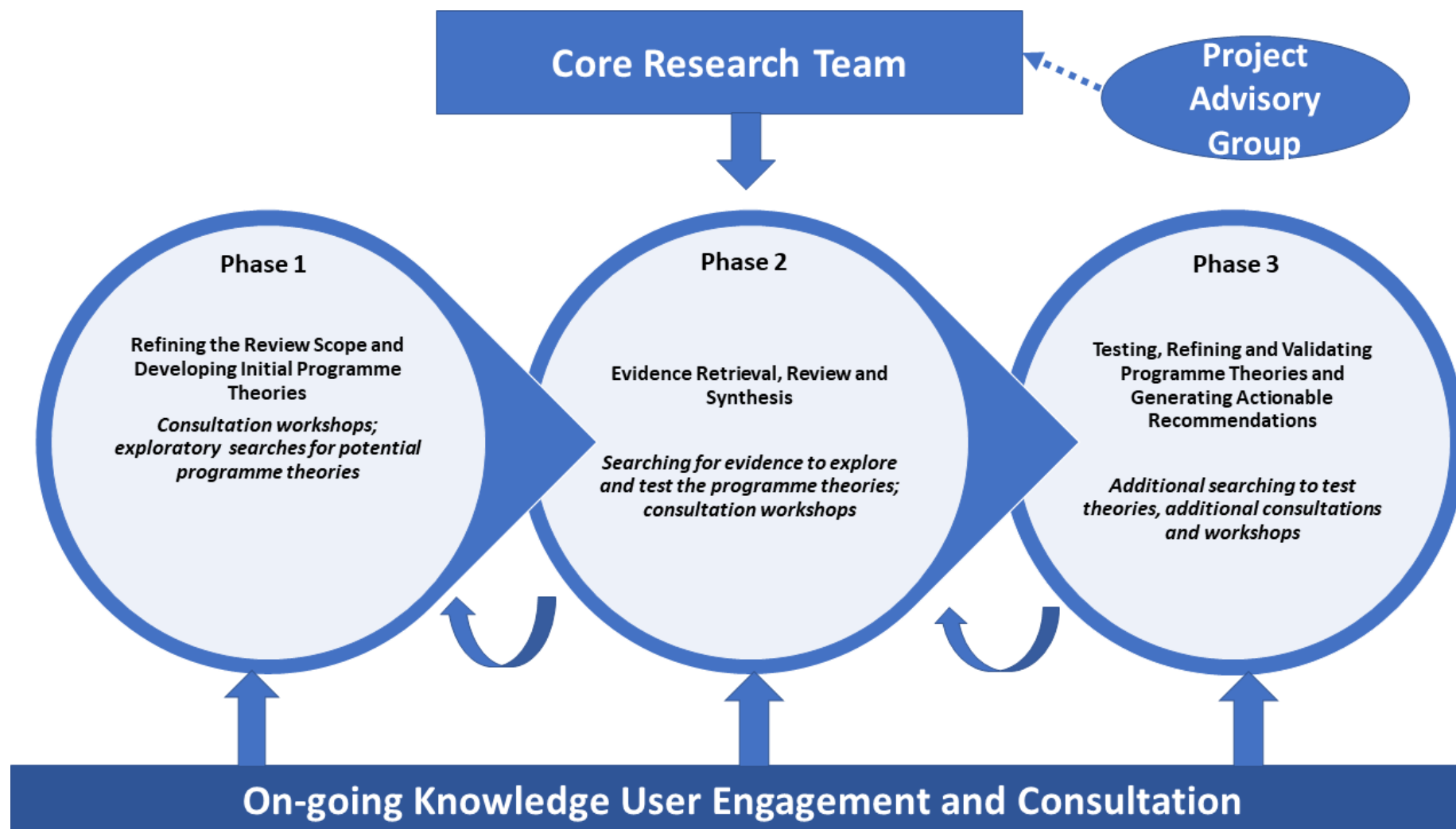

Supplement: online supplemental file 5 [file bmjopen-14-10-s005.pdf]
