# Supplementary material for: Optimising digital clinical consultations in maternity care: a realist review and implementation principles
Source: BMJ Open. 2024 Nov 1;14(10):e079153. doi: 10.1136/bmjopen-2023-079153 (PMC11529580; doi:10.1136/bmjopen-2023-079153)
Supplement: online supplemental file 9 [file bmjopen-14-10-s009.pdf]

Supplemental File 9: Phase 2 Search Strategies

Search Strategies for Comprehensive Database Searches (Phase 2)

Ovid MEDLINE(R) and Epub Ahead of Print, In-Process, In-Data-Review & Other Non-Indexed Citations, Daily and Versions <1946 to June 29, 2022>

1 exp Telemedicine/ 40937

2 remote consultation/ or videoconferencing/ 7451

3 (telemedicine or tele-medicine or telecare or tele-care or telehealth or tele-health or telemonitoring or tele-monitoring or remote monitoring).mp. 50441

4 ((remote* or virtual* or online or on-line or digital*) adj3 (consultation* or appointment* or meet*)).mp. 9066

5 (videoconferenc* or video-conferenc* or teleconferenc* or tele-conferenc* or zoom or facetime or face-time or badge?net or medway or system C or systemC or K2* or athena or attendanywhere or attend anywhere or dr doctor or doctor doctor or PKB or patient knows best or PAS or patient administration system* or near me).mp. 74138

6 1 or 2 or 3 or 4 or 5 128601

7 exp Maternal Health Services/ 55880

8 exp Prenatal Care/ or exp Midwifery/ or exp Pregnancy/ or exp Obstetrics/ 991039

9 (matern* or pregnan* or prenatal or pre-natal or antenatal or ante-natal or perinatal or peri-natal or postnatal or post-natal or postpartum or post-partum or breastfeed* or breast feed* or infant feeding or lactati* or midwi* or obstetric* or gestation*).mp. 1506581

10 7 or 8 or 9 1517372

11 6 and 10 4898

12 limit 11 to yr="2016 -Current" 2812

13 meta analysis.mp,pt. or review.pt. or search:.tw. 3449244

14 12 and 13 462

15 12 not 13 2350

16 exp Great Britain/ 385304

17 (national health service* or nhs*).ti,ab,in. 247302

18 (english not ((published or publication* or translat* or written or language* or speak* or literature or citation*) adj5 english)).ti,ab. 45087

19 (gb or "g.b." or britain* or (british* not "british columbia") or uk or "u.k." or united kingdom* or (england* not "new england") or northern ireland* or northern irish* or scotland* or scottish* or ((wales or "south wales") not "new south wales") or welsh*).ti,ab,jw,in. 2322787

20 (bath or "bath's" or ((birmingham not alabama*) or ("birmingham's" not alabama*) or bradford or "bradford's" or brighton or "brighton's" or bristol or "bristol's" or carlisle* or "carlisle's" or (cambridge not (massachusetts* or boston* or harvard*)) or ("cambridge's" not (massachusetts* or boston* or harvard*))

or (canterbury not zealand*) or ("canterbury's" not zealand*) or chelmsford or "chelmsford's" or chester or "chester's" or chichester or "chichester's" or coventry or "coventry's" or derby or "derby's" or (durham not (carolina* or nc)) or ("durham's" not (carolina* or nc)) or ely or "ely's" or exeter or "exeter's" or gloucester or "gloucester's" or hereford or "hereford's" or hull or "hull's" or lancaster or "lancaster's" or leeds* or leicester or "leicester's" or (lincoln not nebraska*) or ("lincoln's" not nebraska*) or (liverpool not (new south wales* or nsw)) or ("liverpool's" not (new south wales* or nsw)) or ((london not (ontario* or ont or toronto*)) or ("london's" not (ontario* or ont or toronto*)) or manchester or "manchester's" or (newcastle not (new south wales* or nsw)) or ("newcastle's" not (new south wales* or nsw)) or norwich or "norwich's" or nottingham or "nottingham's" or oxford or "oxford's" or peterborough or "peterborough's" or plymouth or "plymouth's" or portsmouth or "portsmouth's" or preston or "preston's" or ripon or "ripon's" or salford or "salford's" or salisbury or "salisbury's" or sheffield or "sheffield's" or southampton or "southampton's" or st albans or stoke or "stoke's" or sunderland or "sunderland's" or truro or "truro's" or wakefield or "wakefield's" or wells or westminster or "westminster's" or winchester or "winchester's" or wolverhampton or "wolverhampton's" or (worcester not (massachusetts* or boston* or harvard*)) or ("worcester's" not (massachusetts* or boston* or harvard*)) or (york not ("new york*" or ny or ontario* or ont or toronto*)) or ("york's" not ("new york*" or ny or ontario* or ont or toronto*))))).ti,ab,in. 1633647

21 (bangor or "bangor's" or cardiff or "cardiff's" or newport or "newport's" or st asaph or "st asaph's" or st davids or swansea or "swansea's").ti,ab,in. 65320

22 (aberdeen or "aberdeen's" or dundee or "dundee's" or edinburgh or "edinburgh's" or glasgow or "glasgow's" or inverness or (perth not australia*) or ("perth's" not australia*) or stirling or "stirling's").ti,ab,in. 240883

23 (armagh or "armagh's" or belfast or "belfast's" or lisburn or "lisburn's" or londonderry or "londonderry's" or derry or "derry's" or newry or "newry's").ti,ab,in. 31250

24 or/16-23 2915825

25 (exp africa/ or exp americas/ or exp antarctic regions/ or exp arctic regions/ or exp asia/ or expoceania/) not (exp great britain/ or europe/) 3033847

26 24 not 25 2777210

27 14 and 26 101

28 14 not 26 361

29 15 and 26 225

30 15 not 26 2125

Embase <1974 to 2022 June 29>

1 exp Telemedicine/ 60758
2 teleconsultation/ or videoconferencing/ 19979
3 (telemedicine or tele-medicine or telecare or tele-care or teleconsultation* or tele-consultation* or telehealth or tele-health or telemonitoring or tele-monitoring or remote monitoring).mp. 77484
4 ((remote* or virtual* or online or on-line or digital*) adj3 (consultation* or appointment* or meet*)).mp. 5895
5 (videoconferenc* or video-conferenc* or teleconferenc* or tele-conferenc* or zoom or facetime or face-time or badge?net or medway or system C or systemC or K2* or athena or attendanywhere or attend anywhere or dr doctor or doctor doctor or PKB or patient knows best or PAS or patient administration system* or near me).mp. 111335
6 1 or 2 or 3 or 4 or 5 192510
7 exp Maternal Health Services/ 2468
8 exp Prenatal Care/ or exp Midwifery/ or exp Pregnancy/ or exp Obstetrics/ 857303
9 (matern* or pregnan* or prenatal or pre-natal or antenatal or ante-natal or perinatal or peri-natal or postnatal or post-natal or postpartum or post-partum or breastfeed* or breast feed* or infant feeding or lactati* or midwi* or obstetric* or gestation*).mp. 1707141
10 7 or 8 or 9 1715189
11 6 and 10 6923
12 limit 11 to yr="2016 -Current" 4195
13 meta-analys:.mp. or search:.tw. or review.pt. 3577645
14 exp United Kingdom/ 445209
15 (national health service* or nhs*).ti,ab,in,ad. 424909
16 (english not ((published or publication* or translat* or written or language* or speak* or literature or citation*) adj5 english)).ti,ab. 53436
17 (gb or "g.b." or britain* or (british* not "british columbia") or uk or "u.k." or united kingdom* or (england* not "new england") or northern ireland* or northern irish* or scotland* or scottish* or ((wales or "south wales") not "new south wales") or welsh*).ti,ab,jx,in,ad. 3507772
18 (bath or "bath's" or ((birmingham not alabama*) or ("birmingham's" not alabama*) or bradford or "bradford's" or brighton or "brighton's" or bristol or "bristol's" or carlisle* or "carlisle's" or (cambridge not (massachusetts* or boston* or harvard*)) or ("cambridge's" not (massachusetts* or boston* or harvard*)) or (canterbury not zealand*) or ("canterbury's" not zealand*) or chelmsford or "chelmsford's" or chester or "chester's" or chichester or "chichester's" or coventry or "coventry's" or derby or "derby's" or (durham not (carolina* or nc)) or ("durham's" not (carolina* or nc)) or ely or "ely's" or exeter or "exeter's" or gloucester or "gloucester's" or hereford or "hereford's" or hull or "hull's" or lancaster or "lancaster's" or leeds* or leicester or "leicester's" or (lincoln not nebraska*) or ("lincoln's" not nebraska*) or (liverpool not (new south wales* or nsw)) or ("liverpool's" not (new south wales* or nsw)) or ((london not (ontario* or ont or toronto*)) or ("london's" not (ontario* or ont or toronto*)) or manchester or "manchester's" or (newcastle not (new south wales* or nsw)) or ("newcastle's" not (new south wales* or nsw)) or norwich or "norwich's" or nottingham or "nottingham's" or oxford or "oxford's" or peterborough or "peterborough's" or plymouth or "plymouth's" or portsmouth or "portsmouth's" or preston or "preston's" or ripon or "ripon's" or salford or "salford's" or salisbury or "salisbury's" or sheffield or "sheffield's" or southampton or "southampton's" or st albans or stoke or "stoke's" or sunderland or "sunderland's" or truro or

"truro's" or wakefield or "wakefield's" or wells or westminster or "westminster's" or winchester or "winchester's" or wolverhampton or "wolverhampton's" or (worcester not (massachusetts* or boston* or harvard*)) or ("worcester's" not (massachusetts* or boston* or harvard*)) or (york not ("new york*" or ny or ontario* or ont or toronto*)) or ("york's" not ("new york*" or ny or ontario* or ont or toronto*))))).ti,ab,in,ad. 2731888

19 (bangor or "bangor's" or cardiff or "cardiff's" or newport or "newport's" or st asaph or "st asaph's" or st davids or swansea or "swansea's").ti,ab,in,ad. 112122

20 (aberdeen or "aberdeen's" or dundee or "dundee's" or edinburgh or "edinburgh's" or glasgow or "glasgow's" or inverness or (perth not australia*) or ("perth's" not australia*) or stirling or "stirling's").ti,ab,in,ad. 375775

21 (armagh or "armagh's" or belfast or "belfast's" or lisburn or "lisburn's" or londonderry or "londonderry's" or derry or "derry's" or newry or "newry's").ti,ab,in,ad. 51742

22 14 or 15 or 16 or 17 or 18 or 19 or 20 or 21 4284822

23 (exp "arctic and antarctic"/ or exp oceanic regions/ or exp western hemisphere/ or exp africa/ or exp asia/ or exp "australia and new zealand"/) not (exp united kingdom/ or europe/) 3506781

24 22 not 23 4027067

25 12 and 13 607

26 12 not 13 3588

27 24 and 25 114

28 25 not 24 493

29 26 and 24 500

30 26 not 24 3088

31 limit 30 to conference abstract status 933

32 30 not 31 2155

APA PsycInfo <2002 to June Week 3 2022>

1 exp Telemedicine/ 10965
2 videoconferencing/ 719
3 (telemedicine or tele-medicine or teleconsultation or tele-consultation or telecare or tele-care or telehealth or tele-health or telemonitoring or tele-monitoring or remote monitoring).mp. 9851
4 ((remote* or virtual* or online or on-line or digital*) adj3 (consultation* or appointment* or meet*)).mp. 1509
5 (videoconferenc* or video-conferenc* or teleconferenc* or tele-conferenc* or zoom or facetime or face-time or badge?net or medway or system C or systemC or K2* or athena or attendanywhere or attend anywhere or dr doctor or doctor doctor or PKB or patient knows best or PAS or patient administration system* or near me).mp. 10774
6 1 or 2 or 3 or 4 or 5 23210
7 exp Prenatal Care/ or exp Midwifery/ or exp Pregnancy/ or exp Obstetrics/ 35626
8 (matern* or pregnan* or prenatal or pre-natal or antenatal or ante-natal or perinatal or peri-natal or postnatal or post-natal or postpartum or post-partum or breastfeed* or breast feed* or infant feeding or lactati* or midwi* or obstetric* or gestation*).mp. 113676
9 7 or 8 117406
10 6 and 9 663
11 limit 10 to yr="2016 -Current" 438
12 limit 11 to "reviews (maximizes sensitivity)" 297
13 11 not 12 141

ASSIA via ProQuest (searched 7/7/22)

(noft(telemedicine OR tele-medicine OR telecare OR tele-care OR telehealth OR tele-health OR telemonitoring OR tele-monitoring OR "remote monitoring") OR noft(remote NEAR/3 care) OR noft("remote consultation" OR videoconferencing OR video-conferenc* OR facetime OR zoom OR face-time OR medway OR "system C" OR systemC OR K2* OR athena OR attendanywhere OR "attend anywhere" OR "dr doctor" OR "doctor doctor" OR PKB OR "patient knows best" OR PAS OR "patient administration system*" OR "near me")) AND noft(matern* OR pregnan* OR prenatal OR pre-natal OR antenatal OR ante-natal OR perinatal OR peri-natal OR postnatal OR post-natal OR postpartum OR post-partum OR breastfeed* OR "breast feed*" OR "infant feeding" OR lactati* OR midwi* OR obstetric* OR gestation*) AND yr(2016-2022)

CINAHL (searched 1/7/22)

	Query	Limiters/Expanders	Results
S23	S17 not S18	Limiters - Published Date: 20160101-20221231<br>Expanders - Apply equivalent subjects<br>Search modes - Boolean/Phrase	163
S22	S16 not S18	Limiters - Published Date: 20160101-20221231<br>Expanders - Apply equivalent subjects<br>Search modes - Boolean/Phrase	546
S21	S14 not S15	Expanders - Apply equivalent subjects<br>Search modes - Boolean/Phrase	699
S20	S15 not S19	Expanders - Apply equivalent subjects<br>Search modes - Boolean/Phrase	1,210
S19	S16 OR S17	Expanders - Apply equivalent subjects<br>Search modes - Boolean/Phrase	757
S18	S16 AND S17	Expanders - Apply equivalent subjects<br>Search modes - Boolean/Phrase	48
S17	S5 AND S12	Limiters - Published Date: 20160101-20221231; Clinical Queries: Review - Best Balance<br>Expanders - Apply equivalent subjects<br>Search modes - Boolean/Phrase	211
S16	S5 AND S12	Limiters - Published Date: 20160101-20221231<br>Expanders - Apply equivalent subjects	594

		Narrow by SubjectGeographic: - uk & ireland<br>Search modes - Boolean/Phrase	
S15	S5 AND S12	Limiters - Published Date: 20160101-20221231<br>Expanders - Apply equivalent subjects<br>Search modes - Boolean/Phrase	1,967
S14	S5 AND S12	Limiters - Published Date: 20100101-20221231<br>Expanders - Apply equivalent subjects<br>Search modes - Boolean/Phrase	2,666
S13	S5 AND S12	Expanders - Apply equivalent subjects<br>Search modes - Boolean/Phrase	2,988
S12	S6 OR S7 OR S8 OR S9 OR S10 OR S11	Expanders - Apply equivalent subjects<br>Search modes - Boolean/Phrase	455,994
S11	matern* or pregnan* or prenatal or pre-natal or antenatal or ante-natal or perinatal or peri-natal or postnatal or post-natal or postpartum or post-partum or breastfeed* or "breast feed*" or "infant feeding" or lactati* or midwi* or obstetric* or gestation*	Expanders - Apply equivalent subjects<br>Search modes - Boolean/Phrase	451,291
S10	(MH "Obstetrics") OR (MH "Diagnosis, Obstetric+") OR (MH "Obstetric Service")	Expanders - Apply equivalent subjects<br>Search modes - Boolean/Phrase	33,685
S9	(MH "Pregnancy+")	Expanders - Apply equivalent subjects<br>Search modes - Boolean/Phrase	236,550
S8	(MH "Midwifery+")	Expanders - Apply equivalent subjects<br>Search modes - Boolean/Phrase	21,780
S7	(MH "Prenatal Care") OR (MH "Prenatal Care (Iowa NIC)")	Expanders - Apply equivalent subjects<br>Search modes - Boolean/Phrase	19,037

S6	(MH "Maternal Health Services+")	Expanders - Apply equivalent subjects Search modes - Boolean/Phrase	35,082
S5	S1 OR S2 OR S3 OR S4	Expanders - Apply equivalent subjects Search modes - Boolean/Phrase	56,506
S4	(telemedicine or tele-medicine or telecare or tele-care or telehealth or tele-health) OR (teleconsultation or tele-consultation or "video consultation*" or videoconsultation*) OR (videoconferenc* or video-conferenc* or teleconferenc* or tele-conferenc* or zoom or facetime or face-time or badgenet or "badge net" or medway or "system C" or systemC or K2* or athena or attendanywhere or "attend anywhere" or "dr doctor" or "doctor doctor" or PKB or "patient knows best" or PAS or "patient administration system*" or "near me"	Expanders - Apply equivalent subjects Search modes - Boolean/Phrase	49,775
S3	(MH "Videoconferencing+")	Expanders - Apply equivalent subjects Search modes - Boolean/Phrase	5,086
S2	(MH "Remote Consultation")	Expanders - Apply equivalent subjects Search modes - Boolean/Phrase	2,870
S1	(MH "Telemedicine+") OR (MH "Telehealth+")	Expanders - Apply equivalent subjects Search modes - Boolean/Phrase	32,293

COCHRANE LIBRARY (searched 3/7/22)

ID Search Hits
#1 MeSH descriptor: [Telemedicine] explode all trees
#2 MeSH descriptor: [Remote Consultation] explode all trees
#3 MeSH descriptor: [Videoconferencing] explode all trees
#4 (telemedicine or tele-medicine or telecare or tele-care or telehealth or tele-health or telemonitoring or tele-monitoring or "remote monitoring"):ti,ab,kw (Word variations have been searched)
#5 ((remote* or virtual* or online or on-line or digital*) near/3 (consultation* or appointment* or meet*)):ti,ab,kw (Word variations have been searched)
#6 (videoconferenc* or video-conferenc* or teleconferenc* or tele-conferenc* or zoom or facetime or face-time or badgenet or "badge net" or medway or "system C" or systemC or K2* or athena or attendanywhere or "attend anywhere" or "dr doctor" or "doctor doctor" or PKB or "patient knows best" or PAS or "patient administration system*" or "near me"):ti,ab,kw (Word variations have been searched)
#7 #1 or #2 or #3 or #4 or #5 or #6
#8 MeSH descriptor: [Maternal Health Services] explode all trees
#9 MeSH descriptor: [Prenatal Care] explode all trees
#10 MeSH descriptor: [Midwifery] explode all trees
#11 MeSH descriptor: [Pregnancy] explode all trees
#12 MeSH descriptor: [Obstetrics] explode all trees
#13 (matern* or pregnan* or prenatal or pre-natal or antenatal or ante-natal or perinatal or peri-natal or postnatal or post-natal or postpartum or post-partum or breastfeed* or "breast feed*" or "infant feeding" or lactati* or midwi* or obstetric* or gestation*):ti,ab,kw (Word variations have been searched)
#14 #8 or #9 or #10 or #11 or #12 or #13

Grey Literature Searches (Phase 2)

Searches to identify relevant unpublished evidence were conducted, including searching for theses via ProQuest Dissertations & Theses and ETHOS, and the DANS-Easy archive of European research data.

Dissertations & Theses was searched using a simple Boolean strategy:
(remote or virtual or online or telehealth) and (matern* or pregnan* or midwi*)
retrieving 227 results which were imported to EndNote for screening.

ETHOS functionality is more limited so searches were restricted to various pairings of words (e.g. **pregnant and online**; **telemedicine and pregnancy**; **virtual and obstetrics**) and a total of 6 records of possible interest were selected.

DANS-Easy yielded only 1 result not found via the previous searches.

In total, these resources retrieved 234 results for screening.

Other Grey Literature Search Strategies (Phase 2)

A list of twenty grey literature websites was compiled by the core research team and searched for relevant content on digital consultations in maternity care (see below); 28 texts were found and all were excluded; 17 texts were considered to have ‘no relevant data’, four texts could not be accessed, two reports and two guidance documents were already in the sample, two were non-UK discussion/opinion pieces and one was published pre-2016.

1. RCM
2. RCOG
3. NHSx
4. NCT
5. All4Maternity
6. MVP/National Maternity Voices
7. Maternity and Midwifery Forum
8. AIMS
9. Association of Radical Midwives
10. BirthRights
11. The Health Foundation
12. The King’s Fund
13. WHO
14. International Confederation of Midwives
15. European Midwives Association
16. British Association Perinatal Medicine (BAPM) and the Intrapartum Care group
17. Positive Birth Movement
18. Health Education England
19. Make Birth Better
20. Birth Trauma Association
