# Supplementary material for: Optimising digital clinical consultations in maternity care: a realist review and implementation principles
Source: BMJ Open. 2024 Nov 1;14(10):e079153. doi: 10.1136/bmjopen-2023-079153 (PMC11529580; doi:10.1136/bmjopen-2023-079153)
Supplement: online supplemental file 10 [file bmjopen-14-10-s010.pdf]

Supplemental File 10: Phase 2 Appraisal and Prioritisation Criteria

The overall appraisal approach was informed by various sources of literature.[1] Appraisal was based on 3 dimensions: relevance, rigour and richness. Relevance and rigour were assessed as high/moderate/low based on aggregate scores for 3 different criteria. The richness assessment was based on one criterion.

Appraisal Domain	Criteria		
**Relevance**<br>• **High = 5pts**<br>• **Moderate = 3pts**<br>• **Low = 1pt**	Does the text focus on DC-CON in UK maternity care?<br>(Yes = 5pts, No = 0pt)	Is the text a high, moderate or low match to the ARM@DA review questions/IPTs?	Does the text provide a high, moderate or low number of "nuggets" of information?
**Rigour**<br>• **High = 5pts**<br>• **Moderate = 3pts**<br>• **Low = 1pt**	Does the text provide a clear account of processes – ethics, sample, selection, limitations and biases noted?	Does the text include a clear description of analytical processes?	Does the text present a developed and plausible explanation?
**Richness**<br>• **High = 5pts**<br>• **Moderate = 3pts**<br>• **Low = 1pt**	Does the text offer a rich description, grounded in the data, of the process and context that can lead to explanatory insights?		

Operationalising Scoring Judgments

Relevance	Scoring Explanations
High - 5	The text is focussed on DC-CON in UK maternity care and has a high amount of "nuggets".
Moderate - 3	The text is either focused on DC-CON in UK maternity care with a moderate amount of "nuggets" or is focussed on DC-CON in an OECD country's maternity care system and has a high amount of "nuggets".
Low - 1	The text may be focussed on DC-CON in the UK or an OECD country and has a low number of "nuggets".

Rigor	Scoring Explanations
High - 5	The text contains a great amount of methodological detail, logical and appropriate explanations for support the conclusions.
Moderate - 3	The text contains some methodological detail and reasonable explanations to support the conclusions.
Low - 1	The text contains a limited or insufficient amount of methodological detail and it is unclear how the conclusions were reached.

[1] **Pawson, R. (2006)** 'Digging for Nuggets: How 'Bad' Research Can Yield 'Good' Evidence' *International Journal of Social Research Methodology* 9(2):127-142; **Rycroft-Malone, J., McCormack, B., Hutchinson, A. M. et al (2012)** 'Realist synthesis: Illustrating the method for implementation research' *Implementation Science* 7(1) 33; **Jagosh, J. (2022)** 'Appraisal Form Template' A*ccessed from workshop: Realist Synthesis Masterclass*; **Higginbottom, G., Evans, C. Morgan, M. et al. (2020)** 'Access to and interventions to improve maternity care services for immigrant women: a narrative synthesis systematic review' *Health Services and Delivery Research* 8(14): 24; **Dada S, Dalkin S, Gilmore B, et al. (2023)** Applying and reporting relevance, richness and rigour in realist evidence appraisals: Advancing key concepts in realist reviews. *Research Synthesis Methods* 2023.

Richness	Scoring Explanations
High - 5	The text contains a good or great amount of detail and depth to explain how and why an intervention does, or is expected to, work. i.e. there is a description of the theoretical underpinning/programme theory which allows the findings to be transferred to other settings.
Moderate - 3	The text contains a reasonable amount of detail and depth to explain how and why an intervention does, or is expected to, work.
Low - 1	The text contains a limited or insufficient amount of detail and depth to explain how and why an intervention does, or is expected to, work.

Below is a visual of the spreadsheet appraisal form – undertaken in Excel.

PRIORITY STUDIES		RELEVANCE: High = 5pts, Moderate = 3pts, Low = 1pt				RIGOUR: High = 5pts, Moderate = 3pts, Low = 1pt					RICHNESS: High = 5pts, Moderate = 3pts, Low = 1pt
Study:	Appraiser initials	Does the text focus on DC-CON in UK maternity care? (Yes = 5pts, No = 0pt)	Is the text a high, moderate or low match to the ARM@DA review questions/IPTs?	Does the text provide a high, moderate or low number of "nuggets" of information?	Total	Does the text provide a clear account of processes – ethics, sample, selection, limitations and biases noted?	Does the text include a clear description of analytical processes?	Does the text present a developed and plausible explanation?	Total	PRIORITY SCORE	Does the text offer a rich description, grounded in the data, of the process and context that can lead to explanatory insights?
Appelman, I. F., Thompson, S. M., van den Berg, L. M. M., Gitsels van der Wal, J. T., de Jonge, A. & Hollander, M. H. (2022) It was tough, but necessary. Organizational changes in a community based maternity care system during the first wave of the COVID-19 pandemic: A qualitative analysis in the Netherlands. PloS one, 17 (3 March): e0264311.	HS	0	5	3	8	5	5	5	15	3rd	3
Aydin, E., Glasgow, K. A., Weiss, S. M., Austin, T., Johnson, M., Barlow, J. & Lloyd-Fox, S. (2021) Expectant parents' perceptions of healthcare and support during COVID-19 in the UK: A thematic analysis. medRxiv, 2021.2004.2014.21255490.	GC	5	5	3	13	3	5	3	11	2nd	3
Bailey, C. M., Newton, J. M. & Hall, H. G. (2019) Telephone triage in midwifery practice: A cross-sectional survey. International journal of nursing studies, 91 110-118.	GC	0	3	5	8	5	5	5	15	3rd	5
Baron, A. M., Ridgeway, J. L., Finnie, D. M., Stirn, S. L., Morris, M. A., Branda, M. E., Inselman, J. W. & Baker, C. A. (2018) Increasing the Connectivity and Autonomy of RNs with Low-Risk Obstetric Patients: Findings of a study exploring the use of a new prenatal care model. AJN American Journal of Nursing, 118 (1): 48-55.	GC	0	5	5	10	3	3	3	9	4th	3
Bidmead, E., Lie, M., Marshall, A., Robson, S. & Smith, V. J. (2020) Service user and staff acceptance of fetal ultrasound telemedicine. Digital health, 6 2055207620925929.	GC	5	3	3	11	5	5	5	15	3rd	5
