# Supplementary material for: Optimising digital clinical consultations in maternity care: a realist review and implementation principles
Source: BMJ Open. 2024 Nov 1;14(10):e079153. doi: 10.1136/bmjopen-2023-079153 (PMC11529580; doi:10.1136/bmjopen-2023-079153)
Supplement: online supplemental file 11 [file bmjopen-14-10-s011.pdf]

Supplemental File 11: Phase 3 Search Strategies

Database Search Strategies

Embase <1974 to 2023 Week 13>

1 (realist or (theory adj3 change) or logic model* or program logic or programme logic or causal model* or results chain* or intervention logic).mp. 11318
2 (safety or safetynet* or safeguard* or safe-guard* or near miss* or never event* or adverse event* or adverse outcome*).mp. 1780103
3 ((risk* or harm*) adj3 (prevent* or reduc*)).mp. 399867
4 2 or 3 2119026
5 1 and 4 766
6 remove duplicates from 5 742
7 limit 6 to embase 361

Ovid MEDLINE(R) ALL <1946 to April 03, 2023>

1 (realist or (theory adj3 change) or logic model* or program logic or programme logic or causal model* or results chain* or intervention logic).mp. 9214
2 (safety or safetynet* or safeguard* or safe-guard* or near miss* or never event* or adverse event* or adverse outcome*).mp. 904994
3 ((risk* or harm*) adj3 (prevent* or reduc*)).mp. 235815
4 2 or 3 1115450
5 1 and 4 584
6 remove duplicates from 5 580

APA PsycInfo <1806 to March Week 4 2023>

1 (realist or (theory adj3 change) or logic model* or program logic or programme logic or causal model* or results chain* or intervention logic).mp. 10413
2 (safety or safetynet* or safeguard* or safe-guard* or near miss* or never event* or adverse event* or adverse outcome*).mp. 104683
3 ((risk* or harm*) adj3 (prevent* or reduc*)).mp. 44045
4 2 or 3 145102
5 1 and 4 329
6 remove duplicates from 5 329

CINAHL search (via EBSCO host)

#	Query	Limiters/Expanders	Last Run Via
S3	S1 AND S2	Expanders - Apply equivalent subjects<br>Search modes - Boolean/Phrase	Interface - EBSCOhost Research Databases<br>Search Screen - Advanced Search<br>Database - CINAHL
S2	((safety or safetynet* or safeguard* or safe-guard*)) OR ("near miss*" or "never event*" or "adverse event*" or "adverse outcome*") OR ((risk or harm) n3 (prevent* or reduc*))	Search modes - Boolean/Phrase	Interface - EBSCOhost Research Databases<br>Search Screen - Advanced Search<br>Database - CINAHL
S1	realist* OR theory n3 change OR ("logic model*" or "program logic" or "programme logic" or "causal model*" or "results chain*" or "intervention logic")	Search modes - Boolean/Phrase	Interface - EBSCOhost Research Databases<br>Search Screen - Advanced Search<br>Database - CINAHL

TOTAL RESULTS = 2662
Minus duplicates = 1998
Minus dissertations (n=81)

Focused Search Strategies: CLUSTER Approaches (Phase 3)

Topic Focus/Search Approach	Date	Records Found for Screening<br>• **Reports from Websites n=5**<br>• **Snowball/CLUSTER n=16**
**Safety/Risk in Remote Maternity Care**		
Keyword searches in Google and Google Scholar (combinations of safety/safety-netting/safe-guarding/risk and remote/virtual/telehealth/triage and maternity/midwifery/obstetrics) – in incognito mode, scrutinising the first 100 records<br><br>Reference list searching of identified records and citation searching	15/02/23	n=5
Keyword searches of records in existing reference management system<br><br>Reference list searching of records and citation searching	20/02/23	n=1
**Inequality/Access/Inclusion in Remote Maternity Care**		
Keyword searches in Google and Google Scholar (combinations of inequality/inclusion/access and remote/virtual/telehealth/triage and maternity/midwifery/obstetrics) – in incognito mode, scrutinising the first 100 records<br><br>Reference list searching of records and citation searching	16/02/23	n=1
Keyword searches of records in existing reference management system	20/02/23	No relevant records
**Both Maternity IPT Areas**		
**Key Author…..**<br>• **Citation Alerts**<br>• **New Publication Alerts**<br>• **Webpage search**<br><br>Lisa Hinton	Cut-off April 30th 2023	No relevant records

**Key/Pearl Paper Citation Alerts**<br><br>Hinton, L., Dakin, F. H., Kuberska, K., Boydell, N., Willars, J., Draycott, T., Winter, C., Mcmanus, R. J., Chappell, L. C., Chakrabarti, S., Howland, E., George, J., Leach, B. & Dixon-Woods, M. 2022. Quality framework for remote antenatal care: qualitative study with women, healthcare professionals and system-level stakeholders. *BMJ Quality & Safety,* 12**,** 12.	Cut-off April 30th 2023	n=1
**Related (Non-Maternity) Evidence on Safety/Risk/Inequality in Remote Consultations**		
**Key Paper Citation Alerts**<br><br>Greenhalgh, T., Rosen, R., Shaw, S. E., Byng, R., Faulkner, S., Finlay, T., Grundy, E., Husain, L., Hughes, G., Leone, C., Moore, L., Papoutsi, C., Pope, C., Rybczynska-Bunt, S., Rushforth, A., Wherton, J., Wieringa, S. & Wood, G. W. 2021. Planning and Evaluating Remote Consultation Services: A New Conceptual Framework Incorporating Complexity and Practical Ethics. *Front Digit Health,* 3**,** 726095.[37]	Cut-off April 30th 2023	**Inequality/Access/Inclusion**<br>n=5<br><br>**Safety/Risk**<br>n=5
**Key Author……**<br>• **Citation Alerts**<br>• **New Publication Alerts**<br>• **Webpage search**<br><br>Tricia Greenhalgh<br><br>Reference list searching of records and citation searching	Cut-off April 30th 2023	
**Keyword Searching in Existing Reference Management Programme**<br><br>Reference list searching of records and citation searching		n=3
