# Supplementary material for: Optimising digital clinical consultations in maternity care: a realist review and implementation principles
Source: BMJ Open. 2024 Nov 1;14(10):e079153. doi: 10.1136/bmjopen-2023-079153 (PMC11529580; doi:10.1136/bmjopen-2023-079153)
Supplement: online supplemental file 12 [file bmjopen-14-10-s012.pdf]

SUPPLEMENTAL FILE 12: CHARACTERISTICS OF INCLUDED STUDIES, REVIEWS AND REPORTS

**Key**
**Pandemic timing**:<br>During pandemic = DP, Pre-pandemic= PP
**Country**:<br>AUS = Australia, CAN: Canada, ESP = Spain, FRA = France, GER = Germany, IT = Italy, NLD = Netherlands, NZL = New Zealand, SUI = Switzerland, UK = United Kingdom, USA = United States of America
**Population:**<br>HCP = Healthcare professionals, MW = Midwives, RN = Registered nurses, OB = Obstetricians, SU = Service users, ADMIN = Administrative staff
**Stage of Pregnancy:**<br>AN = Antenatal, PN = Postnatal, IP = Intrapartum, T = Triage, n/a = not applicable

Characteristics: Empirical Papers

Reference	Pandemic timing	Geographical focus	Population	Stage of pregnancy	Tech modality	Study aim	Study design	Outcomes
Appelman et al. 2022[1]	DP	NLD	HCP	n/a	Telephone and video calls	To investigate which policy changes in maternity care during the first COVID-19 wave were perceived as positive or unfavourable by care providers and that could offer future improvements.	Qualitative	Experiences of HCPs, policy changes, cooperation between HCPs, practices and hospitals.
Aydin et al. 2021[2]	DP	UK	SU	AN	Telephone and video calls	To examine how COVID-19 and its societal related restrictions have impacted the provision of healthcare support for pregnant women during the COVID-19 pandemic.	Quantitative; cross-sectional	Changes to service provision linked to SU anxiety levels.

Reference	Pandemic timing	Geographical focus	Population	Stage of pregnancy	Tech modality	Study aim	Study design	Outcomes
Bailey et al. 2019[3]	PP	AUS	MW	T	Telephone calls	To explore the experiences and practices of midwives regarding their management of telephone triage.	Quantitative; cross-sectional	MW experiences and practices.
Baron et al. 2018[4]	PP	USA	SU, HCP	AN	Telephone and video calls	To explore the perspectives of patients, RNs, and other providers regarding a new prenatal connected care model for low-risk patients aimed at reducing in-office visits and creating virtual patient–RN connections.	Qualitative	Satisfaction, appointment type/number.
Bidmead et al. 2020[5]	PP	UK	SU, HCP	AN	Video calls	For women, to directly assess experiences and acceptance of fetal telemedicine. For HCPs, to identify the barriers and enablers of adoption of fetal telemedicine.	Mixed methods	SU and HCP acceptance and satisfaction with fetal ultrasound telemedicine.
Borrelli et al. 2023[6]	DP	UK/IT	SU	IP; T	Video calls	To report on mothers' perspectives on the potential use of video-calls during early labour in England and Italy.	Qualitative	Implementation benefits and barriers to video calling in early labour.
Borrelli et al. 2023[7]	DP	UK/IT	MW	IP; T	Video calls	To explore midwives' perspectives on potential use of video-	Qualitative	MW perspectives, satisfaction, challenges, best-practice.

Reference	Pandemic timing	Geographical focus	Population	Stage of pregnancy	Tech modality	Study aim	Study design	Outcomes
						calls during early labour.		
Branwer et al. 2021[8]	DP	UK	SU	AN; PN	Telephone calls	To rapidly gather data on the health, social, education and economic impacts of the COVID-19 pandemic on families in Bradford, UK.	Qualitative	SU experiences. Recommendations for service providers.
Butler Tobah et al. 2019[9]	PP	USA	SU	AN	Multiple technologies, including telephone calls, video calls and remote monitoring.	To evaluate the acceptability and effectiveness of OB Nest, a reduced-frequency prenatal care model enhanced with remote home monitoring devices and nursing support.	RCT	Acceptability, satisfaction, effectiveness prenatal maternal stress.
Cordasco et al. 2018[10]	PP	USA	SU	AN; PN	Telephone calls	To develop and assess feasibility, as well as facilitators and barriers, of implementing the VA Maternity Care Coordinator Telephone Care Program.	Mixed methods	Feasibility of telephone care.
Craighead et al. 2022[11]	DP	USA	SU	AN	Not specified	To understand the impact of telehealth on healthcare communication and quality, and patient satisfaction	Mixed methods	Understanding the challenges of implementing telehealth for prenatal care delivery during the pandemic.
Demirci et al. 2019[12]	PP	USA	SU	PN	Video calls via an App.	To describes the feasibility and acceptability of direct	Qualitative	Feasibility of tele-lactation.

Reference	Pandemic timing	Geographical focus	Population	Stage of pregnancy	Tech modality	Study aim	Study design	Outcomes
						to consumer telelactation for rural mothers.		
Duryea et al. 2021[13]	DP	USA	SU	AN	Telephone calls	To explore the association of audio-only virtual prenatal care with perinatal outcomes.	Quantitative; cohort study	Clinical outcomes.
Engeltjes et al. 2022[14]	PP	NLD	HCP	Unclear	Telephone calls	To evaluate the degree of implementation (i.e., normalization) of the Dutch Obstetric Telephone Triage System (DOTTS) and evaluate which lessons can be learned from its current implementation in Dutch hospitals.	Mixed methods	Implementation (i.e. normalisation) of DOTTS.
Engeltjes et al. 2023[15]	PP	NLD	SUs	AN; T	Telephone calls	To explore how care is experienced by pregnant women when using a telephone obstetric triage system.	Qualitative	SU experiences, satisfaction.
Engeltjes et al. 2020[16]	PP	NLD	HCPs	AN; T	Telephone calls	To develop obstetric guidelines for telephonic triage.	Mixed methods	HCPs views on guidelines.
Evans et al. 2017[17]	PP	USA	SU, RN	AN; PN	Telephone calls	To characterize nursing care delivered via telephone social support intervention to low-income, pregnant women in the Midwestern USA.	Qualitative	Feasibility of 'tele-nursing', improvement of clinical and psychosocial outcomes.
Farrell et al. 2022[18]	DP	USA	SU	AN	Unclear	To examine patients' prenatal care needs, preferences, and	Qualitative	SU satisfaction and care preferences during COVID-19.

Reference	Pandemic timing	Geographical focus	Population	Stage of pregnancy	Tech modality	Study aim	Study design	Outcomes
						experiences during the COVID-19 pandemic; to develop models to serve the needs of pregnant patients, providers, and healthcare systems.		
Faucher and Kennedy. 2020[19]	PP	USA	SU	IP; T	Video calls	To examine women's perspectives on the potential use of video technology for early labour support.	Qualitative	SU satisfaction.
Fernandez Lopez et al. 2022[20]	DP	ESP	SU	AN	Video calls	To identify the needs, concerns and preferences of survivors about the use of eHealth strategies to counsel and empower pregnant victims of intimate partner violence in antenatal care.	Qualitative	Suitability of eHealth for pregnant survivors of intimate partner violence.
Foster et al. 2022[21]	DP	USA	HCP	AN	Unclear	To identify mean differences in telehealth maternity care; perceived patient acceptability; clinician satisfaction; and the perceived anticipation of long-term telehealth utilization in family medicine maternity care.	Quantitative; cross-sectional	Acceptability and satisfaction.
Galle et al. 2021[22]	DP	Global	HCP	AN; IP; PN	Telephone and video, SMS	To document the experiences with providing telemedicine	Quantitative; cross-sectional	Implementation of telemedicine, barriers to

Reference	Pandemic timing	Geographical focus	Population	Stage of pregnancy	Tech modality	Study aim	Study design	Outcomes
						for maternal and newborn healthcare during the pandemic among health professionals globally.		effectiveness, HCP perceptions and experiences.
Gao et al. 2022[23]	DP	USA	SU	AN	Not specified	To investigate which prenatal visits are appropriate to be replaced with telehealth, access barriers and how telehealth impacts maternal outcomes.	Quantitative; observational	Telehealth use, disparities in SU using telehealth, clinical outcomes.
Gemperle et al. 2022[24]	DP	SUI	MW	AN; PN	Multiple technologies	To explore midwives' perceptions of the advantages of telemedicine during the COVID-19 pandemic in Switzerland.	Quantitative; cross-sectional	Perceptions of telemedicine
Gomez-Roas et al. 2022[25]	DP	USA	SU	PN	Telephone and video calls	To identify additional challenges to healthcare interactions that emerged for low-income postpartum individuals during the pandemic.	Qualitative	Equity and access to care.
Harrison et al. 2017.[26]	PP	USA	SU	AN	Telephone and video calls	To assess the acceptability of a telemedicine-augmented gestational diabetes mellitus management protocol, which alternates "virtual office visits"	Mixed methods	Acceptability of telemedicine for gestational diabetes mellitus care.

Reference	Pandemic timing	Geographical focus	Population	Stage of pregnancy	Tech modality	Study aim	Study design	Outcomes
						and standard office-based prenatal visits.		
Henry et al. 2022[27]	DP	AUS	HCP	AN; PN	Telephone and video calls	To assess COVID-19 effects on domestic and family violence and mental health screening, as well as broader service provision from the perspective of local maternity service providers.	Mixed methods	Suitability of telehealth for assessing domestic and family violence and mental health. Broader advantages and disadvantages of telehealth.
Hinton et al. 2022[28]	DP	UK	SU, HCP	AN	Telephone and video calls	To characterise what quality remote antenatal care looks like from the perspectives of those who use, provide and organise it.	Mixed methods	Service improvement, quality.
Hinton et al. 2023[29]	DP	UK	SU, HCP	AN	Telephone and video calls	To explore the experiences and perspectives of pregnant women, antenatal healthcare professionals, and system leaders to understand the impact of implementing remote antenatal care during COVID-19 and beyond.	Qualitative	Access, equity, experiences, use of 'candidacy' to understand access to remote antenatal care.
Jeganathan et al. 2020[30]	DP	USA	SU, HCP	AN	Telephone and video calls	To describe patient and provider attitudes toward telehealth for the delivery of high-risk obstetrical care and to	Quantitative; cross-sectional	Attitudes and feasibility; reduction in no-show rates and cancellations.

Reference	Pandemic timing	Geographical focus	Population	Stage of pregnancy	Tech modality	Study aim	Study design	Outcomes
						determine whether the implementation of a telehealth model improves patient adherence to scheduled appointments.		
Karavadra et al. 2020[31]	DP	UK	SU	All	Not specified	To explore pregnant women's perceptions of COVID-19 and their healthcare experiences. To obtain insight into any barriers to healthcare during this pandemic and any concerns women have about their pregnancy.	Quantitative; cross-sectional	Attitudes and feasibility.
Khalil. 2019[32]	PP	FRA	SU, HCP	AN	Remote monitoring (myDiabby app) and telephone calls	To understand, from patients' and HCPs' perspective, what drives the adoption and diffusion of myDiabby (telemonitoring platform) in healthcare centres where telemonitoring of women with gestational diabetes mellitus is not compensated.	Qualitative	Satisfaction with care and understanding of factors that influence diffusion and adoption.
Khosla et al. 2022[33]	DP	USA	SU	PN	Telephone calls	To investigate whether rapid switch to telehealth with audio-based visits during the	Quantitative; retrospective cohort study	Adherence to postpartum hypertension follow

Reference	Pandemic timing	Geographical focus	Population	Stage of pregnancy	Tech modality	Study aim	Study design	Outcomes
						COVID-19 pandemic decreased racial disparities in postpartum hypertension follow-up adherence.		up. Readmission rates.
Klamroth-Marganska et al. 2021[34]	DP	SUI	HCP	n/a	Telephone and video calls	To identify the use of services and to appraise the experiences of HCPs regarding the provision of healthcare at a distance during lockdown. To understand facilitators and barriers for successful implementation of tele-health applications.	Quantitative; cross-sectional	Usage, satisfaction, concerns, support needs.
Kluwgant et al. 2022[35]	DP	AUS	SU	AN; PN	Not specified	To understand the positive aspects of the changes to antenatal and childbirth care from COVID-19 from the perspectives of both pregnant women and midwives.	Quantitative; cross-sectional	Positive impacts, care-related factors and contextual factors.
Kozica-Olenski et al. 2022[36]	DP	AUS	SU, HCP	AN	Telephone and video calls	To explore the experiences and acceptability of telehealth for general maternity care and in diabetes pregnancy care during the COVID-19 pandemic, from the perspectives	Qualitative	Satisfaction, benefits, barriers, evaluation against the NASSS framework.

Reference	Pandemic timing	Geographical focus	Population	Stage of pregnancy	Tech modality	Study aim	Study design	Outcomes
						of pregnant women and their clinicians.		
Krenitsky et al. 2020[37]	DP	USA	SU	AN; PN	Telephone and video calls (integrated with remote monitoring)	To describe the experience of an academic institution and its community hospital partner in establishing a virtual clinic for obstetric patients with mild or resolving acute COVID-19 infections, including the process, challenges, outcomes and lessons.	Quantitative; observational	Clinical outcomes, rates of follow up.
Lapadula et al. 2021[38]	DP	USA	SU, HCP	AN	Video calls (Zoom)	To evaluate patients' and neonatologists' satisfaction with virtual prenatal consultations and to compare satisfaction levels of patients receiving virtual consultation with those receiving in-person care.	Quantitative; cross-sectional	Satisfaction.
Leighton et al. 2019[39]	PP	USA	SU	AN; PN	Video calls (supported by tele-ultrasound)	To compare maternal and child health outcomes between telemedicine care and traditional in-person care. To calculate the time and resources saved by using a telemedicine approach.	Quantitative; observational	Satisfaction, patient and service-related cost savings, clinical outcomes.

Reference	Pandemic timing	Geographical focus	Population	Stage of pregnancy	Tech modality	Study aim	Study design	Outcomes
Liu et al. 2021[40]	DP	USA	SU	AN	Not specified	To identify factors related to satisfaction with virtual visits during pregnancy in an effort to prioritize intervention targets for pregnant women during the COVID-19 pandemic	Quantitative; cross-sectional	Satisfaction and preferences.
Madden et al. 2020[41]	DP	USA	HCP	AN; PN	Video calls	To determine to what degree prenatal care was able to be transitioned to telehealth at prenatal practices associated with two affiliated hospitals in New York City, USA, during the COVID-19 pandemic and describe providers' experiences.	Mixed methods	Satisfaction, barriers, facilitators.
Mann et al. 2021[42]	DP	USA	SU	AN	Not specified	To increase knowledge and understanding of telehealth for reproductive genetic counselling services.	Quantitative; observational	SU access to genetic counselling and services.
Mehl et al. 2022[43]	DP	USA	SU	AN; PN	Video calls	To explore differences in demographics of expectant mothers evaluated pre- and post-telemedicine implementation, and the patient experience with telemedicine.	Mixed methods	Distance and travel time, patient demographics.
Moltrecht et al. 2022[44]	DP	UK	SU	AN; PN	Not specified	To explore young parents' experiences	Qualitative	Experiences of care.

Reference	Pandemic timing	Geographical focus	Population	Stage of pregnancy	Tech modality	Study aim	Study design	Outcomes
						and perceptions of becoming and being parents during the COVID-19 pandemic.		
Moltrecht et al. 2022[45]	DP	UK	HCP	AN; PN	Not specified (various modalities)	To explore HCP experiences of providing care to young parents during the COVID-19 pandemic.	Qualitative	Pandemic-related changes to services.
Morgan et al. 2022[46]	DP	USA	SU	AN	Telephone and video calls	To evaluate patient experience with a prenatal telemedicine visit and identify barriers to accessing telemedicine among rural pregnant people in New England, USA, during COVID-19.	Quantitative; cross-sectional	Satisfaction.
Nelson and Holschuh. 2021[47]	PP	USA	SU, HCP	AN	Not specified	To evaluate a new hybrid antenatal model of care in which some in-person visits were replaced by tele-consults.	Quantitative; cross-sectional	Satisfaction.
Oelmeier et al. 2022[48]	DP	GER	SU, HCP	AN; PN	Video calls	To evaluate the technical feasibility and patient satisfaction with video consultations in a tertiary centre for obstetric care.	Quantitative; cross-sectional	Satisfaction, acceptability
Osarhiemen et al. 2022[49]	DP	USA	SU	AN; PN	Unclear	To define vulnerable obstetrical populations that were more likely to miss scheduled visits	Quantitative; observational	No-show rate, access to care

Reference	Pandemic timing	Geographical focus	Population	Stage of pregnancy	Tech modality	Study aim	Study design	Outcomes
						before the COVID-19 pandemic and to quantify the impact of telehealth on the odds of no-shows in vulnerable obstetrical populations.		
Palmer et al. 2021[50]	DP	AUS	SU	AN	Video calls (95%) and telephone calls (5%)	To assess the impact of telehealth integration into antenatal care across low-risk and high-risk care models.	Quantitative; interrupted time series analysis	Safety, efficacy, clinical outcomes.
Peahl et al. 2021[51]	DP	USA	SU, HCP	AN	Telephone and video calls	To evaluate initial adoption and patient and provider care experience of a hybrid (integrated antenatal care model).	Quantitative service evaluation; observational	Adoption, adherence, satisfaction.
Pflugeisen et al. 2016[52]	PP	USA	SU	AN; PN	Video calls (and digital BP machine & fetal doppler monitor)	To evaluate a new hybrid model of pre- and post-natal care in which women are offered a choice of in-person consultations or a hybrid programme with some video consultations.	Quasi-experimental	Safety, clinical outcomes, health service outcomes, hospital admissions, emergency department attendance rates.
Pflugeisen and Mou. 2017[53]	PP	USA	SU	AN; PN	Video calls (and digital BP machine & fetal doppler monitor)	To compare the satisfaction of obstetric patients who received one-third of their antenatal visits in videoconference compared to those who received 12–14	Quasi-experimental	Satisfaction.

Reference	Pandemic timing	Geographical focus	Population	Stage of pregnancy	Tech modality	Study aim	Study design	Outcomes
						face-to-face visits in clinic with their physician/midwife.		
Quinn et al. 2021[54]	DP	UK	SU, HCP, ADMIN,	AN	Telephone calls	To evaluate patient and healthcare professional satisfaction, preferences, and experiences of a virtual antenatal clinic during the COVID-19 pandemic from a tertiary obstetric hospital.	Quantitative; cross sectional; service evaluation	Satisfaction.
Rasekaba et al. 2021[55]	PP	AUS	SU, HCP, ADMIN,	AN	Not specified	To identify the profiles of women accessing care for gestational diabetes mellitus in a large regional hospital with a rural catchment and the views of women, clinicians and IT staff on the acceptability and feasibility of telehealth in this context.	Mixed methods	Feasibility, satisfaction, burden of treatment.
Rayment-Jones et al. 2023[56]	PP	UK	SU	AN	Telephone call, text message or free technology (freephone number, WhatsApp etc)	To evaluate how women access and engage with different models of maternity care, whether specialist models improve access and engagement for women with social risk factors, and if so, how?	Quantitative; observational (with mixed methods)	Access, engagement, equity.

Reference	Pandemic timing	Geographical focus	Population	Stage of pregnancy	Tech modality	Study aim	Study design	Outcomes
Rayment-Jones et al. 2022[57]	PP	UK	SU	AN	Telephone call, text message or free technology (freephone number, WhatsApp etc)	To evaluate two specialist models of care that provide continuity to women with social risk factors and identify mechanisms that reduce or exacerbate health inequalities.	Qualitative	Service evaluation, access, engagement, equity.
Reid et al. 2021[58]	DP	USA	OB	AN; PN	Telephone and video calls	To assess the rapid implementation of obstetric telemedicine during the COVID-19 pandemic.	Mixed methods	Feasibility, satisfaction
Rousseau et al. 2022.[59]	DP	FRA	MW	n/a	Telephone and video calls	To measure and understand the determinants of independent midwives' implementation of teleconsultations and their intention to continue these in the future.	Mixed methods	Implementation of telehealth, intention to continue telehealth, and explanation of these two variables.
Saad et al. 2021.[60]	DP	CAN	SU	PN	Video calls	To understand the perspectives of new mothers using virtual visits. To understand the barriers and facilitators.	Qualitative	Access, satisfaction, financial benefits.
Sanders and Blaylock. 2021[61]	DP	UK	SU	AN; PN	Telephone and video calls	To understand the impact of COVID-19 public health messaging and pandemic-related service changes on users of maternity care	Mixed methods	Messaging, access, satisfaction.

Reference	Pandemic timing	Geographical focus	Population	Stage of pregnancy	Tech modality	Study aim	Study design	Outcomes
						in the UK during the pandemic.		
Sarre et al. 2021[62]	DP	UK	SU	AN	Telephone and video calls	To explore patients' experience of antenatal diabetic maternity services during the current COVID-19 pandemic.	Mixed methods	Satisfaction.
Shashikumar et al. 2022[63]	DP	NZL	SU	AN	Telephone calls	To determine satisfaction of pregnant people with teleclinics for diabetes in pregnancy; compare clinical outcomes and attendance for those receiving care through teleclinics versus standard care.	Quantitative; cross sectional	Satisfaction and future use of telehealth, clinical outcomes, number of appointments.
Shaw et al. 2018[64]	PP	UK	SU	AN	Video calls	To define good practice and inform digital technology implementation in relation to remote consultations via Skype and similar technologies.	Mixed methods	Satisfaction, efficiency, best practice.
Silverio et al. 2021[65]	DP	UK	SU	AN; PN	Telephone and video calls	To explore women's experiences of maternity service reconfiguration during the first wave of the COVID-19 pandemic.	Qualitative	Women's experiences, satisfaction.
Smith et al. 2020[66]	DP	UK	SU	AN; PN	Telephone and video calls	To understand the impact of the changes that were introduced in the first period of	Mixed methods	Satisfaction.

Reference	Pandemic timing	Geographical focus	Population	Stage of pregnancy	Tech modality	Study aim	Study design	Outcomes
						lockdown and local restrictions (March 2020-August 2020) on expectant and new parents and families.		
Smith et al. 2021[67]	PP	UK	SU	AN	Video calls	To report the successful introduction of a fetal ultrasound telemedicine service linking a specialist fetal medicine centre and a remote obstetric unit.	Mixed methods	Satisfaction, clinical, time/cost savings for women.
Spiby et al. 2019[68]	PP	UK/USA	MW	IP	Video calls	To explore midwives' views on the potential of video-calling as a method for assessing women in early labour.	Qualitative	Midwives views
Stacey et al. 2021[69]	DP	UK	SU	AN; PN	Telephone and video calls	To explore service users' and their partners' experiences of maternity services in the North of England during the COVID-19 pandemic.	Mixed methods	Experiences of care during COVID-19.
Sullivan et al. 2021[70]	DP	USA	SU	AN	Telephone and video calls	To determine acceptability of virtual prenatal care and preferences for future pregnancies among our patient population.	Quantitative; cross sectional	SU acceptability and preferences of virtual prenatal care
Sung et al. 2021[71]	DP	USA	SU	AN; PN	Unclear	To evaluate the effects of the High-Risk Pregnancy (telemedicine) Program at the University of Arkansas,	Quantitative; observational	Admissions, insulin usage, cost/expenditure, clinical outcomes, number of visits.

Reference	Pandemic timing	Geographical focus	Population	Stage of pregnancy	Tech modality	Study aim	Study design	Outcomes
						USA, on health services utilisation and medical expenditures among pregnant women with pre-existing diabetes and their newborns.		
Talmont et al. 2022[72]	DP	USA	RN	AN; PN	Telephone and video calls	To assess telehealth readiness among perinatal nurses in New Jersey, USA.	Quantitative; cross sectional	Telehealth readiness, usage and acceptability.
Tavener et al. 2022.[73]	DP	UK	SU, OB, MW	AN	Telephone and video calls	To introduce telephone consultations to reduce need to attend the clinic and to reduce waiting times for those women needing to be seen face-to-face.	Quality Improvement Initiative	Waiting times in clinic, patient and staff satisfaction.
Theiler et al. 2021.[74]	PP	USA	SU	AN	Multiple technologies, including telephone calls, video calls and remote monitoring.	To explore the cost implications of telemedicine-enhanced programmes added to prenatal care packages.	RCT	Appointment time, cost.
Tozour et al. 2021[75]	DP	USA	SU	Unclear	Telephone and video calls	To evaluate both patient and provider satisfaction with maternal-fetal medicine services through telemedicine and to identify the factors that drive the patient desire for future	Quantitative; cross-sectional	Satisfaction.

Reference	Pandemic timing	Geographical focus	Population	Stage of pregnancy	Tech modality	Study aim	Study design	Outcomes
						obstetrical telemedicine.		
van den Heuvel et al. 2020[76]	PP	NLD	SU	AN	Remote monitoring and telephone calls.	To explore the usability and acceptability of telemonitoring and gain insight into the experiences and preferences of high risk pregnant women concerning telemonitoring, opposed to women who were hospitalised in pregnancy.	Qualitative	Feasibility, useability, acceptability, experiences, preferences.
Zulifqar. 2021[77]	DP	USA	HCP	AN	Telephone and video calls	To understand provider satisfaction with providing prenatal care in various formats.	Quantitative; cross sectional	Provider satisfaction.

Characteristics: Reviews

Reference	Geographical focus	Stage of pathway	Tech modality	Study aim	Review methodology	Number of studies included	Range of studies included
Almuslim et al. 2022[78]	Not stated (appears global)	AN; PN	Not stated	To determine how healthcare organizations are responding to the COVID-19 pandemic by incorporating telehealth visits into their protocols for obstetric care, what services were converted to telehealth, and its benefits and barriers.	Scoping review	15 clinical practice protocols; 10 studies.	All 2020
Bailey et al. 2018[79]	Global (all USA or UK)	T	Telephone calls	To identify and determine the nature and degree of literature on midwives' practice of telephone triage to inform future educational strategies and	Scoping review	11	1999-2014

Reference	Geographical focus	Stage of pathway	Tech modality	Study aim	Review methodology	Number of studies included	Range of studies included
				practice, and to identify gaps in the literature to guide future research.			
Cantor et al. 2022[80]	High income countries	AN; PN	Multiple modalities including telephone calls, video calls and Apps.	To conduct a rapid review of the effectiveness and harms of telehealth strategies for maternal healthcare given the recent expansion of telehealth from the COVID-19 pandemic; produce an evidence map.	Rapid systematic review with narrative summary	42 studies (45 publications)	2015-2022
Chua et al. 2022[81]	High income countries	PN	Video calls.	To consolidate and synthesize findings on the available evidence of tele-lactation interventions on breastfeeding outcomes, uptake of interventions, and recommendations for future lactation interventions	Mixed studies systematic review	13	2007-2021
Fernandez Turienzo et al. 2021[82]	High income countries (all AUS, UK, USA)	AN	Telephone calls	To uncover theories of change by which we can postulate how and why continuity of midwifery care models might affect preterm birth.	Realist review	11	1996-2017
Flaherty et al. 2022[83]	Global	AN; PN	Not stated	To gain insight and understanding of the experience of maternity care during COVID-19, from the perspectives of women and maternity care providers.	Qualitative systematic review using thematic synthesis	48 studies (50 papers)	2020-2021
Friedemann Smith et al. 2022[84]	Global	n/a	Telephone calls	To produce a programme theory of safety-netting, that is, advice and support provided to patients when diagnosis or prognosis is uncertain, in primary care.	Realist review	95	1996-2021
Ghimire et al. 2023[85]	High income countries	AN	Telephone and video calls	To assess the practical implications of virtual prenatal care and identify the needs and experiences associated with it.	Systematic review (mixed methods) using integrative analysis	23	2011-2021

Reference	Geographical focus	Stage of pathway	Tech modality	Study aim	Review methodology	Number of studies included	Range of studies included
Konnyu et al. 2023[86]	High income countries	AN	Telephone and video calls.	To systematically review patient, partner or family, and clinician perspectives, preferences, and experiences related to (i) prenatal care visit schedules and (ii) tele-visits for routine prenatal care.	Qualitative systematic review using framework analysis approach	9 (only 5 of which looked at telehealth)	1995-2022
Society for Maternal-Fetal Medicine. 2022[87]	Not stated	Any	Multiply modalities including telephone and video calls.	To summarize the literature regarding the safety and quality of telemedicine for pregnancy-related services, including prenatal care, postpartum care, diabetes mellitus management, medical abortion, lactation support, hypertension management, genetic counselling, ultrasound examination, contraception, and mental health.	Narrative review	Not stated	Not stated
Wu et al. 2021[88]	High income countries	AN	Telephone and video calls.	To gain a deeper understanding of (1) how virtual visits have been integrated with in-person visits during routine prenatal care and (2) how patients and healthcare providers have experienced combined virtual and in-person visits.	Systematic review (integrative approach)	13	2013-2020

Characteristics: Reports

Reference	Geographical focus	Report aim	Methodology	Reporting period
Healthcare Safety Investigation Branch. 2021[89]	England	To inform understanding about the range of factors that may have contributed to the increased referral rate to HSIB of incidences of intrapartum stillbirth; promote and support learning discussions within organisations; influence the development of systems and processes to optimise patient safety; identify potential safety risks that merit further HSIB investigation.	Review of 37 reports concerning cares of intrapartum still birth that occurred during the time period.	01/04/20 – 30/06/20

Reference	Geographical focus	Report aim	Methodology	Reporting period
Healthcare Safety Investigation Branch. 2021[90]	England	To investigate maternal deaths during the first peak of the COVID-19 pandemic; inform understanding about the range of factors that contributed to harm at a local, regional, and national level; support learning discussions within organisations; influence the development of systems and processes to optimise patient safety; identify potential safety risks that merit further HSIB investigation.	Review of 19 maternal deaths that happened in England during the time period (out of 20).	01/03/20 – 31/05/20
Healthcare Safety Investigation Branch. 2023[91]	England	This national learning report analyses themes from HSIB's maternity investigation programme in relation to the risk assessment of pregnant women/people, with the aim of identifying key learnings about risk assessment.	Thematic review of 208 reports of maternity investigations that had made a total of 271 findings and recommendations to NHS trusts about risk assessment across the entire maternity pathway, including the antenatal and intrapartum periods.	April 2019 – January 2022
Knight et al. 2021[92]	UK	To respond to the second wave of COVID-19 in the UK which brought further challenges to maternity services and a higher burden of infection, together with new variants of concern. The aim was to ensure any new messages for care and services were identified in a timely manner in order to implement rapid change.	The care of 17 women was assessed by 5-7 multi-disciplinary review experts.	June 2020 – March 2021
Knight et al. 2022[93]	UK (some data included from Ireland)	Confidential enquiry into maternal deaths and morbidity for women who died during or up to one year after pregnancy (and focus on morbidity in relation to diabetic ketoacidosis). Focus on women who died from cardiovascular causes, hypertensive disorders, early pregnancy disorders and accidents, and mental-health related causes.	Epidemiological surveillance information for 536 women who died and 61 women who suffered with diabetic ketoacidosis. For each death, care was examined by 10-15 multi-disciplinary review experts and assessed against current guidelines and standards	2018 – 2020

References

1. Appelman IF, Thompson SM, van den Berg LMM, et al. It was tough, but necessary. Organizational changes in a community based maternity care system during the first wave of the COVID-19 pandemic: A qualitative analysis in the Netherlands. *PLOS One* 2022; 17: e0264311. DOI: https://dx.doi.org/10.1371/journal.pone.0264311.
2. Aydin E, Glasgow KA, Weiss SM, et al. Expectant parents' perceptions of healthcare and support during COVID-19 in the UK: A thematic analysis. *medRxiv* 2021. DOI: 10.1101/2021.04.14.21255490.
3. Bailey CM, Newton JM and Hall HG. Telephone triage in midwifery practice: A cross-sectional survey. *International Journal of Nursing Studies* 2019; 91: 110-118. DOI: 10.1016/j.ijnurstu.2018.11.009.
4. Baron AM, Ridgeway JL, Finnie DM, et al. Increasing the Connectivity and Autonomy of RNs with Low-Risk Obstetric Patients: Findings of a study exploring the use of a new prenatal care model. *Am J Nurs* 2018; 118: 48-55. DOI: 10.1097/01.NAJ.0000529715.93343.b0.
5. Bidmead E, Lie M, Marshall A, et al. Service user and staff acceptance of fetal ultrasound telemedicine. *Digit Health* 2020; 6: 2055207620925929. DOI: https://dx.doi.org/10.1177/2055207620925929.
6. Borrelli S, Downey, J., Fumagalli, S., Colciago, E., Antonella, N., Spiby, H. How should a video-call service for early labour be provided? A qualitative study of midwives' perspectives in the United Kingdom and Italy. *Women Birth* 2023. DOI: https://doi.org/10.1016/j.wombi.2023.06.00.
7. Borrelli S, Downey J, Colciago E, et al. Mothers' perspectives on the potential use of video-calling during early labour in the United Kingdom and Italy: A qualitative study. *Women Birth* 2023. DOI: https://doi.org/10.1016/j.wombi.2023.01.004.
8. Branwer JGR, D; Jackson, C; Dickerson, J; Dharni, N; Sheard, L; Smith, H; . *"What if I'm on my own?" Interim Report: Experiences of Pregnancy and Birth During the COVID-19 Pandemic*. 2021. Bradford Research https://www.bradfordresearch.nhs.uk/wp-content/uploads/2021/05/BiB-Qualitative-study_Pregnancy-in-COVID_brief-report_FINAL.pdf [accessed 07/07/22]
9. Butler Tobah YS, LeBlanc A, Branda ME, et al. Randomized comparison of a reduced-visit prenatal care model enhanced with remote monitoring. *Am J Obstet Gynecol* 2019; 221: 638.e631-638.e638. DOI: 10.1016/j.ajog.2019.06.034.
10. Cordasco KM, Katzburg JR, Katon JG, et al. Care coordination for pregnant veterans: VA's Maternity Care Coordinator Telephone Care Program. *Translational Behavioral Medicine* 2018; 8: 419-428. DOI: https://dx.doi.org/10.1093/tbm/ibx081.
11. Craighead CG, Collart C, Frankel R, et al. Impact of Telehealth on the Delivery of Prenatal Care During the COVID-19 Pandemic: Mixed Methods Study of the Barriers and Opportunities to Improve Health Care Communication in Discussions About Pregnancy and Prenatal Genetic Testing. *JMIR Formative Research* 2022; 6: e38821. DOI: https://dx.doi.org/10.2196/38821.
12. Demirci J, Kotzias V, Bogen DL, et al. Telelactation via Mobile App: Perspectives of Rural Mothers, Their Care Providers, and Lactation Consultants. *Telemedicine Journal and E-health: The Official Journal of the American Telemedicine Association* 2019; 25: 853-858. DOI: https://dx.doi.org/10.1089/tmj.2018.0113.
13. Duryea EL, Adhikari EH, Ambia A, et al. Comparison Between In-Person and Audio-Only Virtual Prenatal Visits and Perinatal Outcomes. *JAMA Network Open* 2021; 4: e215854-e215854. DOI: 10.1001/jamanetworkopen.2021.5854.
14. Engeltjes B, Rosman A, Scheele F, et al. Evaluation of Normalization After Implementation of the Digital Dutch Obstetric Telephone Triage System: Mixed Methods Study With a Questionnaire Survey and Focus Group Discussion. *JMIR Formative Research* 2022; 6: e33709. DOI: 10.2196/33709.
15. Engeltjes B, van Herk N, Visser M, et al. Patients' experiences with an obstetric telephone triage system: A qualitative study. *Patient Education and Counseling* 2023; 108: 107610. DOI: https://doi.org/10.1016/j.pec.2022.107610.
16. Engeltjes B, Wouters E, Rijke R, et al. Obstetric telephone triage. *Risk Manage Healthc Policy* 2020; 13: 2497-2506. DOI: http://dx.doi.org/10.2147/RMHP.S277464.
17. Evans EC and Bullock LFC. Supporting Rural Women During Pregnancy: Baby BEEP Nurses. *MCN: The American Journal of Maternal Child Nursing* 2017; 42: 50-55. DOI: 10.1097/NMC.0000000000000305.
18. Farrell R, Collart C, Craighead C, et al. The Successes and Challenges of Implementing Telehealth for Diverse Patient Populations Requiring Prenatal Care During COVID-19: Qualitative Study. *JMIR Formative Research* 2022; 6: e32791. DOI: https://dx.doi.org/10.2196/32791.

19. Faucher MA and Kennedy HP. Women's Perceptions on the Use of Video Technology in Early Labor: Being Able to See. *Journal of Midwifery & Women's Health* 2020; 65: 342-348. DOI: https://dx.doi.org/10.1111/jmwh.13091.
20. Fernandez Lopez R, de-Leon-de-Leon S, Martin-de-Las-Heras S, et al. Women survivors of intimate partner violence talk about using e-health during pregnancy: a focus group study. *BMC Women's Health* 2022; 22: 98. DOI: https://dx.doi.org/10.1186/s12905-022-01669-2.
21. Foster KE, Casola AR, Uzumcu Z, et al. Outpatient maternity care and telemedicine use perceptions in the COVID-19 pandemic: a 2020 CERA survey. *Women & Health* 2022; 62: 402-411. DOI: https://dx.doi.org/10.1080/03630242.2022.2072051.
22. Galle A, Semaan A, Huysmans E, et al. A double-edged sword-telemedicine for maternal care during COVID-19: findings from a global mixed-methods study of healthcare providers. *BMJ Glob Health* 2021; 6. DOI: 10.1136/bmjgh-2020-004575.
23. Gao C, Osmundson S, Malin BA, et al. Telehealth Use in the COVID-19 Pandemic: A Retrospective Study of Prenatal Care. *Studies in Health Technology & Informatics* 2022; 290: 503-507. DOI: 10.3233/SHTI220127.
24. Gemperle M, Grylka-Baeschlin S, Klamroth-Marganska V, et al. Midwives' perception of advantages of health care at a distance during the COVID-19 pandemic in Switzerland. *Midwifery* 2022; 105: 103201. DOI: https://dx.doi.org/10.1016/j.midw.2021.103201.
25. Gomez-Roas MV, Davis KDM, Leziak K, et al. Postpartum during a pandemic: Challenges of low-income individuals with healthcare interactions during COVID-19. *PLOS One* 2022; 17: e0268698. DOI: https://dx.doi.org/10.1371/journal.pone.0268698.
26. Harrison TN, Sacks DA, Parry C, et al. Acceptability of Virtual Prenatal Visits for Women with Gestational Diabetes. *Women's Health Issues* 2017; 27: 351-355. DOI: https://doi.org/10.1016/j.whi.2016.12.009.
27. Henry A, Yang J, Grattan S, et al. Effects of the COVID-19 Pandemic and Telehealth on Antenatal Screening and Services, Including for Mental Health and Domestic Violence: An Australian Mixed-Methods Study. *Frontiers in Global Women's Health* 2022; 3. Original Research. DOI: 10.3389/fgwh.2022.819953.
28. Hinton L, Dakin FH, Kuberska K, et al. Quality framework for remote antenatal care: qualitative study with women, healthcare professionals and system-level stakeholders. *BMJ Quality & Safety* 2022; 12: 12. DOI: 10.1136/bmjqs-2021-014329.
29. Hinton L, Kuberska K, Dakin F, et al. A qualitative study of the dynamics of access to remote antenatal care through the lens of candidacy. *J Health Serv Res Policy* 2023. DOI: 10.1177/13558196231165361.
30. Jeganathan S, Prasannan L, Blitz MJ, et al. Adherence and acceptability of telehealth appointments for high-risk obstetrical patients during the coronavirus disease 2019 pandemic. *Am J Obstet Gynecol MFM* 2020; 2: 100233. DOI: 10.1016/j.ajogmf.2020.100233.
31. Karavadra B, Stockl A, Prosser-Snelling E, et al. Women's perceptions of COVID-19 and their healthcare experiences: a qualitative thematic analysis of a national survey of pregnant women in the United Kingdom. *BMC Pregnancy and Childbirth* 2020; 20: 600. DOI: 10.1186/s12884-020-03283-2.
32. Khalil C. Understanding the Adoption and Diffusion of a Telemonitoring Solution in Gestational Diabetes Mellitus: Qualitative Study. *JMIR Diabetes* 2019; 4: e13661. DOI: https://dx.doi.org/10.2196/13661.
33. Khosla K, Suresh S, Mueller A, et al. Elimination of racial disparities in postpartum hypertension follow-up after incorporation of telehealth into a quality bundle. *American Journal of Obstetrics & Gynecology MFM* 2022; 4: 100580. DOI: https://dx.doi.org/10.1016/j.ajogmf.2022.100580.
34. Klamroth-Marganska V, Gemperle M, Ballmer T, et al. Does therapy always need touch? A cross-sectional study among Switzerland-based occupational therapists and midwives regarding their experience with health care at a distance during the COVID-19 pandemic in spring 2020. *BMC Health Services Research* 2021; 21: 578. DOI: https://dx.doi.org/10.1186/s12913-021-06527-9.
35. Kluwgant D, Homer C and Dahlen H. "Never let a good crisis go to waste": Positives from disrupted maternity care in Australia during COVID-19. *Midwifery* 2022; 110: 103340. DOI: https://dx.doi.org/10.1016/j.midw.2022.103340.
36. Kozica-Olenski SL, Soldatos G, Marlow L, et al. Exploring the acceptability and experience of receiving diabetes and pregnancy care via telehealth during the COVID-19 pandemic: a qualitative study. *BMC Pregnancy and Childbirth* 2022; 22: 932. DOI: https://dx.doi.org/10.1186/s12884-022-05175-z.

37. Krenitsky NM, Spiegelman J, Sutton D, et al. Primed for a pandemic: Implementation of telehealth outpatient monitoring for women with mild COVID-19. *Seminars in Perinatology* 2020; 44: 151285. DOI: https://dx.doi.org/10.1016/j.semperi.2020.151285.
38. Lapadula MC, Rolfs S, Szyld EG, et al. Evaluating Patients' and Neonatologists' Satisfaction With the Use of Telemedicine for Neonatology Prenatal Consultations During the COVID-19 Pandemic. *Frontiers in Pediatrics* 2021; 9: 642369. DOI: https://dx.doi.org/10.3389/fped.2021.642369.
39. Leighton C, Conroy M, Bilderback A, et al. Implementation and Impact of a Maternal-Fetal Medicine Telemedicine Program. *Am J Perinatol* 2019; 36: 751-758. DOI: https://dx.doi.org/10.1055/s-0038-1675158.
40. Liu CH, Goyal D, Mittal L, et al. Patient Satisfaction with Virtual-Based Prenatal Care: Implications after the COVID-19 Pandemic. *Maternal and Child Health Journal* 2021; 25: 1735-1743. DOI: 10.1007/s10995-021-03211-6.
41. Madden N, Emeruwa UN, Friedman AM, et al. Telehealth Uptake into Prenatal Care and Provider Attitudes during the COVID-19 Pandemic in New York City: A Quantitative and Qualitative Analysis. *Am J Perinatol* 2020; 37: 1005-1014. DOI: 10.1055/s-0040-1712939.
42. Mann C, Goodhue B, Guillard A, et al. The COVID-19 pandemic and reproductive genetic counseling: Changes in access and service delivery at an academic medical center in the United States. *Journal of Genetic Counseling* 2021; 30: 958-968. DOI: 10.1002/jgc4.1462.
43. Mehl SC, Short WD, Powell P, et al. Impact of Telemedicine on Prenatal Counseling at a Tertiary Fetal Center: A Mixed Methods Study. *The Journal of Surgical Research* 2022; 280: 288-295. DOI: https://dx.doi.org/10.1016/j.jss.2022.07.020.
44. Moltrecht B, Dalton LJ, Hanna JR, et al. Young parents' experiences of pregnancy and parenting during the COVID-19 pandemic: a qualitative study in the United Kingdom. *BMC Public Health* 2022; 22: 523. DOI: https://doi.org/10.1186/s12889-022-12892-9.
45. Moltrecht B, de Cassan S, Rapa E, et al. Challenges and opportunities for perinatal health services in the COVID-19 pandemic: a qualitative study with perinatal healthcare professionals. *BMC Health Services Research* 2022; 22: 1026. DOI: https://dx.doi.org/10.1186/s12913-022-08427-y.
46. Morgan A, Goodman D, Vinagolu-Baur J, et al. Prenatal telemedicine during COVID-19: patterns of use and barriers to access. *JAMIA Open* 2022; 5: ooab116. DOI: https://dx.doi.org/10.1093/jamiaopen/ooab116.
47. Nelson GA and Holschuh C. Evaluation of Telehealth Use in Prenatal Care for Patient and Provider Satisfaction: A Step Toward Reducing Barriers to Care. *J Nurse Pract* 2021; 17: 481-484. DOI: 10.1016/j.nurpra.2020.12.026.
48. Oelmeier K, Schmitz R, Moellers M, et al. Satisfaction with and Feasibility of Prenatal Counseling via Telemedicine: A Prospective Cohort Study. *Telemedicine e-Health* 2022; 28: 1193-1198. DOI: 10.1089/tmj.2021.0309.
49. Osarhiemen OA, Robinson MA, Zhao Z, et al. Assessing access to obstetrical care via telehealth in the era of COVID-19. *American Journal of Obstetrics and Gynecology* 2022; 226: 429-432. DOI: 10.1016/j.ajog.2021.09.011.
50. Palmer KR, Tanner M, Davies-Tuck M, et al. Widespread implementation of a low-cost telehealth service in the delivery of antenatal care during the COVID-19 pandemic: an interrupted time-series analysis. *The Lancet* 2021; 398: 41-52. DOI: https://doi.org/10.1016/S0140-6736(21)00668-1.
51. Peahl AF, Powell A, Berlin H, et al. Patient and provider perspectives of a new prenatal care model introduced in response to the coronavirus disease 2019 pandemic. *American Journal of Obstetrics and Gynecology* 2021; 224: 384.e381-384.e311. DOI: https://doi.org/10.1016/j.ajog.2020.10.008.
52. Pflugeisen BM, McCarren C, Poore S, et al. Virtual Visits: Managing prenatal care with modern technology. *MCN The American Journal of Maternal Child Nursing* 2016; 41: 24-30. DOI: https://dx.doi.org/10.1097/NMC.0000000000000199.
53. Pflugeisen BM and Mou J. Patient Satisfaction with Virtual Obstetric Care. *Matern Child Health J* 2017; 21: 1544-1551. DOI: 10.1007/s10995-017-2284-1.
54. Quinn LM, Olajide O, Green M, et al. Patient and Professional Experiences With Virtual Antenatal Clinics During the COVID-19 Pandemic in a UK Tertiary Obstetric Hospital: Questionnaire Study. *J Med Internet Res* 2021; 23: e25549. DOI: 10.2196/25549.
55. Rasekaba T, Nightingale H, Furler J, et al. Women, clinician and IT staff perspectives on telehealth for enhanced gestational diabetes mellitus management in an Australian rural/regional setting. *Rural and Remote Health* 2021; 21: 5983. DOI: https://dx.doi.org/10.22605/RRH5983.

56. Rayment-Jones H, Dalrymple K, Harris JM, et al. Project20: maternity care mechanisms that improve access and engagement for women with social risk factors in the UK – a mixed-methods, realist evaluation. *BMJ Open* 2023; 13: e064291. DOI: 10.1136/bmjopen-2022-064291.
57. Rayment-Jones H, Harris J, Harden A, et al. Project20: Maternity care mechanisms that improve (or exacerbate) health inequalities. A realist evaluation. *Women Birth* 2022. DOI: https://dx.doi.org/10.1016/j.wombi.2022.11.006.
58. Reid CN, Marshall J and Fryer K. Evaluation of a Rapid Implementation of Telemedicine for Delivery of Obstetric Care During the COVID-19 Pandemic. *medRxiv* 2021: 2021.2005.2019.21257311. DOI: 10.1101/2021.05.19.21257311.
59. Rousseau A, Gaucher L, Gautier S, et al. How midwives implemented teleconsultations during the COVID-19 health crisis: a mixed-methods study. *BMJ Open* 2022; 12: e057292. DOI: https://dx.doi.org/10.1136/bmjopen-2021-057292.
60. Saad M, Chan S, Nguyen L, et al. Patient perceptions of the benefits and barriers of virtual postnatal care: a qualitative study. *BMC Pregnancy and Childbirth* 2021; 21: 543. DOI: 10.1186/s12884-021-03999-9.
61. Sanders J and Blaylock R. “Anxious and traumatised”: Users’ experiences of maternity care in the UK during the COVID-19 pandemic. *Midwifery* 2021; 102: 103069. DOI: https://doi.org/10.1016/j.midw.2021.103069.
62. Sarre G, Hyer S, Chauhan-Whittingham P, et al. Patients' experience of antenatal diabetic care during the current COVID-19 pandemic: an exploratory study. *Practical Diabetes* 2021; 38: 23-30. DOI: https://dx.doi.org/10.1002/pdi.2367.
63. Shashikumar A, Okesene-Gafa K, Apaapa-Timu T, et al. Teleclinics for the management of diabetes in pregnancy during COVID-19 —maternal satisfaction and pregnancy outcomes. *New Zealand Medical Journal* 2022; 135: 63-77.
64. Shaw S, Wherton J, Vijayaraghavan S, et al. Advantages and limitations of virtual online consultations in a NHS acute trust: the VOCAL mixed-methods study. *NIHR Journals Library (Health Services and Delivery Research)* 2018. DOI: https://dx.doi.org/10.3310/hsdr06210.
65. Silverio SA, De Backer K, Easter A, et al. Women's experiences of maternity service reconfiguration during the COVID-19 pandemic: A qualitative investigation. *Midwifery* 2021; 102: 103116. DOI: 10.1016/j.midw.2021.103116.
66. Smith AD, Z; Farmer, D; Stacey, T *Using Maternity Services During COVID-19*. 2020. Yorkshire and Harrogate Maternity Voices Partnership; University of Huddersfield https://www.maternityvoices.co.uk/content/uploads/2021/01/Covid-19-Maternity-Report.pdf [accessed 07/07/21]
67. Smith VJ, Marshall A, Lie MLS, et al. Implementation of a fetal ultrasound telemedicine service: women's views and family costs. *BMC Pregnancy and Childbirth* 2021; 21: 38. DOI: https://dx.doi.org/10.1186/s12884-020-03532-4.
68. Spiby H, Faucher MA, Sands G, et al. A qualitative study of midwives' perceptions on using video-calling in early labor. *Birth* 2019; 46: 105-112. DOI: 10.1111/birt.12364.
69. Stacey T, Darwin Z, Keely A, et al. Experiences of maternity care during the COVID-19 pandemic in the North of England. *British Journal of Midwifery* 2021; 29: 516-523. DOI: 10.12968/bjom.2021.29.9.516.
70. Sullivan MW, Kanbergs AN, Burdette ER, et al. Acceptability of virtual prenatal care: thinking beyond the pandemic. *The Journal of Maternal-Fetal & Neonatal Medicine* 2021: 1-4. DOI: https://dx.doi.org/10.1080/14767058.2021.1980534.
71. Sung Y-S, Zhang D, Eswaran H, et al. Evaluation of a telemedicine program managing high-risk pregnant women with pre-existing diabetes in Arkansas's Medicaid program. *Seminars in Perinatology* 2021; 45: 151421. DOI: https://dx.doi.org/10.1016/j.semperi.2021.151421.
72. Talmont E and Vitale TR. Telehealth Readiness Assessment of Perinatal Nurses. *Nursing for Women's Health* 2022; 26: 86-94. DOI: https://dx.doi.org/10.1016/j.nwh.2022.01.004.
73. Tavener CR, Kyriacou C, Elmascri I, et al. Rapid introduction of virtual consultation in a hospital-based Consultant-led Antenatal Clinic to minimise exposure of pregnant women to COVID-19. *BMJ Open Qual* 2022; 11: e001622. DOI: 10.1136/bmjoq-2021-001622.
74. Theiler RN, Butler-Tobah Y, Hathcock MA, et al. OB Nest randomized controlled trial: a cost comparison of reduced visit compared to traditional prenatal care. *BMC Pregnancy and Childbirth* 2021; 21: 71. DOI: 10.1186/s12884-021-03557-3.
75. Tozour JN, Bandremer S, Patberg E, et al. Application of telemedicine video visits in a maternal-fetal medicine practice at the epicenter of the COVID-19 pandemic. *American Journal of Obstetrics & Gynecology MFM* 2021; 3: 100469. DOI: https://dx.doi.org/10.1016/j.ajogmf.2021.100469.

76. van den Heuvel JFM, Ayubi S, Franx A, et al. Home-Based Monitoring and Telemonitoring of Complicated Pregnancies: Nationwide Cross-Sectional Survey of Current Practice in the Netherlands. *JMIR mHealth and uHealth* 2020; 8: e18966. DOI: https://dx.doi.org/10.2196/18966.
77. Zulifqar BA. *Providers' Satisfaction with Provision of Prenatal Care During the COVID-19 Pandemic*. M.S., University of North Texas Health Science Center at Fort Worth, Ann Arbor, 2021.
78. Almuslim H and AlDossary S. Models of Incorporating Telehealth into Obstetric Care During the COVID-19 Pandemic, Its Benefits And Barriers: A Scoping Review. *Telemed J E Health* 2022; 28: 24-38. DOI: 10.1089/tmj.2020.0553.
79. Bailey CM, Newton JM and Hall HG. Telephone triage and midwifery: A scoping review. *Women & Birth* 2018; 31: 414-421. DOI: 10.1016/j.wombi.2017.12.002.
80. Cantor AG, Jungbauer RM, Totten AM, et al. Telehealth Strategies for the Delivery of Maternal Health Care: A Rapid Review. *Annals of Internal Medicine* 2022; 175: 1285-1297. DOI: https://dx.doi.org/10.7326/M22-0737.
81. Chua CMS, Mathews J, Ong MSB, et al. Use of telelactation interventions to improve breastfeeding outcomes among mothers: A mixed-studies systematic review. *Women Birth* 2022. DOI: https://dx.doi.org/10.1016/j.wombi.2022.06.011.
82. Fernandez Turienzo C, Rayment-Jones H, Roe Y, et al. A realist review to explore how midwifery continuity of care may influence preterm birth in pregnant women. *Birth* 2021; 48: 375-388. DOI: https://dx.doi.org/10.1111/birt.12547.
83. Flaherty SJ, Delaney H, Matvienko-Sikar K, et al. Maternity care during COVID-19: a qualitative evidence synthesis of women's and maternity care providers' views and experiences. *BMC Pregnancy and Childbirth* 2022; 22: 438. DOI: 10.1186/s12884-022-04724-w.
84. Friedemann Smith C, Lunn H, Wong G, et al. Optimising GPs' communication of advice to facilitate patients' self-care and prompt follow-up when the diagnosis is uncertain: A realist review of 'safety-netting' in primary care. *BMJ Quality and Safety* 2022; 31: 541-554. DOI: https://dx.doi.org/10.1136/bmjqs-2021-014529.
85. Ghimire S, Martinez S, Hartvigsen G, et al. Virtual prenatal care: A systematic review of pregnant women's and healthcare professionals' experiences, needs, and preferences for quality care. *International Journal of Medical Informatics* 2023; 170: 104964. DOI: https://doi.org/10.1016/j.ijmedinf.2022.104964.
86. Konnyu KJ, Danilack VA, Adam GP, et al. Changes to Prenatal Care Visit Frequency and Telehealth: A Systematic Review of Qualitative Evidence. *Obstetrics and Gynecology* 2023. DOI: https://dx.doi.org/10.1097/AOG.0000000000005046.
87. Society for Maternal-Fetal Medicine, Healy A, Davidson C, et al. Society for Maternal-Fetal Medicine Special Statement: Telemedicine in Obstetrics - Quality and Safety Considerations. *American Journal of Obstetrics and Gynecology* 2022. DOI: https://dx.doi.org/10.1016/j.ajog.2022.12.002.
88. Wu K, Lopez C and Nichols M. Virtual Visits in Prenatal Care: An Integrative Review. *Journal of Midwifery & Women's Health* 2021. DOI: https://dx.doi.org/10.1111/jmwh.13284.
89. Healthcare Safety Investigation Branch. *National Learning Report Intrapartum Stillbirth: Learning from Maternity Safety Investigations that Occurred during the COVID-19 Pandemic, 1 April to 30 June 2020*. 2021. https://hsib-kqcco125-media.s3.amazonaws.com/assets/documents/HSIB_Intrapartum_Stillbirth_Report_web.pdf [accessed 14/02/23]
90. Healthcare Safety Investigation Branch. *National Learning Report Maternal Death: Learning from Maternal Death Investigations during the First Wave of the COVID-19 Pandemic*. 2021. https://hsib-kqcco125-media.s3.amazonaws.com/assets/documents/HSIB_Maternal_Death_Report_V13.pdf [accessed 14/02/23]
91. Healthcare Safety Investigation Branch. *Assessment of Risk during the Maternity Pathway*. 2023. https://www.hsib.org.uk/investigations-and-reports/assessment-risk-during-maternity-pathway/report/#43-risk-assessment-and-triage [accessed 16/03/23]
92. Knight M, Bunch K, Cairns A, et al. *MBRACE-UK: Saving Lives, Improving Mothers' Care: Rapid report 2021: Learning from SARS-CoV-2-Related and Associated Maternal Deaths in the UK: June 2020-March 2021*. 2021. https://www.npeu.ox.ac.uk/assets/downloads/mbrrace-uk/reports/MBRRACE-UK_Maternal_Report_June_2021_-_FINAL_v10.pdf [accessed 14/02/23]
93. Knight M, Bunch K, Patel R, et al. *MBRACE-UK: Lessons Learned to Inform Maternity Care from the UK and Ireland Confidential Enquiries into Maternal Deaths and Morbidity 2018-20*. 2022. https://www.npeu.ox.ac.uk/assets/downloads/mbrrace-uk/reports/maternal-report-2022/MBRRACE-UK_Maternal_MAIN_Report_2022_v10.pdf [accessed 22/02/23]
