# Supplementary material for: Optimising digital clinical consultations in maternity care: a realist review and implementation principles
Source: BMJ Open. 2024 Nov 1;14(10):e079153. doi: 10.1136/bmjopen-2023-079153 (PMC11529580; doi:10.1136/bmjopen-2023-079153)
Supplement: online supplemental file 13 [file bmjopen-14-10-s013.pdf]

Supplemental File 13: Evidence Sources included in the Longlist but not Prioritised into the Shortlist of the Review (Phases 2&3)

1. Acquavita, S. P., Krummel, D. A., Talks, A., Cobb, A. & Mcclure, E. 2019. Assessing the digital divide among low-income perinatal women: Opportunities for provision of health information and counseling. *Telemedicine and e-Health,* 25**,** 48-54.

2. Aksoy derya, Y., Altiparmak, S., Akça, E., Gökbulut, N. & Yilmaz, A. N. 2021. Pregnancy and birth planning during COVID-19: The effects of tele-education offered to pregnant women on prenatal distress and pregnancy-related anxiety *Midwifery,* 92**,** 102877.

3. Altman, M. R., Gavin, A. R., Eagen-Torkko, M. K., Kantrowitz-Gordon, I., Khosa, R. M. & Mohammed, S. A. 2021. Where the System Failed: The COVID-19 Pandemic's Impact on Pregnancy and Birth Care. *Global Qualitative Nursing Research,* 8**,** 23333936211006397.

4. Aquino, M., Munce, S., Griffith, J., Pakosh, M., Munnery, M. & Seto, E. 2020. Exploring the Use of Telemonitoring for Patients at High Risk for Hypertensive Disorders of Pregnancy in the Antepartum and Postpartum Periods: Scoping Review. *JMIR mHealth and uHealth,* 8**,** e15095.

5. Atmuri, K., Sarkar, M., Obudu, E. & Kumar, A. 2021. Perspectives of pregnant women during the COVID-19 pandemic: A qualitative study. *Women and Birth*.

6. Avercenc, L., Ngueyon Sime, W., Bertholdt, C., Baumont, S., Freitas, A. C. D., Morel, O., Guillemin, F. & Ambroise Grandjean, G. 2022. Improving prenatal care during lockdown: Comparing telehealth and in-person care for low-risk pregnant women in the PROTECT pilot study. *Journal of Gynecology Obstetrics and Human Reproduction,* 51**,** 102445.

7. Bailey, E. & Nightingale, S. 2020. Navigating maternity service redesign in a global pandemic: A report from the field. *Midwifery,* 89**,** 102780-102780.

8. Banerjee, A. & Mansfield, A. 2020. 'Shining a light on the gaps for learning'. *Midwifery,* 89**,** 102790.

9. Barbour, K. D., Nelson, R., Esplin, M. S., Varner, M. & Clark, E. a. S. 2017. 873: A randomized trial of prenatal care using telemedicine for low-risk pregnancies: patient-related cost and time savings. *American Journal of Obstetrics & Gynecology,* 216**,** S499-S499.

10. Barrera, C. M., Powell, A. R., Biermann, C. R., Siden, J. Y., Nguyen, B.-H., Roberts, S. J., James, L., Chopra, V. & Peahl, A. 2021. A Review of Prenatal Care Delivery to Inform the Michigan Plan for Appropriate Tailored Healthcare in Pregnancy Panel. *Obstetrics and Gynecology,* 138**,** 603-615.

11. Bhalla, A., Bhalla, R. & Ganta, S. 2021. Working remotely: a perspective on telemedicine in delivery of obstetrics and gynaecology health care. *Obstetrician and Gynaecologist,* 23**,** 237-242.

12. Bick, D., Cheyne, H., Chang, Y.-S. & Fisher, J. 2020. Maternal postnatal health during the COVID-19 pandemic: Vigilance is needed. *Midwifery,* 88**,** 102781.

13. Bradfield, Z., Hauck, Y., Homer, C. S. E., Sweet, L., Wilson, A. N., Szabo, R. A., Wynter, K., Vasilevski, V. & Kuliukas, L. 2021. Midwives' experiences of providing maternity care

during the COVID-19 pandemic in Australia. *Women and Birth: Journal of the Australian College of Midwives*.

14. Bradley, D., Blaine, A., Shah, N., Mehrotra, A., Gupta, R. & Wolfberg, A. 2020. Patient Experience of Obstetric Care During the COVID-19 Pandemic: Preliminary Results From a Recurring National Survey. *Journal of Patient Experience,* 7**,** 653-656.

15. Brislane, Á., Larkin, F., Jones, H. & Davenport, M. H. 2021. Access to and Quality of Healthcare for Pregnant and Postpartum Women During the COVID-19 Pandemic. *Frontiers in Global Women's Health,* 2.

16. Ceesay, M., Ravi, S., Mumford, K., Alvarez, M., Ghartey, J., Harper, L. M. & Cahill, A. G. 2023. Telehealth since the COVID-19 pandemic: can it improve prenatal care and birth outcomes? *American Journal of Obstetrics & Gynecology,* 228**,** S529-S530.

17. Chan, C. B., Popeski, N., Hassanabad, M. F., Sigal, R. J., O'connell, P. & Sargious, P. 2021. Use of Virtual Care for Glycemic Management in People With Types 1 and 2 Diabetes and Diabetes in Pregnancy: A Rapid Review. *CanadianJjournal of Diabetes,* 45**,** 677-688.e2.

18. Davis, A. & Bradley, D. 2022. Telemedicine utilization and perceived quality of virtual care among pregnant and postpartum women during the COVID-19 pandemic. *Journal of Telemedicine and Telecare***,** 1357633X221133862.

19. Department of Health and Social Care 2022. Final Report of the Ockenden Review: Findings, conclusions and essential actions from the independent review of maternity services at the Shrewsbury and Telford Hospital NHS Trust.

20. Fantinelli, S., Marchetti, D., Verrocchio, M. C., Franzago, M., Fulcheri, M. & Vitacolonna, E. 2019. Assessment of Psychological Dimensions in Telemedicine Care for Gestational Diabetes Mellitus: A Systematic Review of Qualitative and Quantitative Studies. *Frontiers in Psychology,* 10**,** 153.

21. Ferraz Dos Santos, L., Borges, R. F. & De Azambuja, D. A. 2020. Telehealth and Breastfeeding: An Integrative Review. *Telemed J E Health,* 26**,** 837-846.

22. Futterman, I., Rosenfeld, E., Toaff, M., Boucher, T., Golden-Espinal, S., Evans, K. & Clare, C. A. 2021. Addressing Disparities in Prenatal Care via Telehealth During COVID-19: Prenatal Satisfaction Survey in East Harlem. *American Journal of Perinatology,* 38**,** 88-92.

23. Gavine, A., Marshall, J., Buchanan, P., Cameron, J., Leger, A., Ross, S., Murad, A. & Mcfadden, A. 2022. Remote provision of breastfeeding support and education: Systematic review and meta-analysis. *Maternal & Child Nutrition,* 18**,** 1-23.

24. Grassl, N., Nees, J., Schramm, K., Spratte, J., Sohn, C., Schott, T. C. & Schott, S. 2018. A Web-Based Survey Assessing the Attitudes of Health Care Professionals in Germany Toward the Use of Telemedicine in Pregnancy Monitoring: Cross-Sectional Study. *JMIR mHealth and uHealth,* 6**,** e10063.

25. Guille, C., Johnson, E., Douglas, E., Aujla, R., Boyars, L., Kruis, R., Beeks, R., King, K., Ford, D. & Sterba, K. 2022. A Pilot Study Examining Access to and Satisfaction with Maternal Mental Health and Substance Use Disorder Treatment via Telemedicine. *Telemedicine Reports,* 3**,** 24-29.

26. Guille, C., Simpson, A. N., Douglas, E., Boyars, L., Cristaldi, K., Mcelligott, J., Johnson, D. & Brady, K. 2020. Treatment of Opioid Use Disorder in Pregnant Women via Telemedicine: A Nonrandomized Controlled Trial. *JAMA Network Open,* 3**,** e1920177.

27. Hargis-Villanueva, A., Lai, K., Van Leeuwen, K., Weidler, E. M., Felts, J., Schmidt, A., Franklin, W. J., Lindblade, C., Martin, G. C., Patil, A. S. & Goncalves, L. F. 2022. Telehealth multidisciplinary prenatal consultation during the COVID-19 pandemic: enhancing patient care coordination while maintaining high provider satisfaction. *The Journal of Maternal-Fetal & Neonatal Medicine: the official journal of the European Association of Perinatal Medicine, the Federation of Asia and Oceania Perinatal Societies, the International Society of Perinatal Obstetricians***,** 1-5.

28. Hearn, F., Biggs, L., Wallace, H. & Riggs, E. 2021. No one asked us: Understanding the lived experiences of midwives providing care in the north west suburbs of Melbourne during the COVID-19 pandemic: An interpretive phenomenology. *Women and Birth: Journal of the Australian College of Midwives*.

29. Hertle, D., Wende, D., Schumacher, L. & Bauer, N. H. 2022. Midwives' and women's views on digital midwifery care in Germany: Results from an online survey. *Midwifery,* 115.

30. Hinton, L., Kuberska, K., Dakin, F., Dixon-Woods, M. & Ekechi, C. 2021. Creating equitable remote antenatal care: the importance of inclusion. *BMJ Opinion*.

31. Hofmann, G., Hampanda, K., Harrison, M. S., Fasano, M., Nacht, A. & Yeoman, M. 2022. Virtual Prenatal and Postpartum Care Acceptability Among Maternity Care Providers. *Maternal and Child Health Journal,* 26**,** 1401-1408.

32. Holcomb, D., Faucher, M. A., Bouzid, J., Quint-Bouzid, M., Nelson, D. B. & Duryea, E. 2020. Patient Perspectives on Audio-Only Virtual Prenatal Visits Amidst the Severe Acute Respiratory Syndrome Coronavirus 2 (SARS-CoV-2) Pandemic. *Obstetrics & Gynecology,* 136.

33. Jack, S. M., Munro-Kramer, M. L., Williams, J. R., Schminkey, D., Tomlinson, E., Jennings Mayo-Wilson, L., Bradbury-Jones, C. & Campbell, J. C. 2021. Recognising and responding to intimate partner violence using telehealth: Practical guidance for nurses and midwives. *Journal of Clinical Nursing,* 30**,** 588-602.

34. Jacobsen, K. E., Katon, J. G. & Kantrowitz-Gordon, I. 2022. Midwifery in the Time of COVID-19: An Exploratory Study from the Perspectives of Community Midwives. *Women's Health Issues*.

35. Jardine, J., Relph, S., Magee, L. A., Von Dadelszen, P., Morris, E., Ross-Davie, M., Draycott, T. & Khalil, A. 2020. Maternity services in the UK during the coronavirus disease 2019 pandemic: a national survey of modifications to standard care. *BJOG*.

36. Jennifer Yang, J. Y., Louise Everitt, L. E., Sarah Grattan, S. G., Lynne Roberts, L. R., Anne Lainchbury, A. L., Janani Shanthosh, J. S., Patricia Cullen, P. C. & Amanda Henry, A. H. 2022. Maternity staff perceptions of effects on antenatal screening and services for mental health and domestic violence during the covid-19 pandemic: a survey study. *Journal of Paediatrics and Child Health,* 58**,** 75.

37. Jensen, N. H., Nielsen, K. K., Dahl-Petersen, I. K. & Maindal, H. T. 2022. The experience of women with recent gestational diabetes during the COVID-19 lockdown: a qualitative study from Denmark. *BMC Pregnancy and Childbirth,* 22**,** 84.

38. John, J. R., Curry, G. & Cunningham-Burley, S. 2021. Exploring ethnic minority women's experiences of maternity care during the SARS-CoV-2 pandemic: a qualitative study. *BMJ Open,* 11**,** e050666.

39. Jurgiel, J., Graniak, A., Jozwik, K. & Pomorski, M. 2022. Pregnancy and childbirth during the coronavirus pandemic. The cross-sectional study of 1321 participants in Poland. *Ginekologia Polska*.

40. Kaarthikayinie, T., Kirsten, P., Miranda, D. T. & Ryan, H. 2022. Telehealth-integrated antenatal care - a year in review. *Journal of Paediatrics and Child Health,* 58**,** 24.

41. Keating, N., Dempsey, B., Corcoran, S., Lalor, J. & Higgins, M. 2020. Women's Experience of Pregnancy and Birth during the Covid-19 Pandemic: A Qualitative Study. *Research Square*.

42. Kennedy, H. P., Faucher, M. A., Roberts, J., Divall, B. & Spiby, H. 2018. The Use of Video Calls in Early Labor Care: Exploring Midwives' Views of the Potential Benefits and Challenges...American College of Nurse-Midwives' 63rd Annual Meeting, Georgia. *Journal of Midwifery & Women's Health,* 63**,** 624-625.

43. Kobayashi, H. & Sado, T. 2019. Satisfaction of a new telephone consultation service for prenatal and postnatal health care. *The Journal of Obstetrics and Gynaecology Research,* 45**,** 1376-1381.

44. Kolker, S., Biringer, A., Bytautas, J., Blumenfeld, H., Kukan, S. & Carroll, J. C. 2021. Pregnant during the COVID-19 pandemic: an exploration of patients' lived experiences. *BMC Pregnancy and Childbirth,* 21**,** 851-851.

45. Kuberska, K., Dakin, F., Dixon-Woods, M., Ekechi, C. & Hinton, L. 2021. Creating an equitable evidence base for quality and safety in remote antenatal care. *Authorea (Pre-Print)*.

46. Kumar, N. R., Arias, M. P., Leitner, K., Wang, E., Clement, E. G. & Hamm, R. F. 2023. Assessing the impact of telehealth implementation on postpartum outcomes for Black birthing people. *American Journal of Obstetrics and Gynecology MFM,* 5**,** 100831.

47. Lanssens, D., Vandenberk, T., Lodewijckx, J., Peeters, T., Storms, V., Thijs, I. M., Grieten, L. & Gyselaers, W. 2019. Midwives', Obstetricians', and Recently Delivered Mothers' Perceptions of Remote Monitoring for Prenatal Care: Retrospective Survey. *J Med Internet Res,* 21**,** e10887.

48. Lanssens, D., Vandenberk, T., Smeets, C. J., De Canniere, H., Vonck, S., Claessens, J., Heyrman, Y., Vandijck, D., Storms, V., Thijs, I. M., Grieten, L. & Gyselaers, W. 2018. Prenatal Remote Monitoring of Women With Gestational Hypertensive Diseases: Cost Analysis. *Journal of Medical Internet Research,* 20**,** e102.

49. Lie, M., Snaith, V., Bidmead, E., Marshall, A. & Robson, S. C. 2017. Patients' views of a fetal ultrasound telemedicine service: A mixed methods evaluation study. *BJOG: An International Journal of Obstetrics and Gynaecology,* 124**,** 40.

50. Lister, R., Osarhiemen, O., Robinson, M., Zhao, Z., Ding, T., Mann, C., Bennett, K., Patel, S., Garrison, E. & Crants, S. 2021. 959 The silver lining to Covid-19: access to obstetric care via telehealth. *American Journal of Obstetrics and Gynecology,* 224**,** S595.

51. Mallampati, D., Talati, A. N., Honart, A. W., Johnson, J. D., Vladutiu, C. & Menard, K. 2021. 971 Preferences and perceived needs for telehealth use among maternity care providers nationally. *American Journal of Obstetrics and Gynecology,* 224**,** S602.

52. Malloy, S. & Bradley, D. 2022. A case for retaining obstetric telemedicine in the post-pandemic world. *American Journal of Obstetrics and Gynecology,* 226**,** S677.

53. Marcucci, B. 2018. Use of Telehealth to Increase Breastfeeding Exclusivity and Duration. *Clinical Lactation,* 9**,** 66-71.

54. Marshall, C., Gutierrez, S., Hecht, H., Logan, R., Kerns, J. & Diamond-Smith, N. 2023. Quality of prenatal and postpartum telehealth visits during COVID-19 and preferences for future care. *AJOG Global Reports,* 3**,** 100139.

55. Mckinlay, A. R., Fancourt, D. & Burton, A. 2021. Factors affecting the mental health of pregnant women using UK maternity services during the COVID-19 pandemic: A qualitative interview study. *medRxiv***,** 2021.10.20.21265279.

56. Mizrak Sahin, B. & Kabakci, E. N. 2021. The experiences of pregnant women during the COVID-19 pandemic in Turkey: A qualitative study. *Women Birth,* 34**,** 162-169.

57. Montagnoli, C., Zanconato, G., Ruggeri, S., Cinelli, G. & Tozzi, A. E. 2021. Restructuring maternal services during the covid-19 pandemic: Early results of a scoping review for non-infected women. *Midwifery,* 94**,** 102916.

58. Nakagawa, K., Umazume, T., Mayama, M., Chiba, K., Saito, Y., Noshiro, K., Morikawa, M., Yoshino, M. & Watari, H. 2021. Survey of attitudes of individuals who underwent remote prenatal check-ups and consultations in response to the COVID-19 pandemic. *The Journal of Obstetrics and Gynaecology Research,* 47**,** 2380-2386.

59. National Maternity Review 2016. Better Births: Improving Outcomes of Maternity Services in England: A Five Year Forward View for Maternity Care.

60. NHS Digital Digital Maternity: Harnessing Digital Technology in Maternity Services.

61. NHS Digital 2018. Maternity DMA Report: Digital Maturity Assessment of Maternity Services in England.

62. NHS Digital 2022. Digital Maturity Assessment Regional Summary (LMNS).

63. NHS England Maternity Transformation Programme.

64. NHS England 2020. Better Births Four Years On: A Review of Progress.

65. NHS England & Better Births 2021. Communications toolkit for local maternity teams to improve communications with Black, Asian and minority ethnic women.

66. NHS England & Nhs Improvement 2021. Equity and equality: Guidance for local maternity systems.

67. Norris, K. G., Huang, P. A., Glantz, J. C., Kodam, R.-S. & Anto-Ocrah, M. 2021. A Cross-Cultural Analysis of the COVID-19 Pandemic's Impact on Antenatal Healthcare-Seeking Behaviors in Ghana and the United States. *Journal of Patient Experience,* 8**,** 23743735211062392.

68. Oggero, M. K., Cappello, R. B., Clay, P. C. & Gaskamp, C. R. 2021. Patient Satisfaction With a COVID-19 Telehealth Lactation Care Model. *Clinical Lactation,* 12**,** 58-66.

69. Penny, R. A., Bradford, N. K. & Langbecker, D. 2018. Registered nurse and midwife experiences of using videoconferencing in practice: A systematic review of qualitative studies. *Journal of Clinical Nursing,* 27**,** e739-e752.

70. Quinn, L., Olajide, O., Breslin, E., Tan, B. & Ansar, H. 2020. Virtual antenatal clinics. *British Journal of Midwifery,* 28**,** 680-682.

71. Ragheb, J. W., Kountanis, J. A., Shilling, B. A., Cassidy, R., Mckinney, A. M. & Pancaro, C. 2021. Retrospective study evaluating telehealth antenatal anesthesia consults for high-risk obstetric patients. *The Journal of Maternal-Fetal & Neonatal Medicine : the official journal of the European Association of Perinatal Medicine, the Federation of Asia and Oceania Perinatal Societies, the International Society of Perinatal Obstetricians***,** 1-8.

72. Rayment-Jones, H., Harris, J., Harden, A., Silverio, S. A., Turienzo, C. F. & Sandall, J. 2021. Project20: interpreter services for pregnant women with social risk factors in England: what works, for whom, in what circumstances, and how? *Int J Equity Health,* 20**,** 233.

73. Reforma, L. G., Duffy, C., Collier, A.-R. Y., Wylie, B. J., Shainker, S. A., Golen, T. H., Herlihy, M., Lydeard, A. & Zera, C. A. 2020. A multidisciplinary telemedicine model for management of coronavirus disease 2019 (COVID-19) in obstetrical patients. *American Journal of Obstetrics & Gynecology MFM,* 2**,** 100180.

74. Reisinger-Kindle, K., Qasba, N., Cayton, C., Niakan, S., Knee, A. & Goff, S. L. 2021. Evaluation of rapid telehealth implementation for prenatal and postpartum care visits during the COVID-19 pandemic in an academic clinic in Springfield, Massachusetts, United States of America. *Health Science Reports,* 4**,** e455.

75. Reneker, J. C., Zhang, Y., Young, D. K., Liu, X. & Lutz, E. A. 2022. Use of Telehealth Services for Prenatal Care in Mississippi: Comparison of Pre-COVID-19 Pandemic and Pandemic Obstetric Management. *International Journal of Clinical Practice,* 2022**,** 3535700.

76. Royal College of Midwives 2021a. Digital Technology in Maternity Care: A Position Statement.

77. Royal College of Midwives 2021b. Virtual Consultations: Guidance on Appropriate Application for Virtual Consultations and Practical Tips for Effective Use. RCM.

78. Royal College of Midwives & Royal College of Obstetricians & Gynaecologists 2020. Guidance for Antenatal and Postnatal Services in the evolving Coronavirus (COVID-19) pandemic (Version 3).

79. Royal College of Nursing 2020. Remote consultations guidance under COVID-19 restrictions.

80. Sanghavi, M., Packard, E., Sperling, S., Eberly, L. A., Ambrose, M., Julien, H. M., Hirshberg, A., Adusumalli, S. & Lewey, J. 2022. Telemedicine may increase visit completion rates in postpartum patients with preeclampsia. *PloS One,* 17**,** e0275741.

81. Schmitt, N., Mattern, E., Cignacco, E., Seliger, G., König-Bachmann, M., Striebich, S. & Ayerle, G. M. 2021. Effects of the Covid-19 pandemic on maternity staff in 2020 – a scoping review. *BMC Health Services Research,* 21, 1364.

82. Shields, A. D., Wagner, R. K., Knutzen, D., Deering, S. & Nielsen, P. E. 2020. Maintaining access to maternal fetal medicine care by telemedicine during a global pandemic. *Journal of Telemedicine and Telecare*, 1357633X20957468.

83. Singh, H., Sendejas, M., Pallivathucal, L. B., Gonzalez, M., Decker, S., Pimentel, A. R., Mills, M. D., Ahn, D. T. & Billimek, J. 2019. Using telehealth to enhance engagement and reduce patient burden in the management of gestational diabetes: A randomized trial in a diverse community setting. *Diabetes,* 68.

84. Talati, A. N., Mallampati, D., Johnson, J. D., West-Honart, A., Vladutiu, C. & Menard, M. K. 2021a. 970 Future telehealth use for maternity care beyond COVID19: mixed-methods survey of a regional perinatal health-center. *American Journal of Obstetrics and Gynecology,* 224, S601-S602.

85. Talati, A. N., Mallampati, D., Johnson, J. D., West-Honart, A., Vladutiu, C. & Menard, M. K. 2021b. 1000 Provider satisfaction with telehealth for maternity-care during COVID-19: mixed-methods survey of a regional perinatal health-center. *American Journal of Obstetrics and Gynecology,* 224, S619-S620.

86. Townsend, R., Chmielewska, B., Barratt, I., Kalafat, E., Van Der Meulen, J., Gurol-Urganci, I., O'brien, P., Morris, E., Draycott, T., Thangaratinam, S., Doare, K. L., Ladhani, S., Dadelszen, P. V., Magee, L. A. & Khalil, A. 2021. Global changes in maternity care provision during the COVID-19 pandemic: A systematic review and meta-analysis. *EClinicalMedicine,* 37, 100947.

87. Uscher-Pines, L., Ghosh-Dastidar, B., Bogen, D. L., Ray, K. N., Demirci, J. R., Mehrotra, A. & Kapinos, K. A. 2020. Feasibility and Effectiveness of Telelactation Among Rural Breastfeeding Women. *Acad Pediatr,* 20, 652-659.

88. Valverde, V. L., Althaus, E., Horton, L., La Rosa, M. & Haeri, S. 2023. Economic and environmental impact of Maternal-Fetal Telemedicine (teleMFM). *American Journal of Obstetrics & Gynecology,* 228, S231-S231.

89. Van Den Heuvel, J. F., Groenhof, T. K., Veerbeek, J. H., Van Solinge, W. W., Lely, A. T., Franx, A. & Bekker, M. N. 2018. eHealth as the Next-Generation Perinatal Care: An Overview of the Literature. *J Med Internet Res,* 20, e202.

90. Wang, E., Gellman, C., Wood, E., Garvey, K. L., Connolly, C., Barazani, S., Pruzan, A. & Abraham, C. 2022. A Medical Student Postpartum Telehealth Initiative During the COVID-19 Pandemic. *Maternal and Child Health Journal,* 26, 65-69.

91. Wei, L. S., Bennett, T.-a. M., Friedman, S., Chuang, M. & Mehta-Lee, S. 2023. Impact of telehealth on improving access to pregnancy care in an underserved population. *American Journal of Obstetrics & Gynecology,* 228, S524-S524.

92. Westwood, A. R. 2021. Telehealth and maternity: Has the onset of the pandemic changed the way we utilise telemedicine forever? *British Journal of Midwifery,* 29, 352-355.

93. Wilson, H., Tucker, K. L., Chisholm, A., Hodgkinson, J., Lavallee, L., Mackillop, L., Cairns, A. E., Hinton, L., Podschies, C., Chappell, L. C. & Mcmanus, R. J. 2022. Self-

monitoring of blood pressure in pregnancy: A mixed methods evaluation of a national roll-out in the context of a pandemic. *Pregnancy Hypertension,* 30**,** 7-12.

94. Zarasvand, S., Bayar, E., Adan, M., Mountain, K., Lewis, H., Joash, K., Teoh, T. G., Bennett, P. R., Das, S. & Sykes, L. 2020. Rapid quality improvement in a preterm birth clinic care pathway during the COVID-19 pandemic. *BMJ Open Quality,* 9.

95. Zulfeen, M. & Chandrasekaran, N. 2021. Virtual prenatal-care during a pandemic; how satisfying is it to the patients? *BJOG: An International Journal of Obstetrics and Gynaecology,* 128**,** 186.
