# Supplementary material for: Optimising digital clinical consultations in maternity care: a realist review and implementation principles
Source: BMJ Open. 2024 Nov 1;14(10):e079153. doi: 10.1136/bmjopen-2023-079153 (PMC11529580; doi:10.1136/bmjopen-2023-079153)
Supplement: online supplemental file 14 [file bmjopen-14-10-s014.pdf]

SUPPLEMENTAL FILE 14: PROGRAMME THEORY TABLES

Details for each of the 5 programme theory domains are presented in tables below. A key to abbreviations used is in the table below.

**Key for Programme Theory Tables**
**Participant type**:<br>HCP = Healthcare professionals, MW = Midwives, RN = Registered nurses, SU = Service users
**Country:**<br>AUS = Australia, CAN = Canada, IT = Italy, NLD = Netherlands, SUI = Switzerland, UK = United Kingdom, USA = United States of America

Programme Theory Domain 1: Infrastructure and Resources

**Programme Theories: Infrastructure and Resources**	**References**	**Key contexts**	**Examples of supporting data**	**Additional insights from stakeholders**
**1.1. Developing infrastructure**<br>*If organisations take adequate time to provide a digital infrastructure (including reliable equipment, software, internet), developed with staff input to make it user-friendly [**C**], healthcare providers will feel confident [**M**] that digital consultations [**I**] are a tool that can 'fit' into existing work practices [**C**]. Hence, staff will feel motivated [**M**] to embed it into their practice [**O**].*	n=34<br>1-34	• The digital maturity of healthcare facilities and, in the UK, local NHS Trusts.<br>• HCPs working in the community or at home with poor internet connection, limited or outdated devices, with which to conduct digital consultations.<br>• IT support.<br>• National level digital infrastructure e.g. superfast broadband mobile phone network coverage	• "I think it could have been good, if this organization was invested in the equipment… It took me four months to get a computer that was a laptop, and I still haven't been able to crack how to get those two apps on my desktop. . . so I still cannot work remotely."[11] HCP, AUS<br>• "I think that our facility underestimated the time commitment that putting a program like this [telehealth] in place requires."[4] HCP, USA<br>• "There's constantly a push for things to be digital; and there are huge advantages of that, but, until you make internet free for	• UK staff often use mainstream software and applications to facilitate women's engagement and access to DC-CON, even though these technologies are not approved by the NHS.<br>• Poor resourcing from employers cause many HCPs to rely on their personal devices and internet allowances to conduct DC-CONs.<br>• The poor quality and inaccessibility of NHS DC-CON software,

Programme Theories: Infrastructure and Resources	References	Key contexts	Examples of supporting data	Additional insights from stakeholders
			everyone and give everyone a smart phone, then, you know, the people that really need us are the ones that get left behind."[12] HCP, UK<br>• "WhatsApp would be very accessible because every woman has WhatsApp on their phone. Everybody knows how to use it."[2] MW, UK/IT	which often lacks interoperability with other systems (e.g. medical records), makes digital consultations challenging.
**1.2. Establishing clinical systems and pathways**<br><br>*If digital consultations [**I**] are supported by administrative systems and integrated electronic patient record systems that can operate across contexts [**C**], it will improve the ability of staff to access information, work in multi-disciplinary teams and coordinate care across the pathway [**M**]. When systems work well, digital consultations are perceived by staff to improve existing workflows - increasing convenience, efficiency and reducing workload [**O**] – for organisations, staff and service users – as well as maintaining safety [**O**].*	n=33<br>1, 3, 4, 10-13, 15, 18-20, 22-25, 27, 28, 32, 33, 35-47	• HCPs offsite in the community or at-home who need access to medical systems and records.<br>• Multi-disciplinary teams with HCPs working in different locations but who need to make joint decisions about care plans.<br>• Women trying to contact and access maternity care services.	• "You have a lot more leg work to make the two (Attend Anywhere and hospital appointment system) combine…well, they don't. I've got this form as I said, I have to fill in and then save it in their file and retrieve it when I need it. You know, that's a bit of a hassle."[12] HCP, UK<br>• "So, in the video clinics they will have a regular appointment with the diabetes specialist nurse and the diabetes specialist dietician, and for our ladies with Type one or Type two diabetes with the consultant as well. So, we can all still have that joint decision-making but just on a video, virtual clinic rather than a face-to-face clinic."[12] HCP, UK<br>• "The fact that the link is there you can talk directly to [specialists] about other service users. Just little worries… that you've no idea what it is; it's worth saying can	• The HP-SG considered access to women's current and previous records, referrals and notes (including safeguarding concerns) as key to delivering safe digital care.

Programme Theories: Infrastructure and Resources	References	Key contexts	Examples of supporting data	Additional insights from stakeholders
			you just cast your eye over these pictures?”[1] HCP, UK<br>• “they [administrative teams] did the heavy lifting that made this [telehealth delivery] possible”[15] HCP, AUS	
**1.3. Appropriate staffing models and conditions**<br><br>*If staffing models for digital consultations include dedicated teams in private spaces with the capacity to provide continuity of carer [**C**], this type of working environment can enhance staff and women’s sense of privacy and comfort [**M**] facilitating the communication of concerns and treatment [**O**]. This helps women and staff feel confident and motivated [**M**] to use digital consultations (and sustain their use) [**O**].*	n=15<br>1-3, 12, 15, 16, 19, 23, 25, 32, 33, 41, 46, 48, 49	• All HCPs providing DC-CONs.<br>• Management/senior staff responsible for allocating staff to teams and providing appropriate workspaces.<br>• Staff currently providing digital care in silos, onsite or offsite (perhaps working from home) who lack communicative spaces.	• “The designated midwife should have a dedicated space so that she can fully focus on what she is doing.”[2] MW, UK/IT<br>• “We’d need a private space in the hospital, and what comes to my mind are those old- fashioned telephone booths, you know, [laughs] where you go in and you close the door.”[23] MW, UK/USA<br>• “I think there’s less of the kind of corridor conversations that were really good with colleagues both in terms of advancing clinical knowledge, working out management plans for patients, but also just making sure that your colleagues are okay.” [15] HCP, AUS<br>• [Some] maternity providers were found to not have dedicated telephone triage lines. This meant that calls were taken in a variety of locations by differing healthcare professionals. In some cases, calls were answered by non-registered staff. This led to variable information and advice being given.[33] UK	• In practice, it is often difficult for staff to find private spaces at work and that women felt uncomfortable if, for example, they saw other HCPs in the background of a video call.<br>• One driver for implementing DC-CON in UK maternity care was to help services cope with reduced workforces, or similarly, to help services retain staff by offering them more flexible working patterns.

Programme Theory Domain 2: Training and Support for Staff

Programme Theories: Training & Support for Staff	References	Key contexts	Examples of supporting data	Additional insights from stakeholders
**2.1. Providing staff training and ongoing support**<br><br>*If NHS and professional organisations provide a supportive and enabling workplace culture for digital clinical consultations (including sufficient training, protected time for training, appropriate workspaces and ongoing access to clinical, technical and administrative support) [**C**], staff will gain relevant knowledge/skills [**M**] and will feel more motivated, supported and confident [**M**], leading to appropriate and sustained uptake of digital consultations [**O**].*	n=23<br>1-7, 9, 10, 14-16, 18, 20, 23, 30-33, 41, 48-50	• Staff who are unfamiliar with digital technology.<br>• Staff who are new to providing maternity care digitally or who are unfamiliar with the systems, software and procedures used to deliver DC-CON locally.	• "Comprehensive training on empathic communication and on all the abilities necessary to help the woman gain confidence already at your first contact."[2] MW, UK/IT.<br>• The majority of staff surveyed were in support of training for virtual clinics, which is not routinely part of the curriculum, and we anticipate this would further improve efficiency, satisfaction, and ease of adaptation to virtual clinics.[18] UK.<br>• The participants stated that ongoing training is a facilitating factor in continuous stimulation of daily use. In addition, nurses who work as triage staff need to be well supported in their new task. In addition to performing obstetric triage, appropriate support services, such as administration and equipment, must be facilitated.[6] NLD.<br>• Telephone triage services should be operated by appropriately trained and competent clinicians who are skilled in the specific needs required for effective telephone triage.[33] UK	• Training and support were presented as an essential feature that influenced a HCP's cognitive participation and collective action in implementing DC-CON.<br>• It was also acknowledged that staff already have a lot of training to complete and ideally need protected time to complete additional comprehensive DC-CON training.

Programme Theories: Training & Support for Staff	References	Key contexts	Examples of supporting data	Additional insights from stakeholders
**2.2. Ensuring staff motivation and 'buy-in'**<br>*If staff are informed about the potential benefits of DC-CON [C], to both HCPs and women, it can promote staff 'buy-in'. In particular, if staff perceive [M] that women accept, are benefitting from, and satisfied [O] with, digital consultations they will be motivated [M] to use it (buy into and sustain its use) [O] and gain job satisfaction from using it [O].*	n=21<br>1, 2, 4, 12, 14, 15, 18, 20, 21, 23, 26, 28, 30, 34, 36, 41, 46, 51-54	• Staff who do not understand why DC-CON is being offered and/or the potential benefits for women and staff.<br>• Older staff who may be unfamiliar and/or unmotivated to use DC-CON.	• "I don't yet have the evidence I would like about the impact on women, about the acceptability from women, about whether women prefer this style."[12] HCP, UK<br>• "[…] there are a number of midwives that are approaching retirement age that would say they are not very digitally savvy, so it's been difficult for them. And they have probably used the telephone more than video appointments. So, that has certainly been a problem for people"[12] HCP, UK<br>• "I love connecting with these women [via telephone] and providing them the resources they need. They are truly appreciative of all we are able to do for them."[4] HCP, USA.<br>• "Saving time ", "Saving travel time ", "The appointment is done efficiently ", "Working more efficiently"[52] HCPs, SUI.	• Knowing women's thoughts on DC-CON helped staff make sense of why they were offering DC-CON and its coherence with the wider maternity service.<br>• Whilst DC-CON can reduce workloads in some settings, by enabling staff to focus on those most in need, it can increase workloads due to the increased flexibility, 'unseen' administration and pressure to fill the day with appointments and meetings.<br>• A shared commitment to DC-CON from staff at all levels was considered necessary for sustained use.<br>• 'Digital champions' were suggested to support and motivate staff with DC-CON.<br>• DC-CONs can benefit staff with health conditions that make in-person appointments difficult and/or risky.
**2.3. Providing clinical protocols on consultation mode**<br>*If digital consultations are guided by clear clinical protocols*	n=23<br>2, 7, 10-13, 15, 16, 19, 20, 23, 24, 26, 32, 33, 35, 41, 55-57 31, 49, 50	• Staff who are new or unfamiliar with digital consultations or local procedures.	• "If you're on a videoconference with somebody and you potentially see something in the background that is either, you're not comfortable with, or is	• Clear evidence-based protocols and guidelines are essential for safe use of DC-CON.

Programme Theories: Training & Support for Staff	References	Key contexts	Examples of supporting data	Additional insights from stakeholders
[C], staff can feel supported [M] in deciding what type of consultation is appropriate to meet women's varied needs and preferences. When digital consultations are further enhanced with the use of at-home monitoring [C], it can provide additional reassurance to professionals and women [M] of the quality and safety of DC-CON [O]. Combined, this can increase staff ability, acceptance and confidence in monitoring and treating women at a distance [M], leading to optimal clinical/safety outcomes [O].		• Staff supporting women with complex pregnancies who may be receiving DC-CON and/or remote monitoring.<br>• Staff who are worried about safety and safeguarding via DC-CON.	potentially illegal then how do you respond to that new information […] what do you do with it?"[23] MW, UK/USA.<br>• Home devices were seen as important for patient and provider comfort— 92.2% of patients (213 of 231) and 95.5% of providers (63 of 66) believed that a home blood pressure cuff was important for virtual prenatal visits, and 84.8% of patients (196 of 231) and 71.2% of providers (47 of 66) believed that a home fetal Doppler was important. [57] USA.<br>• Protocols should be developed for virtual care that seek to reduce variation between providers and specialties and that outline standards by which symptoms and conditions can be managed virtually […] Clinicians should have access to real-time patient data; therefore, remote patient monitoring data, such as blood pressure and glucose, should be reliably collected into the electronic health record.[31] Global.<br>• Triage proformas may be used to conduct a structured assessment of the pregnant woman/person and baby over the telephone. These may contain parameters that specify what actions should	

Programme Theories: Training & Support for Staff	References	Key contexts	Examples of supporting data	Additional insights from stakeholders
			be taken and the urgency of those actions. Some systems use colour-coded visual cues to aid the assessment. These may be electronic, paper based or a combination of both.[33] UK.	

Programme Theory Domain 3: Personalisation and Flexibility for Women

Programme Theories: Personalisation & Flexibility for Women	References	Key contexts	Examples of supporting data	Additional insights from stakeholders
**3.1. Supporting choice and personalisation of care** *If digital consultations are clearly presented to women as a choice within a hybrid model of care, [**C**] then women will be reassured [**M**] about the option to still have face-to-face appointments when necessary. Furthermore, if the use of digital consultations [**I**] is personalised [**M**] to women's needs, preferences and life circumstances [**C**], women can feel a sense of safety and empowerment [**M**]. This can help digital consultations to be accepted as a valuable addition to traditional maternity care [**O**].*	n=36<br>3, 5, 8, 9, 12, 15, 17-19, 22, 26, 35, 38, 43, 45, 57-71	• All women require support with choice and personalisation, however those who are first-time mothers, have mental health conditions, come from marginalised backgrounds, had previously complicated pregnancies/births, are high risk, face language barriers or have low levels of health literacy may be less suitable for DC-CON and need extra support.<br>• Women's willingness to use DC-CON may depend on whether they have a straightforward or complicated pregnancy, feel uncomfortable on video or prefer telephone	• "So the Asian women that we were looking after prior to lockdown who've stayed on as clients, all, one hundred per cent of them, told us they didn't need our support during lockdown. And the only reason that I can guess that was just because they didn't have space to talk to us […] They've said that they wanted to come back when we do face to face again, but they didn't want support via telephone, video or any online activity."[12] MW, UK.<br>• Some identified their experiences of care as having an impact on their emotional and psychological wellbeing […] "I have been suffering from postnatal depression and have felt that phone calls have simply not been	• DC-CONs should be presented as a choice for women, never mandatory, and based on an assessment of individual women's needs, preferences and circumstances.<br>• Women's consultation preferences and digital resources should be discussed and recorded early on in the pregnancy, including any adjustments they might need to make the most of DC-CONs.<br>• At present, in the NHS, some antenatal 'booking' appointments are conducted via telephone and women are not routinely asked about their consultation mode

Programme Theories: Personalisation & Flexibility for Women	References	Key contexts	Examples of supporting data	Additional insights from stakeholders
		calls, and whether they find DC-CON easy to use.<br>• A few studies identified that those most receptive to DC-CON were often white, young, married and multiparous due to health disparities, language and access barriers, as well as multiparous women potentially having fewer concerns to discuss or greater inconvenience attending face-to-face appointments.	sufficient to support me during this time."[45] SU, UK.<br>• "I feel nervous about lack of face-to-face appointments. I have been having at home visits from an independent Midwife. Our first son was stillborn at 22 weeks, so I feel I need face to face appointments to check the baby and me."[58] SU, UK.<br>• "I think there could be benefits for the right people who are comfortable enough and confident enough and asking the right questions over, over video and things like that."[5] SU, USA.	preferences or digital capacity. If this appointment is in-person, the HCP can better assess preferences, suitability and needs for DC-CONs.<br>• Some women might actively choose DC-CON over in-person care, because they have mental health conditions that make it difficult to leave the house, feel stigmatised (e.g. around smoking), or are uncomfortable in clinical settings. For some women, DC-CON may improve engagement.
**3.2. Managing the burden of care**<br>*If digital consultations are easy to use and fit flexibly [M] with women's preferences, life circumstances and clinical needs [C], it gives them more control over the time, money and effort they have to engage with care [M]. This can be a relief and for some women will make it less burdensome [M] for them to engage with services [O]. It can also make it easier [M] for women to access services/specialists in a wider*	n=43<br>1, 8, 11-13, 15, 17-19, 22-24, 26-31, 34, 37, 39-41, 43, 45, 46, 54, 57, 60-62, 64, 66, 70-79	• Women in remote/rural locations without local access to care – particularly specialist care – who would otherwise incur time and financial costs to be seen in-person, or potentially forgo care all together.<br>• Women with co-morbidities that made travelling difficult, women who needed frequent monitoring (e.g. for GDM), women juggling other responsibilities (e.g. childcare or work) and	• "It's [remote care] flexible, so if I'm, like, feeling tired or unwell, I can just stay at home and still get the same level of care."[12] SU, UK.<br>• "The expertise is there because the doctor's there on screen. You can ask a question without having to think well is he going to be able to answer this, is he not going to be able to answer it .. . You know the right professional's there."[1] SU, UK.<br>• Some clinicians reported that telehealth utilisation reduced the number of women who failed to attend appointments and	• The potential for DC-CON to reduce 'did not attend' rates was considered especially important for vulnerable women with complex social risk factors.

Programme Theories: Personalisation & Flexibility for Women	References	Key contexts	Examples of supporting data	Additional insights from stakeholders
*geographical area, potentially improving clinical outcomes [**O**].*		potentially women in early labour who could be supported remotely to stay at home longer.	improved their ability to engage with "harder to reach" women who "often fall through the cracks". Their rationale was that telehealth is more convenient and minimises barriers to attending appointments such as effort, costs and time.[15] AUS.<br>• "I found it much easier to just be able to be at home, not have to worry about getting the kids ready and long care rides or have to worry about findings someone to watch them. They were very good if I needed to take care of the baby for a second or breastfeed."[75] SU, CAN.	

Programme Theory Domain 4: Women's Access and Inclusion

Programme Theories: Women's Access and Inclusion	References	Key contexts	Examples of supporting data	Additional insights from stakeholders
**4.1. Supporting women's knowledge and navigation of care**<br>*When comprehensive information on digital consultations is provided to women in an easy to understand, accessible format and in a variety of languages, it can facilitate health and digital literacy [**C**]. If women are made*	n=31<br>1-3, 5, 9, 10, 12-15, 17, 23, 24, 28, 30, 33, 34, 38, 41, 43, 45, 46, 54, 57, 60, 68, 80-83	• Women who are unaware of DC-CON as a potential option in their maternity care, and the advantages/ disadvantages of DC-CON compared to in-person care.<br>• Women who have not used DC-CON before or who are anxious about using DC-CON. In	• "That these women are informed thoroughly about the service [video call service for early labour] they are about to use, that they are maybe briefed during late pregnancy appointments. At the front of their chart, there should be a privacy consent form, information on how the service is managed, who makes the phone calls and from where. They have	• Since DC-CON is normalised in other areas of healthcare, such as primary care, some felt it made sense to offer DC-CON in maternity care too.<br>• Others noted that just because women use telephone and video calls in their personal life, it does not necessarily mean that

Programme Theories: Women's Access and Inclusion	References	Key contexts	Examples of supporting data	Additional insights from stakeholders
*aware of the different types of consultations available to them when they first engage with the maternity services [**C**], they can be empowered [**M**] to make informed choices about the mode of care they receive [**M**]. This will improve the potential for personalisation [**M**] of care delivery, enable access [**O**] and help women to play an active role in their maternity care [**O**].*		particular, women with mental health problems, lacking digital literacy, facing communication barriers or a shy/inhibited disposition.<br>• Women unfamiliar with NHS maternity care and/or low health literacy in general.	to be fully aware of what they are going to do"[2] MW, UK/IT.<br>• "If it [DC-CON] was a longer term thing where we were talking about bringing in remote care as part of standard maternity then that should be communicated to you right at the beginning as part of your package of care."[12] HCP, UK.<br>• "I mean I would think a dry run with your patient would be necessary… 'let's practice this; I want you to go into another room and I want you to video me. You know, so that way you know it works.' Every technology there's always hiccups"[23] MW, UK/USA.<br>• "I mean these days we Skype or Facetime, you know, within your personal life so why, why shouldn't it be used for like you know, something medical?" [1] SU, UK.	they are comfortable having medical appointments this way.<br>• A personalised approach to DC-CON based on women's individual needs and preferences is key.<br>• When women are confused about who to call for help they might contact their GP surgery who then becomes the 'gatekeepers' to maternity care. However, stakeholders stressed that going through busy GP surgeries was often time-consuming and complicated, potentially delaying access to care.
**4.2. Ensuring inclusion and equity**<br>*Whilst there can be benefits to using digital clinical consultations [**I**], for women who face language or other communication barriers [**C**], digital clinical consultations [**I**] can present a challenge to the equitable access of care [**O**]. Experiencing communication*	n=32<br>2, 3, 10-12, 14, 15, 17, 23, 24, 28, 29, 31, 32, 34, 38, 41-43, 45-48, 54, 56-58, 63, 79, 84-86	• Women for whom English is not their first language, have disabilities (including learning disabilities, hearing or visual impairments) or who are neurodiverse and may at times struggle to communicate.<br>• HCPs who care for the women above, and their	• "Women that don't necessarily speak good English or limited English, it [video] would maybe be a little bit better for them as well. Because at least then they could physically see you and then maybe you could use hand gestures to kind of help."[2] MW, UK/IT.<br>• "I find it hard sometimes depending on the accent to	• Staff do not always know an interpreter is needed until the women arrives for her appointment, by which time it is often too late to arrange; highlighting the importance of effective administrative systems.<br>• Involving family members in interpretation may not be appropriate depending on

Programme Theories: Women's Access and Inclusion	References	Key contexts	Examples of supporting data	Additional insights from stakeholders
*barriers can create frustration or anxiety, a lack of motivation or sense of entitlement [**M**] to engage with care [**O**]. This can lead to particular groups of women receiving less or inappropriate care relative to their needs [**O**], important issues being missed and sub-optimal clinical outcomes [**O**].*		access to resources to overcome barriers e.g. interpretation services.	follow through, so I felt like it was really…she was talking really fast, and maybe I could have said, like, for…ask for her to slow down a little bit. But, yeah, I think that the main barrier was actually getting a bit lost in translation, 'cause at the end of the call, for example, I didn't even realise the call was about to end (laugh) […] And then I realised I hadn't asked any of my questions"[12] SU, UK.<br>• One woman was deaf and relied on lip reading. While she was engaged in the video consultation, she gave up halfway and became upset as the lag time made it impossible for her to lip read.[24] UK.<br>• It was evident that virtual consultations, either by video or telephone, meant that staff were not aware of the lack of understanding. An inability to speak English as a first language may be a contraindication to remote consultations and guidance reflects this […] stat[ing] that face to face treatment may be preferable when it is hard to ensure, by remote means, that people have all the information they want and	the topic of conversation, especially safeguarding concerns.<br>• Real-time digital translation could be a useful back-up option when interpreters are not available.<br>• Even where a woman speaks an understandable level of English as a second language, DC-CON could create anxiety and worries about either not understanding the HCP or not being understood by the HCP; this could be especially troublesome if the staff member had a strong regional UK accent.<br>• Neurodiverse women could also experience anxiety and potentially a lack of engagement with care (for example not answering the phone) or reliance on partners/family to communicate on their behalf if the consultation modality was not suited to their communication needs.

Programme Theories: Women's Access and Inclusion	References	Key contexts	Examples of supporting data	Additional insights from stakeholders
			need about treatment options.[87] UK.	
**4.3. Considering access to digital resources**<br>*If women do not have access to digital devices, a reliable internet connection or telephone signal [**C**], it may lead to feelings of disempowerment, frustration and loneliness [**M**] as women will struggle to engage with digital clinical consultations [**O**]. This is likely to disproportionately affect already vulnerable women living in poverty or unstable circumstances [**C**], exacerbating health inequalities through digital exclusion [**O**].*	n=26<br>3, 10, 12, 13, 15, 17, 19, 20, 23, 24, 28, 31, 34, 38, 41, 46, 53, 54, 57, 62, 66, 69, 74, 75, 82, 84	• Women of low socio-economic status without consistent access to digital devices, WiFi, phone signal, credit/data, charging facilities and remote monitoring equipment (if necessary).<br>• Women in remote/rural areas with poor connectively, those experiencing poverty (including digital poverty) and those in unstable housing such as migrants, refugees and asylum seeks.	• "I mean, the video calls are a bit of an issue, just because of the internet connection, and I think…I mean, I'm not 100 per cent sure but I…so I…I'm in a very rural area, I don't have broadband, I'm relying on my 4G hotspot, so that is a bit of a problem."[12] SU, UK.<br>• "There's constantly a push for things to be digital; and there are huge advantages of that, but, until you make internet free for everyone and give everyone a smart phone, then, you know, the people that really need us are the ones that get left behind."[12] HCP, UK.<br>• "My cell phone, it has limited data, so I'm not really able to video chat much. When I do it with my family, I do it a few times and that's pretty much it. Then it'll start like freezing or coming on saying low data. So just not being able to have like the actual access to kind [of] do it and video chat. . . it's hard."[38] SU, USA.<br>• Ninety-three survey respondents answered the survey question "are there things that make telemedicine visits hard?" […] Of these, 39.8% cited poor internet	• For very vulnerable women even accessing a phone, purchasing credit and having a consistent phone number can be difficult, posing barriers to contact.

Programme Theories: Women's Access and Inclusion	References	Key contexts	Examples of supporting data	Additional insights from stakeholders
			or phone connectivity and 10.8% reported not having the right equipment.[66] USA.	

Programme Theory Domain 5: Quality Care through Relationship-Focused Connections

Programme Theories: Quality Care through Relationship-Focused Connections	References	Key contexts	Examples of supporting data	Additional insights from stakeholders
**5.1. Promoting safety and managing risk**<br>*Digital clinical consultations [I] provide staff with additional methods with which to communicate with women [C]. When healthcare professionals are matching the mode of consultation to the reason for consultation [C], understanding [M] women's physical, psychological or social circumstances and risks [C] can help staff to personalise care and manage uncertainty [M]. This can lead to equivalent clinical outcomes [O], and safety assurances [O].*	n=51<br>2, 3, 5, 9-20, 23, 24, 26-28, 30-32, 34, 38, 41, 45, 46, 48-50, 53-55, 57, 58, 62, 67-69, 73, 79, 82, 84, 86, 88-92	• When there is a lack of physical examination and non-verbal communication in a consultation (such as via DC-CON) it can affect women and staff's confidence levels.<br>• DC-CON was perceived to suit low-risk women, multiparous women, those not requiring examination, and those living far from hospital. Those not suited included high-risk women, those facing communication barriers, those at risk of social isolation (including asylum seekers, refugees and young mothers) and those with mental health considerations, safeguarding concerns or other psychosocial issues.	• "Videocalls definitely have the advantage of letting you see the woman, how she moves, how she acts, where and who she is. There are a lot of visual elements we can use to make an assessment, whereas on the phone, you can only rely on the voice for clues: when the woman is quiet, there is a contraction but […] you can't see how her body is reacting"[2] MW, UK/IT.<br>• "More often than not their partner didn't come, and so it provided a safe space for women to talk about their issues at home. And enabled us to pick on subtleties in terms of any domestic abuse, any physical abuse; you know, you'd sometimes be able to see that physically on their body. So, you don't necessarily see that remotely."[12] HCP, UK.<br>• "It is hard to make contact on the telephone, you cannot	• Some obstetrician stakeholders commented that they felt the clinical risk of missing something via DC-CON was probably not any more likely than in-person care.<br>• For women in difficult home situations, including domestic violence, in-person appointments were an important opportunity to create a safe, private space in which women could talk openly and seek help.<br>• For DC-CON a cautious approach should be taken centring on women's own preferences, comprehensive safety-netting and clear clinical guidance and protocols to make sure no-one falls through the cracks.

Programme Theories: Quality Care through Relationship-Focused Connections	References	Key contexts	Examples of supporting data	Additional insights from stakeholders
		• Women and staff considered that DC-CONs could be well-suited to 'transactional' care where physical examination was not needed (e.g. form filling, test results, regular monitoring, review appointments) and less suited to discussion of sensitive issues.<br>• Telephone triage could play a vital role in promoting safety and managing risk so long as those answering the phone were appropriately qualified and experienced.	communicate fully if you can't use body language as well. Also, the midwife cannot see you so cannot examine you properly"[58] SU, UK.<br>• 75% agreed that the lack of physical examination was not a problem. Thus, 67% of providers agreed that telemedicine visits are an adequate replacement to in-person visits and 83% agreed they would like telehealth to be an option for future obstetrical visits.[26] USA.<br>• […] recognising situations in which remote consultations are inadequate. This may be for several reasons including language difficulties, lack of access to appropriate technology, repeated presentation, clinical complexity or potentially severe/high risk conditions.[32] UK.	
**5.2. Managing relationships and building rapport**<br>*If digital consultations are used in place of face-to-face care, it can affect the women-healthcare provider relationship [**C**]. Since video calls enable the conveyance of non-verbal cues [**M**], they can be more beneficial in relationship building than*	n=46<br>1-3, 5, 8-15, 17-21, 23, 24, 27, 29-31, 34, 36, 38, 43, 45, 46, 55, 57, 58, 60-63, 66, 69-71, 74, 79, 80, 82, 88, 93	• Continuity of carer was considered particularly important because it was thought to be more difficult to assess women online and to build rapport remotely, particularly because of the more transactional, rather than	• "If you're seeing the same midwife, even on a video call, it makes you feel even more reassured. […] I do think that implementing this with the continuity teams and see how that works with them, I think that would be good."[2] MW, UK/IT.<br>• "You know, midwifery is a science, but it is also an art, and	• It is more comfortable being seen by and showing a known HCP what was wrong online, highlighting how the sometimes awkward nature of video calls could pose a barrier to women appearing for appointments (a core feature of candidacy).

Programme Theories: Quality Care through Relationship-Focused Connections	References	Key contexts	Examples of supporting data	Additional insights from stakeholders
*telephone calls [**O**]. If a relationship of trust has already been established and there is sufficient time for the consultation [**C**], then staff and women can communicate easily and openly [**M**], improving women's disclosure of sensitive information and feelings of reassurance [**M**]. For both routine and complex care via digital consultations, continuity of carer can lead to greater satisfaction for women and professionals and is perceived to support optimal clinical outcomes [**O**].*		therapeutic, nature of DC-CON.<br>• It could be particularly beneficial for women and providers to establish a relationship in-person before starting virtual care.<br>• Establishing a relationship between a women and HCP can be particularly important when delivering of bad news and emotionally supporting women via DC-CON.	it relies on our being together and picking up on people's communication skills, their…you know, their social situations, their body language, the relationships they have, you can't pick that up on a video."[13] HCP, UK.<br>• "[If] I'd had a video call with a lady and then I saw her come into the door … I'd feel like I knew her already … and I'd already started to build up that relationship…"[23] MW, UK/USA.<br>• "I think it might be challenging if there was an intimate something that we would need to converse with via Facetime or what have you. I mean, I'm not concerned with the phone call being hacked and all of a sudden, my genitals being online or something silly like that . . . I don't have a concern with that necessarily. But I think if you already had a relationship with the midwife you were working with, that level of comfortability and privacy would have hopefully already been there."[9] SU, USA.<br>• "I feel like maybe it's better in person because when you can see somebody's facial expression or how they react to a certain question or comment,	• Particularly vulnerable women, the midwife-women relationship could be a significant source of support, sometimes viewed by the women more like a friendship than a professional relationship.<br>• Stakeholders added that a DC-CON with a healthcare professional who was engaged and made a connection with the women could be more beneficial than an in-person appointment where the professional seemed disengaged and was looking at their computer more than the women next to them.

Programme Theories: Quality Care through Relationship-Focused Connections	References	Key contexts	Examples of supporting data	Additional insights from stakeholders
			that probably tells you more about the question you're asking than someone's actual answer."[38] SU, USA.	
**5.3. Supporting women's empowerment and familial involvement**<br>*If women have the ability to use digital consultations [**C**], it can make it easier to facilitate women's active participation [**M**] in partnership with their healthcare provider, especially if remote monitoring is utilised [**C**]. The flexibility and convenience of digital consultations [**C**] can also help to include women's partners/families [**M**] in their care. This can empower, motivate and give women a sense of control over their health and care, [**M**] improving access and enhancing engagement with services [**O**].*	n=27<br>2, 3, 5, 8, 12, 15, 28, 30-32, 34, 36, 38, 40, 43, 46, 52-54, 57, 62, 69, 75, 76, 78, 82, 89	• Women who need regular monitoring e.g. for GDM or high blood pressure, and are able to conduct this themselves at-home.<br>• Women who have the resources and are confident and comfortable using at-home monitoring equipment.<br>• Partners and family members whose involvement in the women's maternity care would benefit from the flexibility of DC-CON. This was particularly true during Covid-19 when restrictions meant that women may have to attend appointments or give birth alone.	• "We have that battle calls where the partner calls and we're like, 'But we want to speak to the woman' and actually, the woman don't always want to speak to you, they've asked their partner to call on their behalf. So, it actually would make it a bit more family centred if you're having a video call with the woman and the partner"[2] MW, UK/IT.<br>• "She [HCP] said she'd send us home with Dopplers to listen to the baby's heart at home, which is like, 'Woah. I'm supposed to sit there and try to find it?' I can't even think about that. So, it's definitely weird and different and not what I expected at all. It makes me nervous that the doctor won't be right there to do it for me, like someone who went to school for this and is trained in this."[8] SU, USA.<br>• "… [the midwife] explained the use of the equipment and took us through all the steps of the entire process. For me it was really nice to speak to somebody on the phone every single day. In	• Despite the convenience of at-home monitoring, some women may still feel more reassured if this is done by a professional and therefore prefer to visit the maternity unit in-person.

Programme Theories: Quality Care through Relationship-Focused Connections	References	Key contexts	Examples of supporting data	Additional insights from stakeholders
			my experience they would call quickly after sending the CTG [cardiotocography]"[78] SU, NLD.<br>• "Telehealth in pregnancy can be tricky. We have to trust the patient to tell us exactly what is going on and trust in their BP [blood pressure] cuffs at home. Things can easily be missed in pregnancy with telehealth visits" [54] RN, USA.	
**5.4. Offering connection and support**<br>*If digital consultations can provide additional and/or convenient opportunities for women to connect with services and staff [**C**] it can support women's sense of safety, reassurance and empowerment [**M**]. These benefits may be enhanced by a pre-existing healthcare provider-woman relationship, good communication and sufficient time for the consultation [**C**]. This leads to increased self-efficacy and motivation [**M**] contributing to satisfaction, engagement and access [**O**].*	n=18<br>2, 3, 9, 12, 15, 22-24, 32, 36, 42, 43, 45, 60, 61, 75, 78, 80	• Women who might need out-of-hours care as DC-CON can offer staff more flexible working patterns and therefore increase access.<br>• For women who worry about burdening or bothering healthcare services, DC-CON can help them to feel more entitled to care – this can be particularly important for vulnerable women in difficult personal situations (Evans, 2017; Rayment-Jones, 2022; Baron, 2018).<br>• HCPs delivering DC-CON who have expectations about the appropriate environment in which	• "So we can be a lot more responsive to these women, by literally just picking up the phone and having that chat with them. You don't have the practical issues, is there a clinic room available, how long is it going to take her to come in, I haven't got a clinic slot for 3 weeks"[12] MW, UK.<br>• "[…] Whoever would answer the phone was reassuring, they were able to talk me through things… if we didn't have the like phone number that we could call the first few weeks it would have been a lot worse, a lot more difficult. We would have ended up in A&E a lot more often than we did"[42] SU, UK.<br>• "As a mom, you have so many questions about is this normal, is this working right and you don't	• When women do not seem ready or focussed on the DC-CON, HCPs worry about how engaged they are in the appointment, their ability to take in information and ask questions.<br>• Women could become frustrated if their HCP did not call at the expected time.

Programme Theories: Quality Care through Relationship-Focused Connections	References	Key contexts	Examples of supporting data	Additional insights from stakeholders
		women should receive DC-CONs.	really know who to turn to with those questions. I'm quite a worrier so I worry about a lot of things. It's just nice to have that. It's a little less formal. You don't feel like you're taking up a lot of time. You don't have to book an appointment just to get one quick question answered"[75] SU, CAN.<br>• Pre-admission telephone triage provides the gateway for women and pregnant people to raise concerns and allows healthcare staff to identify whether there is a need for a person to attend the maternity unit […] The variability in how information is conveyed over the telephone is influenced by the style of communication. How the clinician receives the information is influenced by their knowledge of the subject in the context of the healthcare environment.[32] UK.	

References

1. Bidmead E, Lie M, Marshall A, et al. Service user and staff acceptance of fetal ultrasound telemedicine. *Digit Health* 2020; 6: 2055207620925929. DOI: https://dx.doi.org/10.1177/2055207620925929.

2. Borrelli S, Downey, J., Fumagalli, S., Colciago, E., Antonella, N., Spiby, H. How should a video-call service for early labour be provided? A qualitative study of midwives' perspectives in the United Kingdom and Italy. *Women Birth* 2023. DOI: https://doi.org/10.1016/j.wombi.2023.06.00.

3. Borrelli S, Downey J, Colciago E, et al. Mothers' perspectives on the potential use of video-calling during early labour in the United Kingdom and Italy: A qualitative study. *Women Birth* 2023. DOI: https://doi.org/10.1016/j.wombi.2023.01.004.

4. Cordasco KM, Katzburg JR, Katon JG, et al. Care coordination for pregnant veterans: VA's Maternity Care Coordinator Telephone Care Program. *Translational Behavioral Medicine* 2018; 8: 419-428. DOI: https://dx.doi.org/10.1093/tbm/ibx081.

5. Craighead CG, Collart C, Frankel R, et al. Impact of Telehealth on the Delivery of Prenatal Care During the COVID-19 Pandemic: Mixed Methods Study of the Barriers and Opportunities to Improve Health Care Communication in Discussions About Pregnancy and Prenatal Genetic Testing. *JMIR Formative Research* 2022; 6: e38821. DOI: https://dx.doi.org/10.2196/38821.

6. Engeltjes B, Rosman A, Scheele F, et al. Evaluation of Normalization After Implementation of the Digital Dutch Obstetric Telephone Triage System: Mixed Methods Study With a Questionnaire Survey and Focus Group Discussion. *JMIR Formative Research* 2022; 6: e33709. DOI: 10.2196/33709.

7. Engeltjes B, Wouters E, Rijke R, et al. Obstetric telephone triage. *Risk Manage Healthc Policy* 2020; 13: 2497-2506. DOI: http://dx.doi.org/10.2147/RMHP.S277464.

8. Farrell R, Collart C, Craighead C, et al. The Successes and Challenges of Implementing Telehealth for Diverse Patient Populations Requiring Prenatal Care During COVID-19: Qualitative Study. *JMIR Formative Research* 2022; 6: e32791. DOI: https://dx.doi.org/10.2196/32791.

9. Faucher MA and Kennedy HP. Women's Perceptions on the Use of Video Technology in Early Labor: Being Able to See. *Journal of Midwifery & Women's Health* 2020; 65: 342-348. DOI: https://dx.doi.org/10.1111/jmwh.13091.

10. Galle A, Semaan A, Huysmans E, et al. A double-edged sword-telemedicine for maternal care during COVID-19: findings from a global mixed-methods study of healthcare providers. *BMJ Glob Health* 2021; 6. DOI: 10.1136/bmjgh-2020-004575.

11. Henry A, Yang J, Grattan S, et al. Effects of the COVID-19 Pandemic and Telehealth on Antenatal Screening and Services, Including for Mental Health and Domestic Violence: An Australian Mixed-Methods Study. *Frontiers in Global Women's Health* 2022; 3. Original Research. DOI: 10.3389/fgwh.2022.819953.

12. Hinton L, Dakin FH, Kuberska K, et al. Quality framework for remote antenatal care: qualitative study with women, healthcare professionals and system-level stakeholders. *BMJ Quality & Safety* 2022; 12: 12. DOI: 10.1136/bmjqs-2021-014329.

13. Hinton L, Kuberska K, Dakin F, et al. A qualitative study of the dynamics of access to remote antenatal care through the lens of candidacy. *J Health Serv Res Policy* 2023. DOI: 10.1177/13558196231165361.

14. Klamroth-Marganska V, Gemperle M, Ballmer T, et al. Does therapy always need touch? A cross-sectional study among Switzerland-based occupational therapists and midwives regarding their experience with health care at a distance during the COVID-19 pandemic in spring 2020. *BMC Health Services Research* 2021; 21: 578. DOI: https://dx.doi.org/10.1186/s12913-021-06527-9.

15. Kozica-Olenski SL, Soldatos G, Marlow L, et al. Exploring the acceptability and experience of receiving diabetes and pregnancy care via telehealth during the COVID-19 pandemic: a qualitative study. *BMC Pregnancy and Childbirth* 2022; 22: 932. DOI: https://dx.doi.org/10.1186/s12884-022-05175-z.

16. Krenitsky NM, Spiegelman J, Sutton D, et al. Primed for a pandemic: Implementation of telehealth outpatient monitoring for women with mild COVID-19. *Seminars in Perinatology* 2020; 44: 151285. DOI: https://dx.doi.org/10.1016/j.semperi.2020.151285.

17. Mehl SC, Short WD, Powell P, et al. Impact of Telemedicine on Prenatal Counseling at a Tertiary Fetal Center: A Mixed Methods Study. *The Journal of Surgical Research* 2022; 280: 288-295. DOI: https://dx.doi.org/10.1016/j.jss.2022.07.020.

18. Quinn LM, Olajide O, Green M, et al. Patient and Professional Experiences With Virtual Antenatal Clinics During the COVID-19 Pandemic in a UK Tertiary Obstetric Hospital: Questionnaire Study. *J Med Internet Res* 2021; 23: e25549. DOI: 10.2196/25549.

19. Rasekaba T, Nightingale H, Furler J, et al. Women, clinician and IT staff perspectives on telehealth for enhanced gestational diabetes mellitus management in an Australian rural/regional setting. *Rural and Remote Health* 2021; 21: 5983. DOI: https://dx.doi.org/10.22605/RRH5983.

20. Reid CN, Marshall J and Fryer K. Evaluation of a Rapid Implementation of Telemedicine for Delivery of Obstetric Care During the COVID-19 Pandemic. *medRxiv* 2021: 2021.2005.2019.21257311. DOI: 10.1101/2021.05.19.21257311.

21. Rousseau A, Gaucher L, Gautier S, et al. How midwives implemented teleconsultations during the COVID-19 health crisis: a mixed-methods study. *BMJ Open* 2022; 12: e057292. DOI: https://dx.doi.org/10.1136/bmjopen-2021-057292.

22. Shashikumar A, Okesene-Gafa K, Apaapa-Timu T, et al. Teleclinics for the management of diabetes in pregnancy during COVID-19 —maternal satisfaction and pregnancy outcomes. *New Zealand Medical Journal* 2022; 135: 63-77.

23. Spiby H, Faucher MA, Sands G, et al. A qualitative study of midwives' perceptions on using video-calling in early labor. *Birth* 2019; 46: 105-112. DOI: 10.1111/birt.12364.

24. Tavener CR, Kyriacou C, Elmascri I, et al. Rapid introduction of virtual consultation in a hospital-based Consultant-led Antenatal Clinic to minimise exposure of pregnant women to COVID-19. *BMJ Open Qual* 2022; 11: e001622. DOI: 10.1136/bmjoq-2021-001622.

25. Theiler RN, Butler-Tobah Y, Hathcock MA, et al. OB Nest randomized controlled trial: a cost comparison of reduced visit compared to traditional prenatal care. *BMC Pregnancy and Childbirth* 2021; 21: 71. DOI: 10.1186/s12884-021-03557-3.

26. Tozour JN, Bandremer S, Patberg E, et al. Application of telemedicine video visits in a maternal-fetal medicine practice at the epicenter of the COVID-19 pandemic. *American Journal of Obstetrics & Gynecology MFM* 2021; 3: 100469. DOI: https://dx.doi.org/10.1016/j.ajogmf.2021.100469.

27. Zulifqar BA. *Providers' Satisfaction with Provision of Prenatal Care During the COVID-19 Pandemic*. M.S., University of North Texas Health Science Center at Fort Worth, Ann Arbor, 2021.

28. Almuslim H and AlDossary S. Models of Incorporating Telehealth into Obstetric Care During the COVID-19 Pandemic, Its Benefits And Barriers: A Scoping Review. *Telemed J E Health* 2022; 28: 24-38. DOI: 10.1089/tmj.2020.0553.

29. Chua CMS, Mathews J, Ong MSB, et al. Use of telelactation interventions to improve breastfeeding outcomes among mothers: A mixed-studies systematic review. *Women Birth* 2022. DOI: https://dx.doi.org/10.1016/j.wombi.2022.06.011.

30. Konnyu KJ, Danilack VA, Adam GP, et al. Changes to Prenatal Care Visit Frequency and Telehealth: A Systematic Review of Qualitative Evidence. *Obstetrics and Gynecology* 2023. DOI: https://dx.doi.org/10.1097/AOG.0000000000005046.

31. Society for Maternal-Fetal Medicine, Healy A, Davidson C, et al. Society for Maternal-Fetal Medicine Special Statement: Telemedicine in Obstetrics - Quality and Safety Considerations. *American Journal of Obstetrics and Gynecology* 2022. DOI: https://dx.doi.org/10.1016/j.ajog.2022.12.002.

32. Healthcare Safety Investigation Branch. *National Learning Report Intrapartum Stillbirth: Learning from Maternity Safety Investigations that Occurred during the COVID-19 Pandemic, 1 April to 30 June 2020*. 2021. https://hsib-kqcco125-media.s3.amazonaws.com/assets/documents/HSIB_Intrapartum_Stillbirth_Report_web.pdf [accessed 14/02/23]

33. Healthcare Safety Investigation Branch. *Assessment of Risk during the Maternity Pathway*. 2023. https://www.hsib.org.uk/investigations-and-reports/assessment-risk-during-maternity-pathway/report/#43-risk-assessment-and-triage [accessed 16/03/23]

34. Ghimire S, Martinez S, Hartvigsen G, et al. Virtual prenatal care: A systematic review of pregnant women's and healthcare professionals' experiences, needs, and preferences for quality care. *International Journal of Medical Informatics* 2023; 170: 104964. DOI: https://doi.org/10.1016/j.ijmedinf.2022.104964.

35. Appelman IF, Thompson SM, van den Berg LMM, et al. It was tough, but necessary. Organizational changes in a community based maternity care system during the first wave of the COVID-19 pandemic: A qualitative analysis in the Netherlands. *PLOS One* 2022; 17: e0264311. DOI: https://dx.doi.org/10.1371/journal.pone.0264311.

36. Baron AM, Ridgeway JL, Finnie DM, et al. Increasing the Connectivity and Autonomy of RNs with Low-Risk Obstetric Patients: Findings of a study exploring the use of a new prenatal care model. *Am J Nurs* 2018; 118: 48-55. DOI: 10.1097/01.NAJ.0000529715.93343.b0.

37. Butler Tobah YS, LeBlanc A, Branda ME, et al. Randomized comparison of a reduced-visit prenatal care model enhanced with remote monitoring. *Am J Obstet Gynecol* 2019; 221: 638.e631-638.e638. DOI: 10.1016/j.ajog.2019.06.034.

38. Gomez-Roas MV, Davis KDM, Leziak K, et al. Postpartum during a pandemic: Challenges of low-income individuals with healthcare interactions during COVID-19. *PLOS One* 2022; 17: e0268698. DOI: https://dx.doi.org/10.1371/journal.pone.0268698.

39. Khalil C. Understanding the Adoption and Diffusion of a Telemonitoring Solution in Gestational Diabetes Mellitus: Qualitative Study. *JMIR Diabetes* 2019; 4: e13661. DOI: https://dx.doi.org/10.2196/13661.

40. Leighton C, Conroy M, Bilderback A, et al. Implementation and Impact of a Maternal-Fetal Medicine Telemedicine Program. *Am J Perinatol* 2019; 36: 751-758. DOI: https://dx.doi.org/10.1055/s-0038-1675158.

41. Madden N, Emeruwa UN, Friedman AM, et al. Telehealth Uptake into Prenatal Care and Provider Attitudes during the COVID-19 Pandemic in New York City: A Quantitative and Qualitative Analysis. *Am J Perinatol* 2020; 37: 1005-1014. DOI: 10.1055/s-0040-1712939.

42. Rayment-Jones H, Harris J, Harden A, et al. Project20: Maternity care mechanisms that improve (or exacerbate) health inequalities. A realist evaluation. *Women Birth* 2022. DOI: https://dx.doi.org/10.1016/j.wombi.2022.11.006.

43. Sarre G, Hyer S, Chauhan-Whittingham P, et al. Patients' experience of antenatal diabetic care during the current COVID-19 pandemic: an exploratory study. *Practical Diabetes* 2021; 38: 23-30. DOI: https://dx.doi.org/10.1002/pdi.2367.

44. Shaw S, Wherton J, Vijayaraghavan S, et al. Advantages and limitations of virtual online consultations in a NHS acute trust: the VOCAL mixed-methods study. *NIHR Journals Library (Health Services and Delivery Research)* 2018. DOI: https://dx.doi.org/10.3310/hsdr06210.

45. Smith AD, Z; Farmer, D; Stacey, T *Using Maternity Services During COVID-19*. 2020. Yorkshire and Harrogate Maternity Voices Partnership; University of Huddersfield https://www.maternityvoices.co.uk/content/uploads/2021/01/Covid-19-Maternity-Report.pdf [accessed 07/07/21]

46. Wu K, Lopez C and Nichols M. Virtual Visits in Prenatal Care: An Integrative Review. *Journal of Midwifery & Women's Health* 2021. DOI: https://dx.doi.org/10.1111/jmwh.13284.

47. Healthcare Safety Investigation Branch. *National Learning Report Maternal Death: Learning from Maternal Death Investigations during the First Wave of the COVID-19 Pandemic*. 2021. https://hsib-kqcco125-media.s3.amazonaws.com/assets/documents/HSIB_Maternal_Death_Report_V13.pdf [accessed 14/02/23]

48. Bailey CM, Newton JM and Hall HG. Telephone triage in midwifery practice: A cross-sectional survey. *International Journal of Nursing Studies* 2019; 91: 110-118. DOI: 10.1016/j.ijnurstu.2018.11.009.

49. Bailey CM, Newton JM and Hall HG. Telephone triage and midwifery: A scoping review. *Women & Birth* 2018; 31: 414-421. DOI: 10.1016/j.wombi.2017.12.002.

50. Friedemann Smith C, Lunn H, Wong G, et al. Optimising GPs' communication of advice to facilitate patients' self-care and prompt follow-up when the diagnosis is uncertain: A realist review of 'safety-netting' in primary care. *BMJ Quality and Safety* 2022; 31: 541-554. DOI: https://dx.doi.org/10.1136/bmjqs-2021-014529.

51. Foster KE, Casola AR, Uzumcu Z, et al. Outpatient maternity care and telemedicine use perceptions in the COVID-19 pandemic: a 2020 CERA survey. *Women & Health* 2022; 62: 402-411. DOI: https://dx.doi.org/10.1080/03630242.2022.2072051.

52. Gemperle M, Grylka-Baeschlin S, Klamroth-Marganska V, et al. Midwives' perception of advantages of health care at a distance during the COVID-19 pandemic in Switzerland. *Midwifery* 2022; 105: 103201. DOI: https://dx.doi.org/10.1016/j.midw.2021.103201.

53. Moltrecht B, Dalton LJ, Hanna JR, et al. Young parents' experiences of pregnancy and parenting during the COVID-19 pandemic: a qualitative study in the United Kingdom. *BMC Public Health* 2022; 22: 523. DOI: https://doi.org/10.1186/s12889-022-12892-9.

54. Talmont E and Vitale TR. Telehealth Readiness Assessment of Perinatal Nurses. *Nursing for Women's Health* 2022; 26: 86-94. DOI: https://dx.doi.org/10.1016/j.nwh.2022.01.004.

55. Fernandez Lopez R, de-Leon-de-Leon S, Martin-de-Las-Heras S, et al. Women survivors of intimate partner violence talk about using e-health during pregnancy: a focus group study. *BMC Women's Health* 2022; 22: 98. DOI: https://dx.doi.org/10.1186/s12905-022-01669-2.

56. Mann C, Goodhue B, Guillard A, et al. The COVID-19 pandemic and reproductive genetic counseling: Changes in access and service delivery at an academic medical center in the United States. *Journal of Genetic Counseling* 2021; 30: 958-968. DOI: 10.1002/jgc4.1462.

57. Peahl AF, Powell A, Berlin H, et al. Patient and provider perspectives of a new prenatal care model introduced in response to the coronavirus disease 2019 pandemic. *American Journal of Obstetrics and Gynecology* 2021; 224: 384.e381-384.e311. DOI: https://doi.org/10.1016/j.ajog.2020.10.008.

58. Aydin E, Glasgow KA, Weiss SM, et al. Expectant parents’ perceptions of healthcare and support during COVID-19 in the UK: A thematic analysis. *medRxiv* 2021. DOI: 10.1101/2021.04.14.21255490.

59. Branwer JGR, D; Jackson, C; Dickerson, J; Dharni, N; Sheard, L; Smith, H; . *“What if I’m on my own?” Interim Report: Experiences of Pregnancy and Birth During the COVID-19 Pandemic*. 2021. Bradford Research https://www.bradfordresearch.nhs.uk/wp-content/uploads/2021/05/BiB-Qualitative-study_Pregnancy-in-COVID_brief-report_FINAL.pdf [accessed 07/07/22]

60. Demirci J, Kotzias V, Bogen DL, et al. Telelactation via Mobile App: Perspectives of Rural Mothers, Their Care Providers, and Lactation Consultants. *Telemedicine Journal and E-health: The Official Journal of the American Telemedicine Association* 2019; 25: 853-858. DOI: https://dx.doi.org/10.1089/tmj.2018.0113.

61. Evans EC and Bullock LFC. Supporting Rural Women During Pregnancy: Baby BEEP Nurses. *MCN: The American Journal of Maternal Child Nursing* 2017; 42: 50-55. DOI: 10.1097/NMC.0000000000000305.

62. Harrison TN, Sacks DA, Parry C, et al. Acceptability of Virtual Prenatal Visits for Women with Gestational Diabetes. *Women's Health Issues* 2017; 27: 351-355. DOI: https://doi.org/10.1016/j.whi.2016.12.009.

63. Karavadra B, Stockl A, Prosser-Snelling E, et al. Women's perceptions of COVID-19 and their healthcare experiences: a qualitative thematic analysis of a national survey of pregnant women in the United Kingdom. *BMC Pregnancy and Childbirth* 2020; 20: 600. DOI: 10.1186/s12884-020-03283-2.

64. Kluwgant D, Homer C and Dahlen H. "Never let a good crisis go to waste": Positives from disrupted maternity care in Australia during COVID-19. *Midwifery* 2022; 110: 103340. DOI: https://dx.doi.org/10.1016/j.midw.2022.103340.

65. Liu CH, Goyal D, Mittal L, et al. Patient Satisfaction with Virtual-Based Prenatal Care: Implications after the COVID-19 Pandemic. *Maternal and Child Health Journal* 2021; 25: 1735-1743. DOI: 10.1007/s10995-021-03211-6.

66. Morgan A, Goodman D, Vinagolu-Baur J, et al. Prenatal telemedicine during COVID-19: patterns of use and barriers to access. *JAMIA Open* 2022; 5: ooab116. DOI: https://dx.doi.org/10.1093/jamiaopen/ooab116.

67. Pflugeisen BM, McCarren C, Poore S, et al. Virtual Visits: Managing prenatal care with modern technology. *MCN The American Journal of Maternal Child Nursing* 2016; 41: 24-30. DOI: https://dx.doi.org/10.1097/NMC.0000000000000199.

68. Sanders J and Blaylock R. "Anxious and traumatised": Users' experiences of maternity care in the UK during the COVID-19 pandemic. *Midwifery* 2021; 102: 103069. DOI: https://doi.org/10.1016/j.midw.2021.103069.

69. Silverio SA, De Backer K, Easter A, et al. Women's experiences of maternity service reconfiguration during the COVID-19 pandemic: A qualitative investigation. *Midwifery* 2021; 102: 103116. DOI: 10.1016/j.midw.2021.103116.

70. Stacey T, Darwin Z, Keely A, et al. Experiences of maternity care during the COVID-19 pandemic in the North of England. *British Journal of Midwifery* 2021; 29: 516-523. DOI: 10.12968/bjom.2021.29.9.516.

71. Sullivan MW, Kanbergs AN, Burdette ER, et al. Acceptability of virtual prenatal care: thinking beyond the pandemic. *The Journal of Maternal-Fetal & Neonatal Medicine* 2021: 1-4. DOI: https://dx.doi.org/10.1080/14767058.2021.1980534.

72. Duryea EL, Adhikari EH, Ambia A, et al. Comparison Between In-Person and Audio-Only Virtual Prenatal Visits and Perinatal Outcomes. *JAMA Network Open* 2021; 4: e215854-e215854. DOI: 10.1001/jamanetworkopen.2021.5854.

73. Oelmeier K, Schmitz R, Moellers M, et al. Satisfaction with and Feasibility of Prenatal Counseling via Telemedicine: A Prospective Cohort Study. *Telemedicine e-Health* 2022; 28: 1193-1198. DOI: 10.1089/tmj.2021.0309.

74. Pflugeisen BM and Mou J. Patient Satisfaction with Virtual Obstetric Care. *Matern Child Health J* 2017; 21: 1544-1551. DOI: 10.1007/s10995-017-2284-1.

75. Saad M, Chan S, Nguyen L, et al. Patient perceptions of the benefits and barriers of virtual postnatal care: a qualitative study. *BMC Pregnancy and Childbirth* 2021; 21: 543. DOI: 10.1186/s12884-021-03999-9.

76. Smith VJ, Marshall A, Lie MLS, et al. Implementation of a fetal ultrasound telemedicine service: women's views and family costs. *BMC Pregnancy and Childbirth* 2021; 21: 38. DOI: https://dx.doi.org/10.1186/s12884-020-03532-4.

77. Sung Y-S, Zhang D, Eswaran H, et al. Evaluation of a telemedicine program managing high-risk pregnant women with pre-existing diabetes in Arkansas's Medicaid program. *Seminars in Perinatology* 2021; 45: 151421. DOI: https://dx.doi.org/10.1016/j.semperi.2021.151421.

78. van den Heuvel JFM, Ayubi S, Franx A, et al. Home-Based Monitoring and Telemonitoring of Complicated Pregnancies: Nationwide Cross-Sectional Survey of Current Practice in the Netherlands. *JMIR mHealth and uHealth* 2020; 8: e18966. DOI: https://dx.doi.org/10.2196/18966.

79. Flaherty SJ, Delaney H, Matvienko-Sikar K, et al. Maternity care during COVID-19: a qualitative evidence synthesis of women's and maternity care providers' views and experiences. *BMC Pregnancy and Childbirth* 2022; 22: 438. DOI: 10.1186/s12884-022-04724-w.

80. Engeltjes B, van Herk N, Visser M, et al. Patients' experiences with an obstetric telephone triage system: A qualitative study. *Patient Education and Counseling* 2023; 108: 107610. DOI: https://doi.org/10.1016/j.pec.2022.107610.

81. Jeganathan S, Prasannan L, Blitz MJ, et al. Adherence and acceptability of telehealth appointments for high-risk obstetrical patients during the coronavirus disease 2019 pandemic. *Am J Obstet Gynecol MFM* 2020; 2: 100233. DOI: 10.1016/j.ajogmf.2020.100233.

82. Moltrecht B, de Cassan S, Rapa E, et al. Challenges and opportunities for perinatal health services in the COVID-19 pandemic: a qualitative study with perinatal healthcare professionals. *BMC Health Services Research* 2022; 22: 1026. DOI: https://dx.doi.org/10.1186/s12913-022-08427-y.

83. Osarhiemen OA, Robinson MA, Zhao Z, et al. Assessing access to obstetrical care via telehealth in the era of COVID-19. *American Journal of Obstetrics and Gynecology* 2022; 226: 429-432. DOI: 10.1016/j.ajog.2021.09.011.

84. Gao C, Osmundson S, Malin BA, et al. Telehealth Use in the COVID-19 Pandemic: A Retrospective Study of Prenatal Care. *Studies in Health Technology & Informatics* 2022; 290: 503-507. DOI: 10.3233/SHTI220127.

85. Khosla K, Suresh S, Mueller A, et al. Elimination of racial disparities in postpartum hypertension follow-up after incorporation of telehealth into a quality bundle. *American Journal of Obstetrics & Gynecology MFM* 2022; 4: 100580. DOI: https://dx.doi.org/10.1016/j.ajogmf.2022.100580.

86. MBRACE-UK. *Saving Lives, Improving Mothers' Care: Lessons learned to inform maternity care from the UK and Ireland Confidential Enquiries into Maternal Deaths and Morbidity 2016-18*. 2021. https://www.npeu.ox.ac.uk/assets/downloads/mbrrace-uk/reports/maternal-report-2020/MBRRACE-UK_Maternal_Report_Dec_2020_v10.pdf [accessed 09/02/21]

87. Knight M, Bunch K, Cairns A, et al. *MBRACE-UK: Saving Lives, Improving Mothers' Care: Rapid report 2021: Learning from SARS-CoV-2-Related and Associated Maternal Deaths in the UK: June 2020-March 2021*. 2021. https://www.npeu.ox.ac.uk/assets/downloads/mbrrace-uk/reports/MBRRACE-UK_Maternal_Report_June_2021_-_FINAL_v10.pdf [accessed 14/02/23]

88. Lapadula MC, Rolfs S, Szyld EG, et al. Evaluating Patients' and Neonatologists' Satisfaction With the Use of Telemedicine for Neonatology Prenatal Consultations During the COVID-19 Pandemic. *Frontiers in Pediatrics* 2021; 9: 642369. DOI: https://dx.doi.org/10.3389/fped.2021.642369.

89. Nelson GA and Holschuh C. Evaluation of Telehealth Use in Prenatal Care for Patient and Provider Satisfaction: A Step Toward Reducing Barriers to Care. *J Nurse Pract* 2021; 17: 481-484. DOI: 10.1016/j.nurpra.2020.12.026.

90. Cantor AG, Jungbauer RM, Totten AM, et al. Telehealth Strategies for the Delivery of Maternal Health Care: A Rapid Review. *Annals of Internal Medicine* 2022; 175: 1285-1297. DOI: https://dx.doi.org/10.7326/M22-0737.

91. MBRACE-UK. *Saving Lives, Improving Mothers' Care Rapid report: Learning from SARS-CoV-2-related and associated maternal deaths in the UK.* 2020. https://www.npeu.ox.ac.uk/assets/downloads/mbrrace-uk/reports/MBRRACE-UK_Maternal_Report_2020_v10_FINAL.pdf [accessed 09/02/21]

92. Palmer KR, Tanner M, Davies-Tuck M, et al. Widespread implementation of a low-cost telehealth service in the delivery of antenatal care during the COVID-19 pandemic: an interrupted time-series analysis. *The Lancet* 2021; 398: 41-52. DOI: https://doi.org/10.1016/S0140-6736(21)00668-1.

93. Fernandez Turienzo C, Rayment-Jones H, Roe Y, et al. A realist review to explore how midwifery continuity of care may influence preterm birth in pregnant women. *Birth* 2021; 48: 375-388. DOI: https://dx.doi.org/10.1111/birt.12547.
